# Associations and mechanisms between childhood adversity and irritability or trait anger across the lifespan: A systematic review and meta-analysis

**DOI:** 10.1016/j.xjmad.2026.100204

**Published:** 2026-09-24

**Authors:** Isabella G. Spaulding, Ruiyu Yang, Grace Cotter, Alexa Duran, Grace Kathman, Murray B. Stein, Charles T. Taylor, Jillian Lee Wiggins

**Affiliations:** a San Diego State University/University of California San Diego Joint Doctoral Program in Clinical Psychology, 6363 Alvarado Ct., San Diego, CA, 92120, USA; b Department of Psychology, San Diego State University, 6363 Alvarado Ct. Suite 250, San Diego, CA, 92120, USA; c Department of Psychiatry, University of California San Diego, 9452 Medical Center Drive, La Jolla, CA, 92037, USA; d School of Public Health, University of California San Diego, 9500 Gilman Drive, La Jolla, CA, 92093, USA

**Keywords:** Irritability, Childhood adversity, Systematic review, Lifespan

## Abstract

**Background:**

Childhood adversity (CA) is a robust predictor of psychopathology later in life and has been linked to numerous transdiagnostic symptom presentations. One such presentation, irritability, has garnered increasing interest given its presence across internalizing and externalizing disorders and associations with greater functional impairment. Preliminary work has demonstrated associations between CA and irritability; however, no systematic review or meta-analysis has examined these associations or the mechanisms by which these constructs are connected.

**Methods:**

PubMed, PsycInfo, and Web of Science were searched from inception to October 2025, for empirical English articles published in peer-reviewed journals that included measures of CA and irritability (or trait anger, a closely related construct) and examined their statistical relationship. All procedures used in this review were consistent with Preferred Reporting Items for Systematic Review and Meta-Analyses (PRISMA) guidelines.

**Results:**

A total of 1839 abstracts were screened, resulting in 208 articles reviewed and 109 articles ultimately included. Random-effects meta-analysis identified a positive relationship between CA and irritability or trait anger in association (pooled association *r* = 0.22, 95% CI [0.19–0.24]) and group comparison (Hedges’ *g* = 0.53, 95% CI [0.35–0.71]) studies across the lifespan. Additionally, the review identified concurrently measured psychological (i.e., emotion dysregulation, narcissism, shame, adult attachment) variables that statistically mediated the relationship between CA and irritability or anger.

**Conclusions:**

Findings support robust associations between early adversity and irritability or trait anger across the lifespan. Limitations and suggestions for future empirical studies are discussed.

## Highlights

• Meta-analysis of associations of irritability or anger with childhood adversity.
• Findings suggest positive association between irritability or anger and adversity.
• Psychological variables mediated childhood adversity-irritability relationship.
• More research is needed to examine causal pathways across lifespan.

## 1 Introduction

Childhood adversity (CA) (e.g., childhood maltreatment, trauma) is one of the most well-established risk factors for the development of psychopathology later in life. Children who experience adversity are twice as likely to develop a psychological disorder [1]. CA is associated with 32% of anxiety disorders, 26% of depressive disorders [2] and increased risk for externalizing disorders, such as conduct disorder and substance abuse [3]. Early adversity is also commonplace, with approximately 60% of youth in the United States experiencing at least one adverse event [4]. Although associations between CA and psychopathology are well-established, relatively limited work has explored the mechanisms by which CA is linked to negative psychological outcomes – a gap partly due to the multifinality of CA, which can lead to both internalizing and externalizing symptoms and disorders. In this context, irritability offers a unique and promising lens as a transdiagnostic symptom and neurobehavioral risk factor of various forms of psychopathology [5,6].

Irritability is commonly conceptualized as elevated proneness to anger relative to peers [7,8] and is one of the top reasons for psychiatric referrals in youth [9]. Early elevated irritability is also a robust predictor of psychopathology (e.g., anxiety, depression, behavioral problems), functional impairment, and suicidality [10,11]. Similarly, in adults, elevated irritability is nearly ubiquitous in multiple disorders (e.g., depression, posttraumatic stress disorder [PTSD], generalized anxiety disorder, bipolar disorder, intermittent explosive disorder) [6,[12], [13], [14]] and is linked with concurrent and future suicidality [15,16]. Given these clinical implications, irritability has gained increasing interest over the last two decades, particularly in pediatric populations, and research has also emerged on its etiology. As elevated irritability is a hallmark symptom of PTSD and trauma exposure itself robustly predicts various forms of psychopathology, exposure to trauma likely predisposes individuals to irritability symptoms. Further, CA may be particularly influential as it occurs during critical developmental periods when emotion regulation and reward and threat processing capacities are developing rapidly [17], which are proposed key mechanisms underlying elevated irritability [8]. Indeed, accumulating work increasingly reveals associations between CA and later irritability [[18], [19], [20], [21], [22]]. A few studies have also examined potential mechanisms, such as emotion regulation in adults [18] and reward processing (e.g., frustrative nonreward) in youth with trauma histories [23]. Of note, although irritability confers elevated risk and functional impairment, it likely also serves as an adaptive reaction (e.g., changes in emotional reactivity and regulation tendencies) to adverse experiences or situations [24]. A clearer understanding of the associations between CA and irritability, as well as potential mechanisms underlying such associations, may improve clinical assessment and treatment efforts (e.g., identifying transdiagnostic treatment targets for trauma-related symptoms, conducting trauma-informed assessment of irritability).

To advance this understanding, consideration of how CA and irritability are empirically defined and measured is critical. Some measurement issues are worth noting. While studies examining downstream outcomes associated with CA are extensive, they vary significantly on their definitions of CA. The majority of studies include experiences of abuse (e.g., physical, sexual, or emotional), neglect (e.g., emotional neglect, chronic poverty, material deprivation), traumatic events, as well as (albeit to a lesser degree) household adversity (e.g., incarcerated parent, parental substance abuse) before the age of 18. Recent work has also contended that structural factors as a result of historical and current inequity should be considered [25,26], such as neighborhood crime rates, school quality, access to healthcare, and environmental pollution [26]. Although structural factors certainly contribute to childhood adversity, we limited our scope in the current review to individual-level CA (e.g., childhood maltreatment and trauma, household dysfunction), as existing models posit that structural factors trickle down to individual-level experiences and we are particularly interested in the individual-level associations and mechanisms between CA and irritability [27]. Studies also vary on how CA is measured, with the majority measuring CA from both binary (e.g., abused versus non-abused) and cumulative perspectives (e.g., total adverse events experienced), though recent dimensional models [28] of adversity recommend examining types of adversity separately (i.e., threat versus deprivation) for more nuanced understanding of its effects on development. In the current review, we included both approaches, as they have all been commonly utilized and provide important insights.

Similarly, there has been a lack of clarity and consistency in how irritability is defined and measured in the existing literature. Although emerging research shows that irritability is likely a distinct construct [29], it has been used interchangeably with closely related concepts, such as anger and aggression, with partly overlapping measurement items (see [30] for a review). Toohey & DiGiuseppe [30] suggest that elevated irritability is a lowered threshold for experiencing negative emotions (e.g., anger, frustration), which may, as a result, be expressed more frequently or intensively. On the other hand, emotions such as anger can be one aspect of how elevated irritability is manifested, but it alone may not fully capture the construct of elevated irritability. In this context, to retain the conceptual distinctiveness of irritability without missing relevant literature that may offer valuable insights (particularly before irritability gained research attention), we included studies examining irritability, as well as its related constructs, as long as they contain a mood component rather than a purely behavioral manifestation (e.g., aggressive behaviors) and reflect a trait (versus state) characteristic that aligns with the proneness-to-anger tendency commonly seen in irritability [31,32]. Accordingly, we explored whether findings differ by measurement. It should be noted that while we opted to examine irritability or trait anger specifically as a clearer understanding of this relationship may offer important insights into developing more precise treatment targets, associations between childhood trauma and a higher-order factor of negative affectivity/neuroticism have been observed [33], which may encompass irritability and trait anger. Thus, the specific associations examined here may point to one specific feature of negative affectivity related to CA.

Collectively, despite emerging empirical evidence suggesting a link between CA and irritability, no systematic evidence synthesis exists in this area. To this end, the current review aimed to identify empirical associations between CA and irritability across developmental stages (e.g., childhood, adolescence, emerging adulthood, adulthood), and identify *how* they associate with each other. Exploring these associations and their linking mechanisms could afford valuable insight into risk identification and treatment development (e.g., trauma-informed mental health care). Moreover, most irritability research focuses on youth (i.e., children and adolescents), and thus, a lifespan approach provides an important synthesis of how CA and irritability are associated beyond childhood. Three objectives of the current review were identified:

1. Does extant empirical evidence support a significant association between CA and irritability?
2. Does the association persist from childhood through adulthood (i.e., across lifespan)?
3. What are the potential mechanisms by which CA and irritability may be associated with each other (i.e., mediators)?

## 2 Methods

### 2.1 Search strategy

This systematic review and meta-analysis was conducted in accordance with the Preferred Reporting Items for Systematic Reviews and Meta-analyses (PRISMA [34]) guidelines. Computerized searches were conducted in the PubMed, APA PsycInfo, and Web of Science databases for published articles up to October 2025. The scope of this review focused on the associations between irritability and CA. Specifically, we were interested in irritability as measured dimensionally, in line with the Research Domain Criteria (RDoC) initiative put forth by the National Institute of Mental Health [35]. We also opted to include searches of manifestations of irritability that are commonly examined in pediatric empirical studies (e.g., tantrums), or as a core feature (i.e., required presence according to the diagnostic criteria) of a psychological disorder when categorical diagnoses are considered (e.g., disruptive mood dysregulation disorder [DMDD]). Given that prior literature has often used irritability and anger interchangeably, we included studies examining irritability, anger, or both for comprehensiveness, as long as they reflected a trait characteristic that contained a mood and/or emotional component rather than exclusively a measure of behavior (e.g., aggression). We were interested in CA as an umbrella term for multiple types of adversity, including childhood trauma and maltreatment (e.g., abuse, neglect). We also opted to include adverse childhood experiences (ACEs) and early life stress broadly defined [36], which may extend to household dysfunction, as well as adversity experienced outside of the immediate home environment (e.g., exposure to community violence). Equivalent search terms were used across databases for title and abstract searches (see Supplemental Materials for details).

There were no date limitations set in search restrictions. After the articles were extracted, computerized removal of duplicate articles was conducted in EndNote, followed by manual detection and removal of duplicate articles. Titles and abstracts from each publication were uploaded to Rayyan, a web-based systematic review tool that assists in the screening process [37]. An additional search was conducted using a snowball method to ensure other relevant articles were not missed [38]. This method included backward (i.e., reviewing references cited by the included studies) and forward (i.e., reviewing articles that cite the included studies) snowballing applied to the reference list of key articles.

### 2.2 Inclusion and exclusion criteria

Articles included in this review met the following criteria: 1) peer-reviewed, empirical studies; 2) available in the English language; 3) included a measure of trait irritability or anger in children or adults (e.g., self-, caregiver-, or teacher-report); (4) included a measure of CA (e.g., self- or caregiver-report, official records); and (5) examined statistical relationships between CA and irritability, including analyses exploring variables mediating their relationship. Studies conducted with non-human samples, and non-empirical articles, including book chapters, case studies/reports, narrative reviews, conference abstracts, study protocols, and clinical guidelines were excluded.

### 2.3 Study selection and data extraction

The first authors screened the titles and abstracts of the articles using the inclusion/exclusion criteria described above. Co-authors then completed a second independent screen using the same inclusion/exclusion criteria, and confirmed the inclusion of articles. Disagreement about the inclusion of articles was handled through a consensus among the authors. Finally, articles meeting the inclusion criteria were retrieved for a full-text review to determine eligibility. Eligible articles were included in the final review. Fig. 1 presents the process of study selection.

**Fig. 1:**
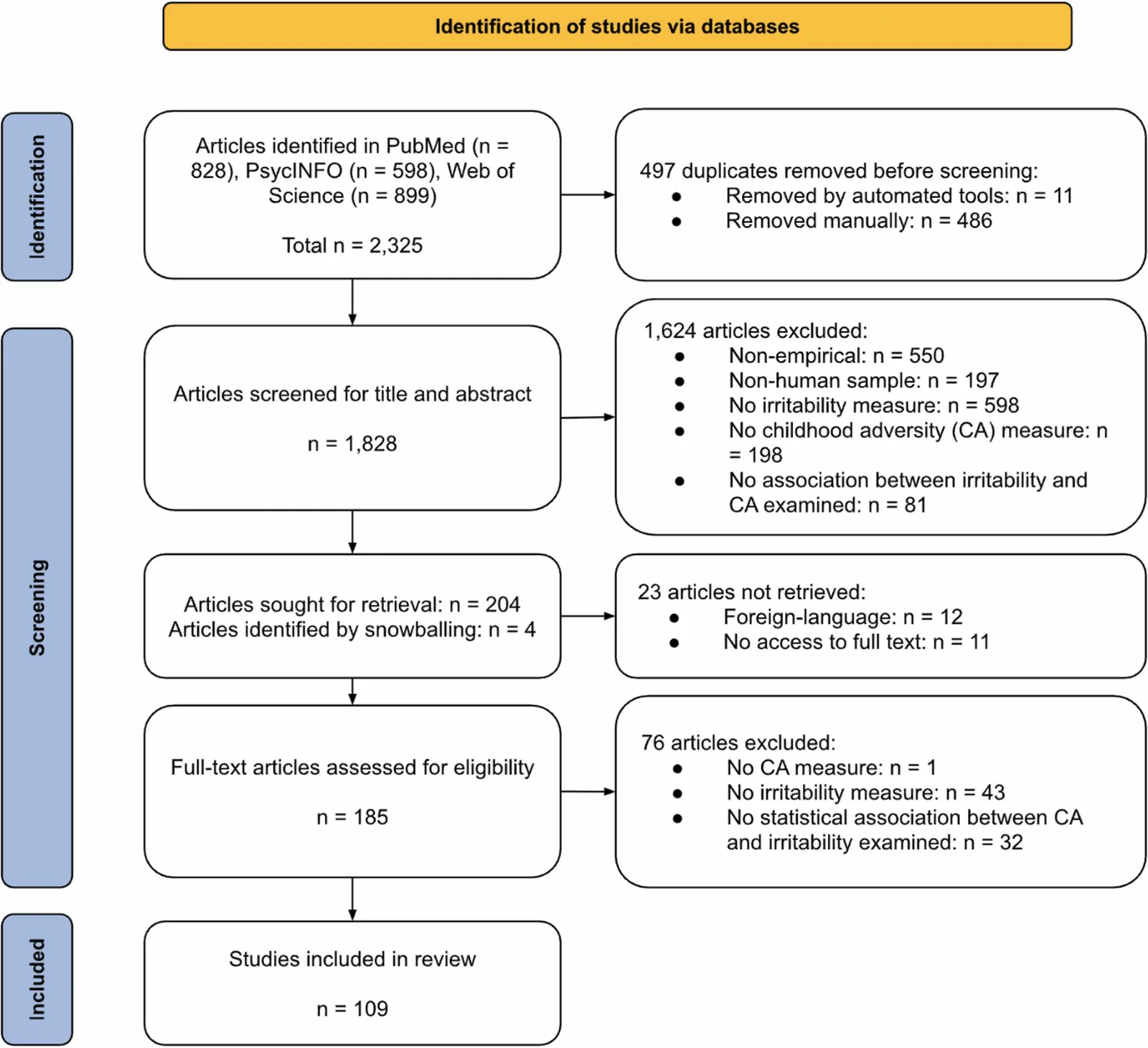
PRISMA flow diagram of article selection process.

### 2.4 Study risk of bias and quality assessment

Risk of bias and methodological quality of included studies was rated using a modified version of the National Institute of Health’s Quality Assessment Tool for Observational Cohort and Cross-sectional Studies [39]. See supplemental methods for additional details. Quality assessment for each of included studies and the criteria used are presented in Supplemental Table S2 and Table S3.

### 2.5 Meta-analysis

Correlation coefficients, including Pearson’s and Spearman’s correlations, were extracted from each study and transformed into Fisher’s z. Pooled estimates were then back-transformed to correlation *r* for interpretability*.* Regression coefficients (e.g., *β*) were transformed into t-statistics, when standard error was reported or could be calculated, which was then transformed into partial correlation coefficients and subsequently into Fisher’s z. Statistics reflecting group comparisons, including *t* and *F* statistics, were extracted and transformed to Hedges’ *g* effect size values.

Two separate random-effects meta-analyses, which accounted for effects nested within studies, were conducted to estimate the overall *r* and Hedges’ *g* for association and group comparison studies, respectively. Hedges’ *g* and *r* effect sizes of 0.2 were considered small, 0.5 were considered medium, and 0.8 were considered large [40]. Analyses were conducted in R using the metafor package [41]. We opted to conduct separate analyses for association and group comparison studies due to their inherent methodological heterogeneity. Age, gender, type of CA, and anger versus irritability measure were included as covariates.

## 3 Results

A total of 109 articles were included in the present review. See Fig. 1 for study selection procedures. Study characteristics (e.g., sample demographics, recruitment source) are presented in Table S4. Primary findings are presented in Tables S5 (Aims 1 and 2) and Table 1 (Aim 3).

**Table 1:** Design and Findings of Included Mediation Studies (Aim 3).

**Study**	**Design**	**Sample**	**Childhood Adversity**	**Irritability**	**Mediating Variable**	**Main Findings**
Berthelot et al. [42]	Cross-sectional	114 adults consulting for sexual dysfunction or dissatisfaction	Childhood abuse and neglect on ETI-SR and CCMS	Self-reported anger on STAXI	Self-reported PTSD symptoms	**Approach: Path Analysis** PTSD mediated the relationship between childhood maltreatment and trait anger (*z* = 1.88, *p* = .06, marginally significant).
Fuchshuber et al. [43]	Cross-sectional	616 adults in Austria	Self-reported childhood maltreatment on CTQ	Self-reported ANPS-Anger	Self-reported adult attachment style on the AAS Self-reported personality structure on the IPO	**Approach: Path Analysis** Structural deficits in personality structure and comfort with depending on others (from adult attachment scale) mediated the relationship between childhood maltreatment and anger (*β* = .20; *p* < .01).
Gardner et al. [44]	Cross-sectional	88 adult violent offenders (IPV and non-IPV) mandated for mental health treatment	Self-reported childhood maltreatment on CTQ	Self-reported trait anger on STAXI-2	Self-reported emotion regulation on the DERS	**Approach: Mediation** Non-intimate partner violence offenders: Difficulties in emotion regulation mediated the relationship between childhood maltreatment (physical abuse and neglect) and trait anger (physical abuse: point estimate = .45, *p* < .05; neglect: point estimate = .42, *p* < .05). Intimate partner violence offenders: Difficulties in emotion regulation mediated the relationship between childhood maltreatment (emotional abuse, emotional neglect, and physical abuse) and trait anger (emotional abuse: point estimate = .16, *p* < .05; emotional neglect: point estimate = .12, *p* < .05; physical abuse: point estimate = .10, *p* < .05).
Keene & Epps [45]	Cross-sectional	400 adult undergraduate students	Self-reported childhood abuse on the EPAB	Self-reported trait anger on STAXI	Self-reported narcissism on the PNI Self-reported shame on the ESS	**Approach: Mediation** Narcissistic vulnerability (point estimate = 0.29, *p* < .001)and shame-proneness point estimate = 0.22, *p* < .001) indirectly mediated the relationship between childhood physical abuse and trait anger.
Zhou & Zhen [46]	Cross-sectional	1689 adolescents	Self-reported emotional and physical abuse on CTQ	Self-reported trait anger on the Chinese trait anger scale	Self-reported cognitive reappraisal and expressive suppression on the Chinese Revised ERQ	**Approach: Structural Equation Modeling** There was an indirect positive effect of emotional abuse on anger through the mediating role of expressive suppression (*β*= .05; *p* < .05).

**Note.** Statistics, including nonsignificant findings, are included alongside descriptive results when reported. *β* refers to standardized regression coefficients, as reported.

Abbreviations in the order of appearance: ETI-SR=Early Trauma Inventory-Self Report; CCMS=Comprehensive Childhood Maltreatment Scale for Adults; STAXI=State-Trait Anger Expression Inventory; STAXI-2 =State-Trait Anger Expression Inventory-2; PTSD=posttraumatic stress disorder; CTQ=Childhood Trauma Questionnaire; ANPS=Affective Neuroscience Personality Scale; AAS=Adult Attachment Scale; IPO=Inventory of Personality Organization; DERS=Difficulties in Emotion Regulation Scale; EPAB=Emotional and Physical Abuse Questionnaire; PNI=Pathological Narcissism Inventory; ESS=Experience of Shame Scale; ERQ=Emotion Regulation Questionnaire.

### 3.1 Study characteristics

#### 3.1.1 Study quality

Risk of bias and quality assessment for each study are presented in Supplemental Table S2. The majority of studies received the highest quality rating (k = 72), while the remaining studies received a rating of ‘fair’ (k = 36) and only one study received a rating of poor. The most common reasons for lower quality ratings were failing to provide a sample size justification or power analysis and insufficient reporting of details regarding recruitment (e.g., recruitment from similar populations, uniform application of inclusion and exclusion criteria).

#### 3.1.2 Sample characteristics

Sample characteristics are summarized for each study in Table S4.

#### 3.1.3 Assessment of childhood adversity

CA was assessed in different ways, primarily with standardized questionnaires (k = 80), review of official records (k = 7), interviews (k = 3), and a mixed-assessment approach (k = 6; e.g., review of official records and caregiver report, standardized questionnaires and construct-specific items). The majority of studies with adults used self-reported CA (k = 51), while others used medical (k = 2) or child protective services records (k = 1), or clinician report (k = 2). As such, most adult reports of CA were retrospective. Child studies had more variability in report, including self-report (k = 38), a combination of self- and caregiver-report (k = 1), official records (k = 4), caregiver-report (k = 4), and a combination of official records and self- or caregiver-report (k = 6). See supplemental results for detailed description of CA measures.

#### 3.1.4 Assessment of irritability or trait anger

Studies primarily utilized standardized questionnaires (k = 99), construct specific items (k = 9), or a latent factor of irritability (k = 1). All adult studies used self-report measures (k = 54) but two (i.e., combined self-caregiver, nurse-reported). Six studies with children relied on caregiver reports, one used combined self- and caregiver-report, and all others relied on self-report (k = 53).

Importantly, only a few studies examined irritability specifically (k = 18) and the majority of the studies (k = 89) measured anger (see supplemental results for detailed description of irritability and anger measures). However, a closer examination of the content of the irritability and anger measures revealed substantial overlap and common themes. For example, the trait anger subscale of the State-Trait Anger Expression Inventory (STAXI) [47] emphasizes a dispositional tendency to experience and/or display anger, which is consistent with the conceptualization of irritability as a general proneness to anger. Items of the Affective Reactivity Index [48] - a gold-standard measure of irritability, also echo those used in anger measures (e.g., low threshold for anger, frequently and easily become angry). The overlapping items of irritability and anger measures reflect their operational similarities in the current literature [30].

#### 3.1.5 Analytic approaches and study design

The studies retained in Aims 1 and 2 utilized correlation and regression analyses, often examining the associations between CA and irritability or trait anger in the context of other covariates (e.g., age, sex assigned at birth, maternal education, socioeconomic status). Some studies also used group-based comparisons, examining differences in irritability or trait anger between individuals with or without adversity, or across levels of adversity severity. Aim 3 studies primarily explored mechanisms linking CA and irritability via mediation analysis, path analysis, or structural equation modeling. The majority of the included studies were cross-sectional, with only 13 studies utilizing longitudinal designs, two of which took a prospective approach.

### 3.2 Does extant empirical evidence support a significant association between CA and irritability (Aim 1)?

Qualitatively, 91% (48 out of 53) of studies conducted in children and adolescents, as well as 93% (52 out of 56) of studies in adults, identified a significant, positive association between CA and irritability or anger. In the meta-analysis, 208 effect estimates were extracted from 85 studies, encompassing 82 unique samples of 111,084 participants. The multilevel random-effects model for association-based studies (63 studies/60 samples contributing to 164 effect estimates) was significant and revealed a positive, small association between CA and irritability or trait anger (pooled association *r* = 0.22, 95% CI [0.19–0.24], *p* < .0001). Similarly, the random-effects model for group comparison studies (22 studies/samples contributing to 44 effect estimates) revealed a positive, medium association between CA and irritability or trait anger (Hedges’ *g* = 0.53, 95% CI [0.35–0.71], *p* < .0001). Substantial heterogeneity across studies for both models was observed (total *I*^2^ = 89.4% and 99% for association and group comparison studies, respectively). Egger’s test indicated no evidence of publication or other selection bias (*p* = 0.70 and 0.21 for association and group comparison studies, respectively). See Fig. 2, Fig. 3 (Child Studies) and Fig. 4, Fig. 5 (Adult Studies) for forest plots of these findings.

**Fig. 2:**
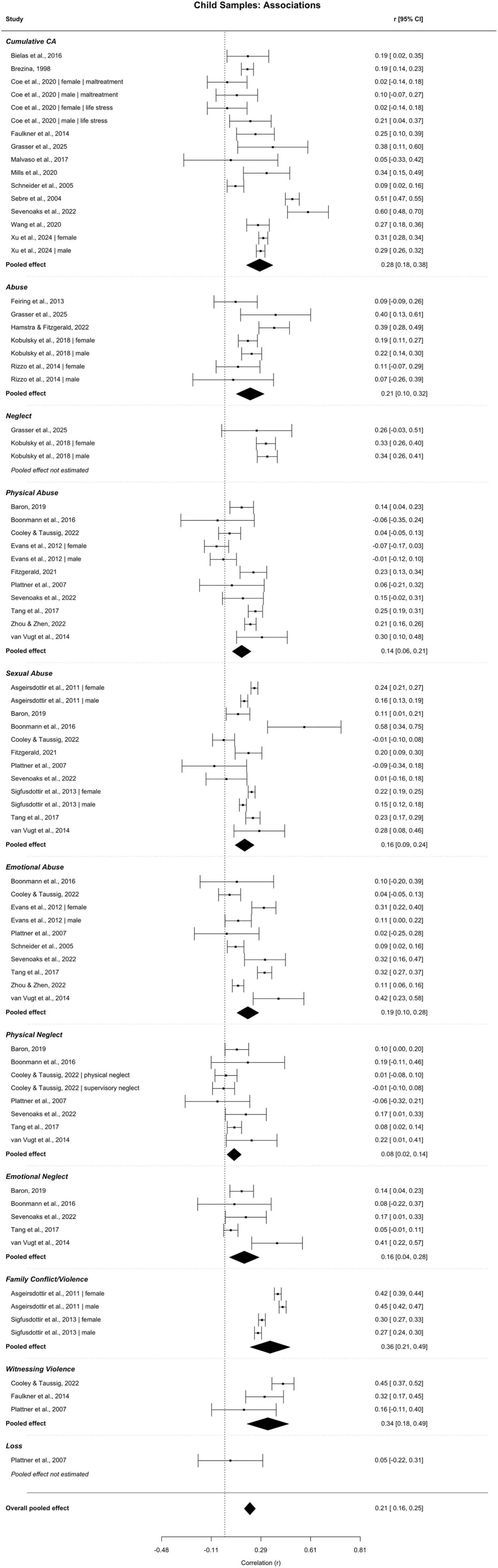
Forest plot demonstrating a small effect across child association studies for the relationship between CA and irritability or trait anger.

**Fig. 3:**
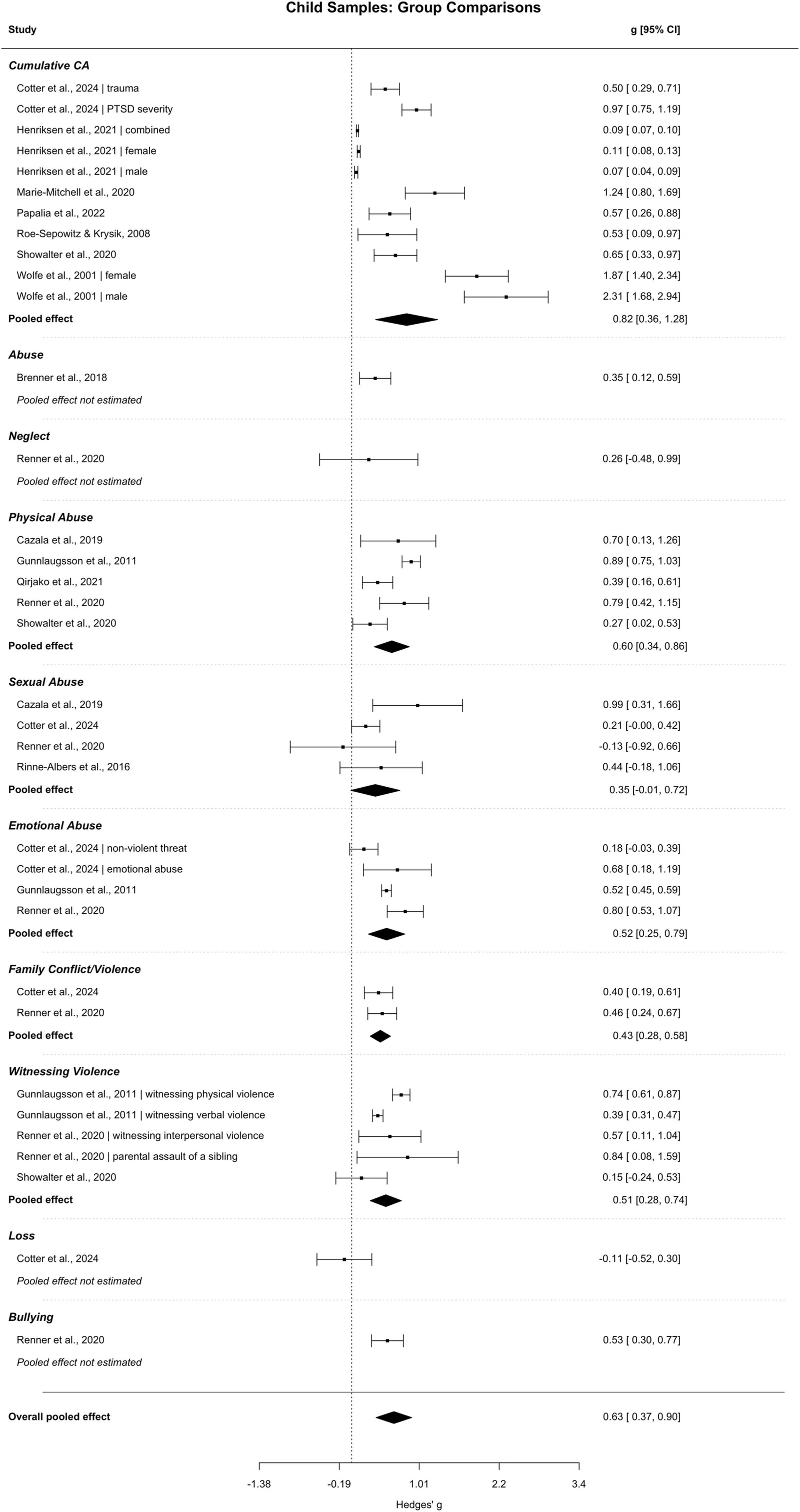
Forest plot demonstrating a medium effect across child group comparison studies for the relationship between CA and irritability or trait anger.

**Fig. 4:**
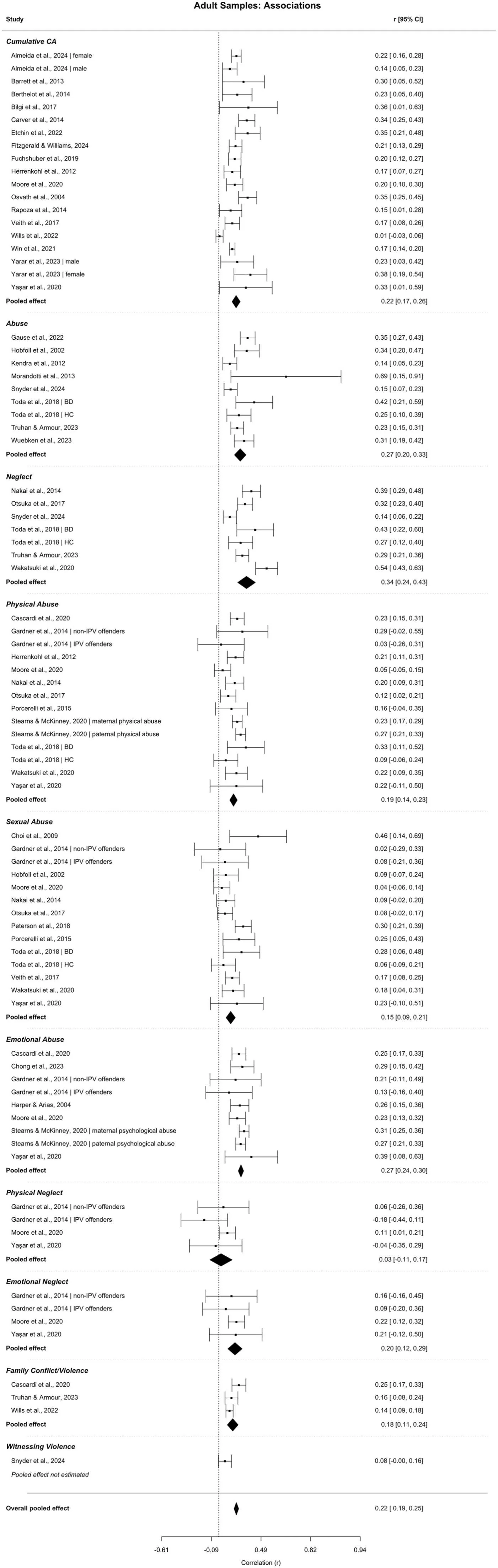
Forest plot demonstrating a small effect across adult association studies for the relationship between CA and irritability or trait anger.

**Fig. 5:**
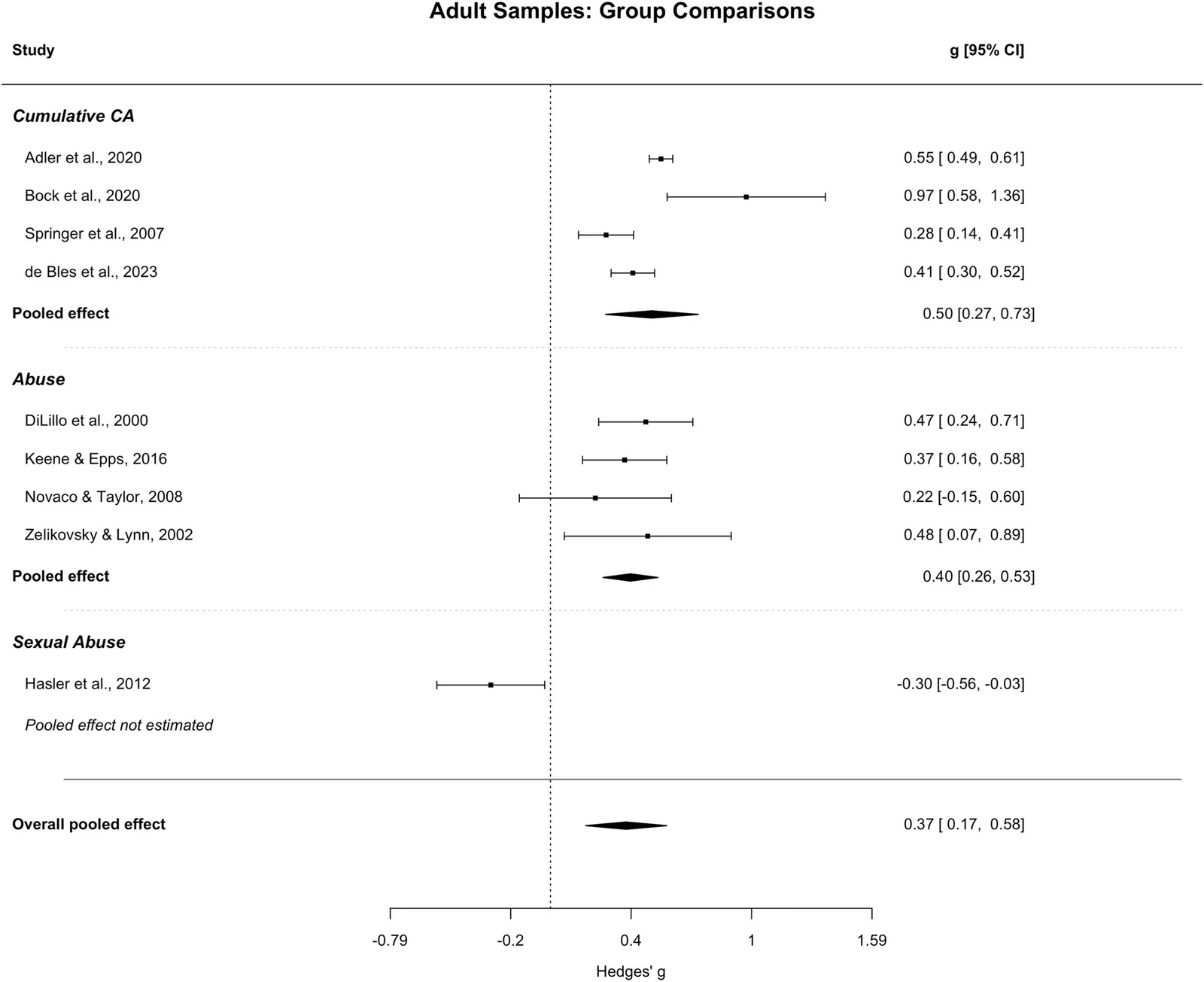
Forest plot demonstrating a small effect across adult group comparison studies for the relationship between CA and irritability or trait anger.

In addition, we examined the moderating effects of CA type, the irritability versus anger as the outcome measure, and sample characteristics (e.g., gender), as detailed below.

#### 3.2.1 Does the association differ by types of adversity?

Different approaches of measuring CA were utilized, including cumulative (e.g., total ACEs) and dimensional (e.g., abuse versus neglect as proxies of the threat versus deprivation dimensions) approaches, reflecting much of the existing literature (see [49] for a review). Additionally, some studies examined specific types of CA, such as different types of abuse and neglect and exposure to violence (e.g., family/interpersonal violence, community violence).

Random-effects meta-analysis revealed significant moderating effects of CA type within association studies. Of note, CA type was dummy coded and cumulative CA served as the reference. Compared to cumulative CA, overall neglect was associated with a significantly larger effect (*b* = 0.13, *p* = 0.01, 95% CI [0.04 – 0.22]), whereas physical neglect (*b* = −0.17, *p* = 0.0003, 95% CI [-0.25 - −0.1]), physical abuse (*b* = −0.08, *p* = 0.04, 95% CI [-0.16 - −0.01]), and sexual abuse (*b* = −0.1, *p* = 0.01, 95% CI [-0.18 - −0.02]) were associated with significantly smaller effects. Among group comparison studies, compared to cumulative CA, overall abuse (*b* = −0.35, *p* = 0.04, 95% CI [-0.68 - −0.03]), sexual abuse (*b* = −0.56, *p* = 0.04, 95% CI [-1.09 - −0.03]), and witnessing violence (*b* = −0.38, *p* = 0.03, 95% CI [-0.70 - −0.06]) were associated with smaller effects.

#### 3.2.2 Does the association differ by irritability versus anger measures?

Random-effects meta-analysis examined whether the use of irritability versus anger as the outcome measure was linked to different effect sizes. Here, outcome measure type was dummy coded and anger measure served as the reference. Among association studies, those that used irritability as an outcome measure demonstrated a significantly larger effect (*b* = 0.07, *p* = 0.02, 95% CI [0.01 – 0.12]), compared with anger as an outcome measure. In contrast, among group comparison studies, using anger versus irritability as an outcome measure did not emerge as a significant moderator (*p* > .05).

Notably, irritability and anger have been historically used interchangeably in many empirical studies, with overlapping content. Thus, the significant moderating effect observed in association studies should be interpreted with caution, particularly given that most studies in our review examined trait anger.

#### 3.2.3 Does the association differ by participant sex and/or gender?

Among 53 child studies, 11 examined the associations between CA and irritability or anger by child sex and/or gender. Seven studies reported no significant differences [20,[50], [51], [52], [53], [54], [55]], while four studies identified different patterns by child sex and types of CA. Meta-analytic results suggest that across all studies, gender was not a significant moderator of the association between CA and anger or irritability (*p*s > .05 for both association and group comparison studies), though sex and/or gender comparisons within individual studies suggest differences may be present in some samples. See supplemental results for detailed description of studies examining sex and/or gender.

#### 3.2.4 Does the association differ by clinical presentations?

Given the limited number of studies reporting differences between clinical and non-clinical samples, clinical presentation was not included as a moderator in the meta-analyses. See supplemental results for detailed, qualitative description of findings based on clinical presentation.

### 3.3 Does the association between CA and irritability persist from childhood through adulthood (i.e., across lifespan) (Aim 2)?

An important and clinically relevant question is whether the associations between CA and irritability or anger last through childhood to adulthood. Meta-analysis showed that age was not a significant moderator of the relationship between CA and irritability or trait anger across studies (*p*s > .05), suggesting that the strength of association likely remained consistent across ages. However, a proper examination of this research question requires a longitudinal, and more ideally, prospective study design, where measures of *current* adversity are collected and participants are followed over time. Overall, nine child studies and four adult studies utilized a longitudinal design, whereas only two (one child and one adult) took a prospective approach, using official records to determine adversity exposure (in contrast to retrospective reports of adversity). All longitudinal studies but one identified a significant, positive association between CA and later irritability or anger, with one prospective child study reporting null findings [56] between abuse severity and anger.

### 3.4 What are the potential mechanisms by which CA and irritability may be associated with each other (i.e., mediators) (Aim 3)?

Five studies explored potential variables mediating the relationship between CA and anger, four of which included adult participants [[42], [43], [44], [45]] and one included adolescent participants [46]. All potential variables examined were self-report measures exploring psychological mediators between childhood adversity and anger. There were no studies specifically examining irritability as an outcome measure. Hypothesized mediators were common transdiagnostic mechanisms often studied in both internalizing and externalizing disorders, ranging from cognitive and emotional processing (i.e., difficulty in emotion regulation, expressive suppression), personality traits (i.e., narcissism, shame-proneness, deficits in personality organization) and attachment (i.e., insecure adult attachment). Temporal precedence was not established in any of these cross-sectional analyses [57]. As such, these studies examined statistical mediation but were not designed to test causal mediation. All but one study reported significant findings (with small effects), such that each variable above significantly mediated the relationship between childhood adversity and anger.

Two studies suggest that certain personality traits may be one pathway between CA and anger. Keene & Epps [45] found that shame-proneness and narcissistic vulnerability (pathological narcissism characterized by hypersensitivity and defensiveness) mediated the relationship between childhood physical abuse and trait anger in a sample of 400 undergraduates. Another study by Fuchshuber et al. [43] identified deficits in personality organization (e.g., poor reality perception, unstable sense of self) and insecure adult attachment as mediators between overall maltreatment and anger.

Emotion regulation difficulties, a mechanism often linked to functionally impairing anger or irritability, was also found to mediate the relationship between CA and anger in two studies. In a sample of 88 adult offenders mandated for mental health treatment, difficulty in emotion regulation mediated the relationship between childhood physical abuse and neglect and trait anger [44]. Zhou & Zhen [46] observed a similar relationship in a large sample of adolescents, such that expression suppression, an emotion regulation strategy associated with psychopathology when habitually used, mediated the effect of emotional abuse on trait anger.

Finally, one study did not report a significant mediation between CA and trait anger [42]. In a cross-sectional study of adults consulting for sexual dysfunction, self-reported PTSD symptoms did not significantly mediate the association between childhood abuse and neglect and trait anger, though this finding was marginally significant (*p* = .06). Null findings may be partially explained by the use of a dichotomous variable for CA, which may have resulted in underestimated between-subject variability.

Together, these studies suggest that childhood adversity may impact key developmental processes in emotional, cognitive, and personality domains, which in turn may increase trait anger later in life. However, all studies were cross-sectional and thus established statistical, but not causal, mediation.

## 4 Discussion

The current review systematically synthesized and meta-analyzed extant empirical literature examining associations between CA and irritability, as well as trait anger, a closely related construct that is often used interchangeably with irritability, across the lifespan. Studies exploring the potential mechanisms underlying such associations were also reviewed. While prior work has primarily linked early adverse experiences to the development of specific psychiatric disorders, our findings show a robust link between CA and a dimensional, transdiagnostic trait that is associated with a variety of symptom profiles, which may offer insights into both understanding the etiology of irritability and developing novel treatment approaches to adversity-related psychopathology.

Several nuances about these associations are worth noting. First, studies retained in this review took different approaches to conceptualizing and operationalizing CA, and overall, they identified a positive, small-to-medium effect size relationship between CA and irritability or trait anger. Cumulative CA, the most frequently examined among these, showed a stronger association, compared with single-domain CA subtypes (e.g., physical abuse, sexual abuse, physical neglect, witnessing violence). Overall neglect was noted to have a stronger association with irritability or trait anger than cumulative CA, for association studies only. This interpretation should be considered with caution, however, due to uneven empirical work on different types of CA, as evidenced by far fewer studies examining CA subtypes in the current review, relative to cumulative CA (e.g., total maltreatment, trauma count and severity), the high co-occurrence rate of different CA types (e.g., abuse and neglect) [58], and the inherent difficulties teasing apart their specific effects [59].

Additionally, we included studies that assessed irritability and/or anger as an affective trait, and excluded those that only looked at its behavioral manifestations (e.g., aggression) or temporary reactions (e.g., state anger). Not surprisingly, given the relatively nascent stage of irritability research, most studies (83%) in our review measured trait anger, albeit with substantial content overlap with irritability measures (e.g., proneness to anger, anger frequency and intensity). Further, as definitions of anger and irritability continue to evolve and be refined [30], current frameworks suggest that elevated irritability may predispose individuals to experiencing and expressing negative emotions (e.g., anger, frustration, aggression) and thus encompass expressed trait anger. Within this context, our finding that there was a stronger association between CA and irritability, compared to trait anger, should be interpreted with caution. Empirical studies that examine theoretical and behavioral similarities, as well as differences, between irritability and trait anger, are needed to enhance the precision of assessment.

A handful of studies also examined whether the associations between CA and irritability or trait anger differed by participant characteristics, such as sex and/or gender, and clinical presentations. Sex or gender did not emerge as a significant moderator in the meta-analyses, suggesting comparable relationships between CA and irritability or trait anger by sex and/or gender. Regarding clinical presentations, a few studies showed that the associations were unique to participants in certain clinical settings relative to healthy controls (e.g., being treated for BPD or schizophrenia) [18,60,61]. Though future investigation with larger sample sizes is needed, these nuances echo the need for improved specificity of clinical research and may provide critical insights into identifying subpopulations at higher risks to improve treatment development and allocation.

Notably, burgeoning empirical research on irritability in the past two decades has predominantly focused on pediatric populations [62]. However, the etiology of irritability in youth remains unclear, with recent work only just beginning to examine early life experiences as a risk factor for elevated irritability. On the other hand, among adults, despite the heterogeneity of trauma-related symptomatology, irritability and its closely related constructs such as trait anger are commonly recognized psychological responses after trauma exposure [20,21] and is one of the hallmark symptoms of PTSD. In this light, the current review included studies conducted across the lifespan to synthesize these two realms of literature and paints a preliminary picture of the comparable associations between CA and irritability (or trait anger) in youth and adults. Further, by looking at both child and adult literature, we examined whether the effects of CA persist from childhood to adulthood and age was not a significant moderator of the association between CA and irritability or trait anger. Most studies retained in this review, however, were cross-sectional, undermining our ability to make inferences about the continuity versus discontinuity of CA-related irritability or trait anger. Two studies utilized a prospective design (where data on CA was collected using official records at the baseline), with one reporting null findings between childhood abuse severity and trait anger 35 years later [56] and the other reporting a significant positive association between childhood maltreatment, as well as abusive disciplining, with anger proneness 6 years later [63]. More prospective studies are thus needed to delineate the downstream effects of CA and avoid limitations of retrospective reporting, which may also help elucidate etiological mechanisms of irritability or trait anger.

Lastly, to better understand *how* CA is related to elevated irritability or trait anger, we reviewed studies exploring potential mediators underlying this association. Only five studies conducted mediation analyses, and all but one identified significant psychological and cognitive mediators spanning emotion regulation, attachment, and personality traits. Results suggest that CA may disrupt key developmental processes (e.g., emotion regulation) or may interact with existing traits (e.g., personality traits, attachment) across cognitive and emotional domains that subsequently increase susceptibility to impairing levels of anger. Significant mediation effects observed across diverse samples (adolescents, adults, offenders, treatment-seeking) and varying types of CA (overall maltreatment, abuse, neglect) suggest these pathways may be relatively robust. However, it is critical to note that no mediational study included a longitudinal design, and studies were primarily conducted with adults. Reports of adversity were retrospective, and these cross-sectional designs preclude our ability to truly establish temporal precedence and determine whether elevated anger began following experiences of adversity. Moreover, none of the studies established whether the significant statistical mediators were temporal mediators, as such, our ability to label the identified mediators as causal and leading to elevated anger is limited. While there are financial and ethical constraints to longitudinal prospective studies on CA, these designs are needed to conduct temporal mediation studies that identify causal pathways [57].

Nevertheless, emotion regulation has been consistently identified as a developmental process influenced by childhood adversity and associated with later internalizing and externalizing psychopathology. Prior work suggests that early adversity disrupts typical emotional development, leading to difficulty regulating emotional responses and disengaging from emotional stimuli, and an increase in the use of maladaptive emotion regulation strategies (e.g., rumination) [64]. Thus, CA may shape developmental pathways related to emotion regulation, leading to heightened anger or irritability later in life. Indeed, emotion dysregulation has been identified as a predictor of externalizing symptoms in adolescents [65], and is a core symptom of disorders characterized by chronic irritability (i.e., DMDD). Findings suggest that the developmental impacts of CA may also interact with existing traits or vulnerabilities to predict anger or irritability. For example, narcissism, shame-proneness, and insecure attachment explained the relationship between CA and anger, though it is challenging to parse apart whether these more static personality traits were shaped by early adverse experiences or whether a reciprocal relationship between CA and personality traits exists.

While our findings suggest CA may impact cognitive and emotional processes, we did not identify any studies exploring biological mechanisms mediating the relationship between CA and irritability or trait anger. Prior studies have linked CA to developmental changes in brain structure and function [66], physiological responses [67], and hormonal dysregulation [68]. However, these mechanisms have not yet been studied in relation to irritability or trait anger. Future work should examine whether biological pathways mediate associations between CA and irritability-related outcomes.

### 4.1 Strengths and limitations

The retained studies of the current review have several strengths. First, there was a relatively equal representation of studies conducted with children (including adolescents) and adults, which allowed us to compare findings in these two populations. Second, the majority of studies had moderate to large sample sizes (e.g., 68 studies with 100–1000 participants, 26 studies with over 1000 participants) and studies were conducted in samples of relatively diverse characteristics, in terms of country/region, recruitment source (e.g., general community, juvenile detention centers, and specialty clinics), and racial and ethnic identities. Studies included both clinical and community samples, ensuring associations observed are not simply attributable to symptoms of a particular disorder. However, findings should be considered in the context of the following limitations of the extant literature. First, as described above, there was a lack of studies that examined irritability-specific associates of CA due to a lack of clarity and consistency in how irritability has been defined and operationalized [30]. Second, there were few longitudinal studies, particularly prospective ones, that traced the impact of CA across the lifespan, limiting the examination of temporal relationships and the unique effects of CA. Lastly, most studies relied on retrospective assessment of CA, with few studies incorporating prospective assessments (e.g., official record review), which may have introduced reporting bias [69,70].

The current review also has strengths and limitations. It contributes to the existing literature as the first review synthesizing and meta-analyzing empirical studies on the associations between CA and irritability or trait anger. By incorporating studies conducted in both children and adults, it provides a more developmentally-informed lifespan perspective to conceptualize both CA and irritability/trait anger. Potential mechanisms linking CA and irritability or trait anger were also explored, which offers preliminary insights into improving treatment targets (e.g., emotion regulation) and development. Several limitations of our protocol for this review warrant discussion. First, we limited our definition of CA to adverse experiences commonly assessed in the existing literature (e.g., childhood maltreatment, witnessing domestic violence) at the individual level; however, emerging research suggests structural CA (e.g., poverty, discrimination, exposure to pollutants) impacts mental health outcomes. Future work should consider expanding definitions of CA to explore how structural adversity trickles down to affect individual outcomes. Second, we limited the scope to only include studies examining direct associations between CA and irritability or studies exploring the mechanisms linking CA to irritability. We did not include studies exploring how this association may lead to later psychopathology. Thus, we are not fully able to interpret whether the irritability or anger associated with adverse experiences reflect symptoms of a particular disorder (e.g., PTSD), or if experiencing adversity increases irritability, which in turn increases risk for developing a psychological disorder. Third, while we chose to specifically examine associations between CA and irritability or trait anger, some part of this relationship is likely explained by higher order factors such as negative affectivity/neuroticism. A recent meta-analysis identified a similar positive association between CA and adult neuroticism [33], highlighting the possibility that associations observed in the current review may partly reflect this broader trait. Finally, as with all systematic reviews, the current review may be limited by publication bias, resulting in an underrepresentation of studies observing null associations and only including manuscripts in English.

### 4.2 Conclusions

This comprehensive systematic review and meta-analysis supports a robust and positive relationship between CA and irritability/trait anger in both children and adults. Findings from this review should be considered in the context of prevention and early diagnosis efforts, as elevated trait anger and irritability are associated with psychopathology and notable functional impairment later in life. Early screening efforts to identify both CA exposure and elevated levels of these traits may improve precision in intervention approaches, development, and allocation (e.g., transdiagnostic treatment targets for trauma-related symptomatology, trauma-informed assessment of irritability). Further, our review identified potential mediators (e.g., emotion regulation, attachment) explaining this relationship, providing preliminary insight into the mechanisms by which CA and irritability/trait anger may be linked, though prospective or experimental designs are needed to validate these cross-sectional mediations.

The current review also offers several directions for future research, including the need for 1) prospective, longitudinal studies to better trace the developmental pathways of how CA leads to irritability; 2) improved consistency and precision in how irritability is defined and measured in children and adults; 3) additional work considering how early adverse experiences shape biological processes and lead to impairing irritability or trait anger; and 4) a closer examination of how individual and contextual differences may impact (e.g., amplify or protect against) the association between CA and irritability, to improve intervention precision. These advances may ultimately be used to inform early prevention and intervention efforts and reduce the burden of CA-related elevated irritability and anger and other related psychopathology.

## CRediT authorship contribution statement

**Isabella G. Spaulding:** Writing – Original draft preparation, Visualization, Methodology, Investigation, Data curation, Conceptualization. **Ruiyu Yang:** Writing – Original draft preparation, Visualization, Methodology, Investigation, Data curation, Conceptualization. **Grace Cotter:** Writing – review & editing, Validation. **Alexa Duran:** Writing – review & editing, Validation. **Grace Kathman:** Writing – review & editing, Validation. **Murray B. Stein:** Writing – review & editing, Supervision. **Charles T. Taylor:** Writing – review & editing, Supervision, Funding acquisition. **Jillian Lee Wiggins:** Writing – review & editing, Supervision, Funding acquisition.

## Funding

This work was supported by the National Institute of Mental Health (NIMH) grant (R01MH122487; R01MH121385) to JLW, the National Institute of Mental Health (NIMH) grant (R61MH129380) to CTT, and the National Science Foundation (NSF) Graduate Research Fellowship Program to IGS.

## Declaration of Competing Interest

The authors declare the following financial interests/personal relationships which may be considered as potential competing interests: Isabella Spaulding reports financial support was provided by National Science Foundation. Jillian Lee Wiggins reports financial support was provided by National Institute of Mental Health. Charles Taylor reports financial support was provided by National Institute of Mental Health. Charles Taylor, Murray Stein reports a relationship with Bionomics, atai Life Science, and Engrail Therapeutics that includes: consulting or advisory. Charles Taylor, Murry Stein reports a relationship with UptoDate Inc that includes: employment. Murray Stein reports a relationship with Ananda Scientific, BigHealth, Biogen, Boehringer Ingelheim Ltd, EmpowerPharm, Jazz Pharmaceuticals, Karuna Therapeutics, Lundbeck, Lykos Therapeutics, Newleos Therapeutics, Orion Pharma, Otsuka US, PureTech Health, Roche Genentech, SAGE Therapeutics Inc, Seaport Therapeutics, Sensorium Therapeutics, and Transcend Therapeuticsthat includes: consulting or advisory. Murray Stein reports a relationship with EpiVario, Newleos Therapeutics, Inc., and Oxeia Biopharmaceuticals that includes: equity or stocks. Murray Stein reports a relationship with Biological Psychiatry that includes: board membership and employment. Murray Stein reports a relationship with Brain & Behavior Research Foundation and Anxiety and Depression Association of America that includes: board membership. Given his role as editor, MBS had no involvement in the peer review of this article and had no access to information regarding its peer review. Full responsibility for the editorial process for this article was delegated to another journal editor. If there are other authors, they declare that they have no known competing financial interests or personal relationships that could have appeared to influence the work reported in this paper.

## Footnotes

Appendix A Supplementary data associated with this article can be found in the online version at doi:10.1016/j.xjmad.2026.100204.
