# Supplementary material for: Associations and mechanisms between childhood adversity and irritability or trait anger across the lifespan: A systematic review and meta-analysis
Source: J Mood Anxiety Disord. 2026 Sep 24;16:100204. doi: 10.1016/j.xjmad.2026.100204 (PMC13653991; doi:10.1016/j.xjmad.2026.100204)
Supplement: Supplementary file 1 [file mmc1.docx]

**SUPPLEMENTARY INFORMATION**

# Associations and Mechanisms between Childhood Adversity and Irritability or Trait Anger Across the Lifespan: A Systematic Review and Meta-Analysis

**Methods**

***Study Risk of Bias and Quality Assessment***

We included the 8 applicable items from the rating scale (yes/no items), which assess the quality and internal validity of studies in multiple domains, including sample size justification, use of validated measures, and consideration of confounding variables. Studies were rated as ‘poor’, ‘fair’, or ‘good’, reflecting the number of ‘yes’ responses out of the total number of applicable questions for each study. Studies with 7+ were rated ‘good’, 5-6 were rated ‘fair, and 4 or less were rated ‘poor’.

**Results**

***Sample Characteristics***

Publication year ranged from 1998 to 2025. Studies were primarily cross-sectional (k=96), while 13 studies used a longitudinal design. The majority were conducted in the United States (k=58), followed by Canada (k=6) and the United Kingdom (k=5), representing primarily Western, educated, industrialized, rich, and democratic (WEIRD) populations [[1]](https://www.zotero.org/google-docs/?rT1d6f). Only 16 studies were conducted in countries outside of Europe, Australia, or North America, though some studies specifically included samples of racial and ethnic minorities (e.g., [[2–4]](https://www.zotero.org/google-docs/?SbopWR)). The majority of studies included samples between 100 and 1000 participants (k=68), though a notable number of studies included sample sizes with over 1,000 participants (k=26) and 15 studies included fewer than 100 participants. Participants were primarily recruited from community and clinical samples, as well as juvenile detention centers.

Over half of the studies consisted of only adult participants (k=56), while 40 studies included child or adolescent samples and 13 studies included both adolescents and young adults. Mean age ranged from 4.2 to 58 years old. Over half of the studies reported race and/or ethnicity of participants (k=63), with relatively diverse racial and ethnic samples. Nineteen studies reported samples with over 75% Non-Hispanic White participants, while 16 studies included samples with 50-75% and 28 studies had samples with less than 50% White participants. Overall, studies were relatively well-balanced between male and female participants, though no studies included participants identifying as non-binary or gender nonconforming.

### ***Assessment of Childhood Adversity***

The most commonly used instrument measures of CA included the Childhood Trauma Questionnaire (k=34, including the short form) [[5]](https://www.zotero.org/google-docs/?IEQ5MV), the Conflict Tactics Scale (k=10) [[6]](https://www.zotero.org/google-docs/?PcrIbq), and the Adverse Childhood Experiences Scale (k=7) [[7]](https://www.zotero.org/google-docs/?0hIZwr). The types of CA assessed were primarily childhood maltreatment (i.e., physical, sexual, and emotional abuse; physical and emotional neglect) and witnessing domestic violence, with some studies also probing parental abusive disciplining, substance use, and marital problems, as well as adverse experiences outside of the immediate home environment (e.g., exposure to community violence, peer victimization) - often as complementary measures.

### ***Assessment of Irritability or Trait Anger***

Importantly, only a few studies examined irritability specifically (k=18), primarily using standardized measures such as the Caprara Irritability Scale [[8]](https://www.zotero.org/google-docs/?liqMQ6), the Irritability Questionnaire [[9]](https://www.zotero.org/google-docs/?A6MycK), the Affective Reactivity Index (ARI) [[10]](https://www.zotero.org/google-docs/?Cwi82n), and the Temperament Evaluation of Memphis, Pisa, Paris, and San Diego-Autoquestionnaire - Irritable subscale [[11]](https://www.zotero.org/google-docs/?8h7XZr). Two studies utilized angry-irritable subscales from established instruments [[4,12]](https://www.zotero.org/google-docs/?mpSiIL). The majority of the studies (k=89) measured anger, primarily using the anger subscale of the Trauma Symptom Checklist for Children (k=16) [[13]](https://www.zotero.org/google-docs/?dqopRR), the trait anger subscale of the State-Trait Anger Expression Inventory (STAXI) (k=19) [[14]](https://www.zotero.org/google-docs/?niHJjY), and the Dimensions of Anger Reactions (k=5) [[15]](https://www.zotero.org/google-docs/?IQusXb).

## **3.2 Does extant empirical evidence support a significant association between CA and irritability (Aim 1)?**

***Does the association differ by participant sex and/or gender?***

Among 53 child studies, 11 examined the associations between CA and irritability or anger by child sex and/or gender. Seven studies reported no significant differences [[2,16–21]](https://www.zotero.org/google-docs/?iyIpaf), with sample sizes ranging from 155 to 9,085. In contrast, four studies identified different patterns by child sex and types of CA. Specifically, Benedini & Fagan [[22]](https://www.zotero.org/google-docs/?lgJK29) reported that physical abuse was a significant predictor of anger in girls only, whereas sexual abuse was significant for both boys and girls. Cazala et al. [[23]](https://www.zotero.org/google-docs/?1QCVuJ) reported in a clinical (borderline personality disorder [BPD]) sample of children and adolescents that emotional abuse, emotional neglect, and physical neglect were significant predictors of increased irritability in males only, whereas sexual and physical abuse were significant for both males and females. Leban & Delacruz [[24]](https://www.zotero.org/google-docs/?J4XsX7) reported that household dysfunction ACEs were uniquely associated with anger for girls only. Lastly, Wolfe et al. [[25]](https://www.zotero.org/google-docs/?SUrB8T) found that moderate/severe maltreatment predicted increased anger for girls only. Sample sizes of these studies (*N*s=777-1,419, with *N* of one study=59) likely afforded sufficient statistical power to examine sex and/or gender differences, though power analysis was not directly reported in all studies.

Fewer studies (k=4) in adults examined the effects by sex and/or gender. Two of the four studies found no significant sex differences and identified psychological abuse [[26]](https://www.zotero.org/google-docs/?TkDVcc) and CA in general [[27]](https://www.zotero.org/google-docs/?2AOTWr) as significant correlates of anger. In contrast, Stearns & McKinney [[28]](https://www.zotero.org/google-docs/?o5jaIP) found that maternal and paternal psychological and physical abuse predicted anger for both males and females, whereas paternal psychological and physical maltreatment emerged as a significant predictor for females only. Another study conducted by Sudbrack et al. [[29]](https://www.zotero.org/google-docs/?IlP99e) observed significant associations between emotional abuse and anger for males only. Similarly, all studies reported fairly large sample sizes (*N*s=294-10,800).

Of note, five child and four adult studies had male-only samples; three child and six adult studies had female-only samples, with no notable differences between the findings of these studies (eight out of nine male-only studies and eight out of nine female-only studies identified a positive relationship between CA and irritability or anger) and those of other studies with mixed sex and/or gender.

***Does the association differ by clinical presentations?***

Nine child studies examined samples with a clinical diagnosis or in a clinical setting, including autism spectrum disorder (k=1), BPD (k=1), PTSD with child sexual abuse history (k=1), treatment-seeking (k=1), outpatient (k=1), residential care (k=2), inpatient (k=1), and pediatric medical clinics (k=1). Only one study directly compared a clinical subsample with healthy controls ([[30]](https://www.zotero.org/google-docs/?YgSngf); *N=*40) and found children with PTSD had significantly higher anger than healthy controls. All studies identified a significant positive association between the CA examined and irritability or anger, except for two that observed nonsignificant associations. These two studies examined the relationship between childhood maltreatment and anger in children recruited from pediatric medical clinics [[17]](https://www.zotero.org/google-docs/?yQjJLk) and in an inpatient sample [[20]](https://www.zotero.org/google-docs/?U68lgz).

Eleven adult studies examined clinical samples, including BPD (k=3), schizophrenia (k=1), anxiety/depression (k=1), alcohol use problems (k=1), outpatient (k=4), and inpatient (k=1). Four studies compared the clinical subsample with healthy controls, with three identifying effects unique to the clinical samples - one found that childhood maltreatment was a significant predictor of anger for schizophrenia patients only ([[31]](https://www.zotero.org/google-docs/?dAtVOq); *N=*72) and two found that childhood abuse ([[32]](https://www.zotero.org/google-docs/?j3rOYB); *N=*37) and child abuse, neglect, and punishment ([[33]](https://www.zotero.org/google-docs/?4mBOaX); *N=*245) were significant predictors of anger for BPD patients only. One did not report a significant difference between patients with anxiety/depression and healthy controls in the relationship between childhood trauma and anger ([[34]](https://www.zotero.org/google-docs/?QU52Tu); *N=*2,271). Of note, power analysis was not directly reported in all studies and some of these studies with smaller sample sizes may not be sufficiently powered to examine differential associations by clinical characteristics.

**Table S1. Boolean Search Terms**

| **Construct** | **Equivalent terms used in PubMed, APA PsycInfo, and Web of Science (title and abstract)** |
| --- | --- |
| Childhood Adversity | child* adversity, child* trauma, child* abuse, child* neglect, child* maltreatment, ‘early life stress,’ ‘early adversity,’ ‘adverse childhood experiences,’ ACE, ACEs  *MeSH terms (PubMed only): “Adverse Childhood Experiences”[Mesh], “Child Abuse”[Mesh], “Child Abuse, Sexual”[Mesh] |
| Irritability/Anger | irritability, irritable, frustration, anger, DMDD, disruptive mood dysregulation disorder, severe mood dysregulation, tantrum  *MeSH terms (PubMed only): “Irritable Mood”[Mesh], “Anger”[Mesh], “Frustration”[Mesh] |

**Table S2. Results of NIH Risk of Bias and Quality Assessment for Included Studies**

| **Study** | **Q1** | **Q2** | **Q3** | **Q4** | **Q5** | **Q6** | **Q7** | **Q8** | **Overall Rating** |
| --- | --- | --- | --- | --- | --- | --- | --- | --- | --- |
| Adler et al., 2020 | Yes | Yes | Yes | No | Yes | Yes | Yes | Yes | Good |
| Almeida et al., 2024 | Yes | Yes | NR | No | Yes | Yes | Yes | No | Fair |
| Asgeirsdottir et al., 2011 | Yes | Yes | NR | No | Yes | Yes | Yes | Yes | Good |
| Baron, 2019 | Yes | Yes | Yes | No | Yes | Yes | Yes | Yes | Good |
| Barrett et al., 2013 | Yes | Yes | Yes | No | Yes | Yes | Yes | Yes | Good |
| Beesley et al., 2010 | Yes | Yes | Yes | No | Yes | Yes | Yes | Yes | Good |
| Benedini et al., 2020 | Yes | Yes | NR | No | Yes | Yes | Yes | Yes | Good |
| Berthelot et al., 2014 | Yes | Yes | NR | No | Yes | Yes | Yes | No | Fair |
| Bielas et al., 2016 | Yes | Yes | Yes | No | Yes | Yes | Yes | Yes | Good |
| Bilgi et al., 2017 | Yes | Yes | No | No | Yes | Yes | Yes | Yes | Good |
| Bock et al., 2020 | Yes | Yes | Yes | No | Yes | Yes | Yes | Yes | Good |
| Boonmann et al., 2016 | Yes | Yes | No | No | Yes | Yes | Yes | No | Fair |
| Brenner et al., 2018 | Yes | Yes | Yes | No | Yes | Yes | Yes | Yes | Good |
| Brezina 1998 | Yes | Yes | NR | No | Yes | Yes | Yes | Yes | Fair |
| Briere et al., 2001 | Yes | No | NR | No | Yes | NR | Yes | Yes | Poor |
| Carver et al., 2014 | Yes | Yes | Yes | No | Yes | Yes | Yes | Yes | Good |
| Cascardi et al., 2020 | Yes | Yes | Yes | Yes | Yes | Yes | Yes | Yes | Good |
| Cazala et al., 2019 | Yes | Yes | Yes | No | Yes | Yes | Yes | Yes | Good |
| Cecil et al., 2014 | Yes | Yes | No | No | Yes | Yes | Yes | Yes | Good |
| Choi et al., 2009 | Yes | Yes | Yes | No | Yes | Yes | No | Yes | Fair |
| Chong et al., 2023 | Yes | Yes | NR | Yes | Yes | Yes | Yes | Yes | Fair |
| Coe et al., 2020 | Yes | Yes | No | No | Yes | Yes | Yes | Yes | Fair |
| Collin-Vézina et al., 2011 | Yes | Yes | Yes | No | Yes | Yes | Yes | No | Fair |
| Cooley et al., 2022 | Yes | Yes | Yes | No | Yes | Yes | Yes | Yes | Good |
| Cotter et al., 2024 | Yes | Yes | Yes | No | Yes | Yes | Yes | Yes | Good |
| de Bles et al., 2023 | Yes | Yes | Yes | No | Yes | Yes | Yes | Yes | Good |
| Derella et al., 2020 | Yes | Yes | Yes | No | Yes | Yes | Yes | Yes | Good |
| DiLillo et al., 2000 | Yes | Yes | Yes | No | Yes | Yes | Yes | Yes | Good |
| Etchin et al., 2022 | Yes | Yes | Yes | No | Yes | Yes | Yes | Yes | Good |
| Evans et al., 2012 | Yes | Yes | Yes | No | Yes | Yes | Yes | Yes | Good |
| Faulkner et al., 2014 | Yes | Yes | Yes | No | Yes | Yes | Yes | Yes | Good |
| Feiring et al., 2013 | Yes | Yes | Yes | No | Yes | Yes | Yes | No | Good |
| Fitzgerald, 2021 | Yes | Yes | Yes | No | Yes | Yes | Yes | Yes | Good |
| Fitzgerald & Williams, 2024 | Yes | Yes | Yes | Yes | Yes | Yes | Yes | Yes | Good |
| Fuchshuber et al., 2019 | Yes | Yes | Yes | No | Yes | Yes | Yes | Yes | Good |
| Gardner et al., 2014 | Yes | Yes | Yes | No | Yes | Yes | Yes | No | Fair |
| Gause et al., 2022 | Yes | Yes | Yes | No | No | Yes | Yes | Yes | Good |
| Gerhant & Olajossy 2016 | Yes | Yes | Yes | No | Yes | Yes | Yes | No | Fair |
| Glück et al., 2017 | Yes | Yes | Yes | No | No | Yes | Yes | No | Fair |
| Grasser et al., 2025 | Yes | Yes | Yes | No | Yes | Yes | Yes | Yes | Good |
| Gunnlaugsson et al., 2011 | Yes | Yes | Yes | No | No | Yes | Yes | No | Fair |
| Hamstra & Fitzgerald, 2022 | Yes | Yes | Yes | No | Yes | Yes | Yes | Yes | Good |
| Harper et al., 2004 | Yes | Yes | Yes | No | Yes | Yes | Yes | No | Good |
| Hasler et al., 2012 | Yes | Yes | Yes | Yes | Yes | Yes | Yes | Yes | Good |
| Henriksen et al., 2021 | Yes | Yes | Yes | No | Yes | Yes | CD | Yes | Good |
| Herrenkohl et al., 2012 | Yes | Yes | Yes | No | Yes | No | Yes | Yes | Fair |
| Hobfoll et al., 2002 | Yes | Yes | Yes | No | Yes | Yes | Yes | Yes | Good |
| Johnsona et al., 2002 | Yes | Yes | Yes | No | Yes | Yes | Yes | Yes | Good |
| Keene & Epps, 2016 | Yes | Yes | Yes | No | Yes | Yes | Yes | Yes | Good |
| Kendra, Bell, & Guimond, 2012 | Yes | Yes | Yes | No | No | Yes | Yes | No | Fair |
| Kobulsky et al., 2018 | Yes | Yes | Yes | No | No | Yes | Yes | Yes | Good |
| Leban & Delacruz, 2023 | Yes | Yes | Yes | No | Yes | Yes | Yes | Yes | Good |
| Li et al., 2025 | Yes | Yes | Yes | No | No | Yes | No | No | Fair |
| Litrownik et al., 2005 | Yes | Yes | Yes | No | Yes | Yes | Yes | Yes | Good |
| Macarenco et al., 2021 | Yes | Yes | Yes | No | Yes | Yes | Yes | Yes | Good |
| Malvaso et al., 2017 | Yes | Yes | Yes | No | Yes | Yes | Yes | No | Fair |
| Marie-Mitchell et al., 2020 | Yes | Yes | Yes | No | Yes | No | No | No | Poor |
| Mills et al., 2020 | Yes | Yes | Yes | No | Yes | Yes | Yes | Yes | Good |
| Moore et al., 2020 | Yes | Yes | Yes | No | Yes | Yes | Yes | No | Fair |
| Morandotti et al., 2013 | Yes | Yes | Yes | No | Yes | Yes | Yes | Yes | Good |
| Murphy & Turgoose 2022 | Yes | Yes | Yes | No | Yes | Yes | Yes | No | Fair |
| Nakai et al., 2014 | Yes | Yes | Yes | No | Yes | Yes | Yes | Yes | Good |
| Novaco & Taylor 2008 | Yes | Yes | Yes | No | Yes | No | Yes | Yes | Good |
| Osvath et al., 2004 | Yes | Yes | Yes | No | Yes | Yes | Yes | Yes | Good |
| Otsuka et al., 2017 | Yes | Yes | Yes | No | Yes | Yes | Yes | Yes | Good |
| Papalia et al., 2022 | Yes | Yes | Yes | No | Yes | Yes | Yes | Yes | Good |
| Peng et al., 2022 | Yes | Yes | Yes | No | Yes | Yes | Yes | Yes | Good |
| Perroud et al., 2010 | Yes | Yes | Yes | Yes | No | Yes | Yes | Yes | Fair |
| Peterson et al., 2018 | Yes | Yes | Yes | No | Yes | Yes | Yes | Yes | Good |
| Plattner et al., 2007 | Yes | Yes | No | No | Yes | Yes | Yes | Yes | Good |
| Porcerelli et al., 2015 | Yes | Yes | Yes | No | Yes | Yes | Yes | No | Fair |
| Qirjako et al., 2021 | Yes | Yes | Yes | No | No | Yes | Yes | Yes | Good |
| Quenneville et al., 2020 | Yes | Yes | Yes | No | Yes | Yes | Yes | Yes | Good |
| Rapoza et al., 2014 | Yes | Yes | Yes | No | Yes | Yes | Yes | Yes | Good |
| Renner et al., 2020 | Yes | Yes | Yes | No | No | Yes | Yes | Yes | Fair |
| Rinne-Albers et al., 2016 | Yes | Yes | No | No | No | No | Yes | Yes | Poor |
| Rizzo et al., 2014 | Yes | Yes | Yes | No | No | Yes | Yes | Yes | Good |
| Roe-Sepowitz & Krysik, 2008 | Yes | Yes | Yes | No | Yes | Yes | Yes | No | Fair |
| Schneider et al., 2005 | Yes | Yes | Yes | No | Yes | Yes | Yes | Yes | Good |
| Sebre et al., 2004 | Yes | Yes | Yes | No | Yes | Yes | Yes | No | Fair |
| Sevenoaks et al., 2022 | Yes | Yes | Yes | No | Yes | Yes | Yes | Yes | Good |
| Showalter et al., 2020 | Yes | Yes | Yes | No | Yes | Yes | Yes | Yes | Good |
| Sigfusdottir et al., 2013 | Yes | Yes | Yes | No | Yes | Yes | Yes | Yes | Good |
| Snyder et al., 2024 | Yes | Yes | Yes | No | Yes | Yes | Yes | Yes | Good |
| Solakoglu et al., 2018 | Yes | Yes | Yes | No | Yes | Yes | Yes | Yes | Good |
| Springer et al., 2007 | Yes | Yes | Yes | No | Yes | Yes | Yes | Yes | Good |
| Stearns & McKinney 2020 | Yes | Yes | Yes | No | Yes | Yes | Yes | Yes | Good |
| Sudbrack et al., 2015 | Yes | Yes | Yes | No | Yes | Yes | Yes | Yes | Good |
| Tang et al., 2017 | No | Yes | Yes | No | Yes | Yes | Yes | Yes | Fair |
| Teicher et al., 2006 | Yes | Yes | Yes | No | Yes | No | Yes | Yes | Fair |
| Teicher & Vitaliano, 2011 | Yes | Yes | CD | No | Yes | No | Yes | Yes | Fair |
| Toda et al., 2018 | Yes | Yes | Yes | No | Yes | Yes | Yes | No | Fair |
| Truhan & Armour 2023 | Yes | Yes | Yes | No | Yes | Yes | Yes | Yes | Good |
| Turner et al., 2006 | Yes | Yes | Yes | No | Yes | Yes | Yes | Yes | Good |
| van Vugt et al., 2014 | Yes | Yes | Yes | No | Yes | Yes | Yes | Yes | Good |
| Veith et al., 2017 | Yes | Yes | Yes | No | Yes | Yes | Yes | No | Fair |
| Wakatsuki et al., 2020 | Yes | Yes | Yes | No | Yes | Yes | Yes | No | Fair |
| Walker et al., 2021 | Yes | Yes | Yes | Yes | No | No | Yes | Yes | Fair |
| Wang et al., 2020 | Yes | Yes | Yes | No | Yes | Yes | Yes | Yes | Good |
| White et al., 2016 | Yes | Yes | Yes | No | Yes | No | Yes | Yes | Fair |
| Wills et al., 2022 | Yes | Yes | Yes | No | Yes | Yes | Yes | No | Fair |
| Win et al., 2022 | Yes | Yes | Yes | No | Yes | Yes | Yes | No | Fair |
| Wolfe et al., 2001 | Yes | Yes | Yes | No | Yes | Yes | Yes | Yes | Good |
| Wuebken et al., 2023 | Yes | Yes | Yes | No | Yes | Yes | Yes | Yes | Good |
| Xu et al., 2024 | Yes | Yes | Yes | No | Yes | Yes | Yes | Yes | Good |
| Yarar et al., 2022 | Yes | Yes | Yes | No | Yes | Yes | Yes | Yes | Good |
| Yaşar et al., 2020 | Yes | Yes | Yes | No | Yes | Yes | Yes | No | Good |
| Zelikovsky & Lynn, 2002 | Yes | Yes | Yes | No | Yes | Yes | Yes | No | Fair |
| Zhou & Zhen, 2022 | Yes | Yes | Yes | No | Yes | Yes | Yes | No | Good |

*Note.* Overall rating includes either a poor, fair, or good quality assessment, reflecting the number of ‘yes’ responses out of the total number of applicable questions for each study. Studies with 7+ were rated ‘good’, 5-6 were rated ‘fair, and 4 or less were rated ‘poor’.

**Table S3. Included NIH Risk of Bias and Quality Assessment Items**

| **Item Number** | **Item Description** |
| --- | --- |
| 1 | Was the research question or objective in this paper clearly stated? |
| 2 | Was the study population clearly specified and defined? |
| 3 | Were all the subjects selected or recruited from the same or similar populations (including the same time period)? Were inclusion and exclusion criteria for being in the study prespecified and applied uniformly to all participants? |
| 4 | Was a sample size justification, power description, or variance and effect estimates provided? |
| 5 | For exposures that can vary in amount or level, did the study examine different levels of the exposure as related to the outcome (e.g., categories of exposure, or exposure measured as continuous variable)? |
| 6 | Were the exposure measures (independent variables) clearly defined, valid, reliable, and implemented consistently across all study participants? |
| 7 | Were the outcome measures (dependent variables) clearly defined, valid, reliable, and implemented consistently across all study participants? |
| 8 | Were key potential confounding variables measured and adjusted statistically for their impact on the relationship between exposure(s) and outcome(s)? |

*Note.* Assessment tool can be accessed at: <https://www.nhlbi.nih.gov/health-topics/study-quality-assessment-tools>

**Table S4. Characteristics of Included Studies**

| **Study** | **Developmental Stage** | **Country** | **Age** | **Gender or Sex^1^** | **Race and Ethnicity^1^** | **Recruitment Source** |
| --- | --- | --- | --- | --- | --- | --- |
| Adler et al., 2020 [[35]](https://www.zotero.org/google-docs/?rKpRNe) | Adults | USA | No mean reported, range: 18-77 | 70.5% male, 29.5% female | 73% Non-Hispanic White, 10.5 % Non-Hispanic Black, 16.5% other | Randomly selected from USA military rosters |
| Almeida et al., 2024 [[27]](https://www.zotero.org/google-docs/?yWYM9D) | Adults | Portugal | Mean: 31.4, range: 18-77 | 29.9% male, 70.1% female | 93.4% Caucasian | Online |
| Asgeirsdottir et al., 2011 [[16]](https://www.zotero.org/google-docs/?QHCSXf) | Adolescents and young adults | Iceland | Mean: 17.2, range: 16-19 | 49% male, 51% female | Not reported | All high schools in Iceland |
| Baron, 2019 [[36]](https://www.zotero.org/google-docs/?K9xVVL) | Adolescents and young adults | Canada | Mean: 20.1, range: 16-24 | 64% male, 36% female | 47.5% White, 19.5% Black Canadian, 8% Aboriginal, 17.7% Asian Canadian/Indo-Canadian, Hispanic, or Biracial | Homeless youth in Toronto recruited from the street, shelters, and drop-in centers |
| Barrett et al., 2013 [[37]](https://www.zotero.org/google-docs/?iMnKsY) | Adults | Australia | Mean: 35.1, range not reported | 77.6% male, 22.4% female | Not reported | An inpatient detoxification clinic in Sydney, Australia |
| Beesley et al., 2010 [[38]](https://www.zotero.org/google-docs/?CBIZoL) | Adults | UK | Mean: 41.6 for irritable bowel syndrome group and 37.8 for Crohn's disease group, range not reported | 64.2% male, 35.8% female | Not reported | Patients attending outpatient clinics with a diagnosis of irritable bowel syndrome or Crohn's disease |
| Benedini & Fagan, 2020 [[22]](https://www.zotero.org/google-docs/?o5P1M0) | Adolescents | USA | Mean: 4.5 at baseline and followed to age 16 | 51% female, 49% male | 52% African American, 26% White, 8% Hispanic, and 15% other racial/ethnic groups | LONGSCAN |
| Berthelot et al., 2014 [[39]](https://www.zotero.org/google-docs/?zlMywZ) | Adults | Canada | Mean: 35, range: 19-80 | 53.5% women, 46.5% men | Not reported | Patients consulting for sexual dysfunction or dissatisfaction from different clinics |
| Bielas et al., 2016 [[40]](https://www.zotero.org/google-docs/?QjAgLV) | Adolescents and young adults | Switzerland | Mean: 16.84, range 13.8-19.5 | All male | Not reported | Youth in Zurich Juvenile Detention Centre |
| Bilgi et al., 2017 [[31]](https://www.zotero.org/google-docs/?pGI8Kq) | Adults | Turkey | Mean: 33.8 for schizophrenia group and 33.7 for healthy control group, range not reported | 80.6% male, 19.4% female | Not reported | An inpatient psychiatry unit and healthy age-, gender-, and education-matched controls |
| Bock et al., 2020 [[41]](https://www.zotero.org/google-docs/?jtVKiV) | Adults | Brazil | Mean: 23.7, range: 18-35 | 52.3% female, 47.7% male | 75.1% White | Population-based study carried out in the urban area of Pelotas, Southern Brazil |
| Boonmann et al., 2016 [[12]](https://www.zotero.org/google-docs/?dpWSLH) | Adolescents and young adults | Netherlands | Mean: 17 for sexually-offending juveniles (JSOs) and 17.7 for non-JSOs; range: 13-24 | All male | Not reported | Male youth recruited from 2 juvenile detention centers in the Netherlands |
| Brenner et al., 2018 [[42]](https://www.zotero.org/google-docs/?i1PTLU) | Children | USA | Mean: 12.9, range: 4-21 | 79% male, 21% female | 79% White | Psychiatric specialty hospitals as part of the autism inpatient collection study |
| Brezina, 1998 [[43]](https://www.zotero.org/google-docs/?XUFUMc) | Adolescents | USA | Mean: 15 at wave 1 | All boys | Not reported | Youth in Transition (YIT) survey (1.5 years apart), 10-11th grades |
| Briere et al., 2001 [[13]](https://www.zotero.org/google-docs/?Fab1Hk) | Youth and their caregivers | USA | Mean: 7.1, range not reported | 62.8% female, 37.2% male | 38.3% Non-Hispanic White, 25.4% Black, 27.8% Hispanic | Data acquired from researchers across the United States |
| Carver et al., 2014 [[44]](https://www.zotero.org/google-docs/?ymd2Ez) | Young adults | USA | Mean: 18.7, range not reported | 65.2 female, 34.8% male | 57.1% Non-Hispanic white, 24.1% Hispanic, 7.5% Asian, 4.5% African American, 2.3% Caribbean, and 4.5% other | Undergraduates at the University of Miami |
| Cascardi et al., 2020 [[45]](https://www.zotero.org/google-docs/?QuxrIV) | Young adults | USA | Mean not reported, range: 18-21 | 63.9% female, 36.1% male | 50% Non-Hispanic White, 16.2% Non-Hispanic Black, 22.9% Hispanic of any race, 10.9% Asian, Middle Eastern, or multi-racial/ethnic | A regional state university in the northeast United States |
| Cazala et al., 2019 [[23]](https://www.zotero.org/google-docs/?fslN1M) | Children and adolescents | USA | Mean: 13.8, range: 6-17 | 50.8% female, 49.2% male | Not reported | Advertisements and physician referrals from clinics in Houston and San Antonio, Texas, and Chapel Hill, North Carolina |
| Cecil et al., 2014 [[46]](https://www.zotero.org/google-docs/?JBTWKS) | Adolescents and young adults | UK | Mean: 18.9, range: 16-24 | 53% female, 47% male | 44% Caucasian, 41% Black, 10% Mixed, and 5% Asian | Community groups and secondary schools in London |
| Choi et al., 2009 [[47]](https://www.zotero.org/google-docs/?Vg6ith) | Adults | USA | Mean: 37.7 for no adversity group, 30.8 for adversity group, range not reported | 48.5% female, 51.5% male | 79% White, 18% African American, 3% Native American | Inpatient psychiatric rehabilitation research unit at a state psychiatric hospital |
| Chong et al., 2023 [[48]](https://www.zotero.org/google-docs/?230Vf5) | Adults | USA | Mean: 19.0, range not reported | 52.7% female, 47.3% male | 72% White, 11% Black, 10 % Hispanic, and 7 % biracial or other | Undergraduate students |
| Coe et al., 2020 [[17]](https://www.zotero.org/google-docs/?eckKMf) | Children | USA | Mean: 4.2, range: 3-5 | 52.2% female, 47.8% male | 40% White, 16% Black, 22% Biracial, and 19% Other Races, 46% Hispanic | Pediatric medical clinic during a well-child visit, childcare centers, and the state’s child protection agency |
| Collin-Vézina et al., 2011 [[49]](https://www.zotero.org/google-docs/?IElj4D) | Children and adolescents | Canada | Mean: 15.5, range not reported | 55% male, 45% female | 38% Caucasian, 19% Black, 21% Aboriginal (First Nations, Inuit, or Métis), 22% mixed or of another ethnic background | Residential care unit in Montreal |
| Cooley & Taussig, 2022 [[50]](https://www.zotero.org/google-docs/?l9EZtW) | Children | USA | Mean: 10.3, range: 8-11 | 47.3% girls, 52.7% boys | 50.6% White, 52.1% Hispanic/Latinx, 28.0% Black/African American, 9.8% other race/ethnicity | Children who had a dependency and neglect petition with out-of-home care filed in the previous year in participating counties |
| Cotter et al., 2024 [[51]](https://www.zotero.org/google-docs/?MayUyI) | Children and adolescents | USA | Mean: 9.7, range: 3-18 | 56.8% male, 43.2% female | 34.6% White, 37.5% Hispanic, 20.4% Black, 4.8% Multiracial, and 2.6% Other | Individuals enrolled in Fathers for Change, a psychotherapy intervention for fathers with IPV histories |
| de Bles et al., 2023 [[34]](https://www.zotero.org/google-docs/?EVxpSQ) | Adults | Netherlands | Mean: 42.1, range not reported | 66.2% female, 33.8% male | Not reported | Netherlands Study of Depression and Anxiety |
| Derella et al., 2020 [[52]](https://www.zotero.org/google-docs/?58Fj3J) | Children and adolescents | USA | Mean not reported, range: 5-8 at baseline and followed through age 16 | All female | 53% African-American, 41% White, and 6% Asian or multiracial | Pittsburgh Girls Study (PGS), recruited from neighborhoods where one-quarter of the residents were at or below the poverty level were enumerated, as were a random selection of 50% of the households in the remainder of city neighborhoods |
| DiLillo et al., 2000 [[53]](https://www.zotero.org/google-docs/?2IkgDZ) | Adults | USA | Mean: 25.8 for adversity-exposed group, 26.5 for non-exposed group, range not reported | All female | Adversity exposed group: 29% African American, 65% White, 6% other minority; non-exposed group 32% African American, 58% White, 10% other minority | Mom-Kid Trial Parenting Program; recruited primarily through regular presentations by our staff at local Women, Infants, and Children (WIC) clinics |
| Etchin et al., 2022 [[54]](https://www.zotero.org/google-docs/?DWm14b) | Adults | USA | Mean: 37.5, range not reported | 90.5% male, 9.5% female | 77.8% White | Veterans recruited from New England area |
| Evans et al., 2012 [[2]](https://www.zotero.org/google-docs/?obbvt9) | Children/Adolescents | USA | Mean: 10.5 at wave 1, range: 10-12 | 45.9% female, 54.1% male | All African American | Family and Community Health Study, a multi-site (Georgia and Iowa) investigation of neighborhood and family processes that contribute to African American children’s development in families living in a variety of community settings |
| Faulkner et al., 2014 [[55]](https://www.zotero.org/google-docs/?vt4Xyc) | Adolescents and young adults | Canada | Mean: 17.9, range: 16-19 | 62.7% female, 37.3% male | 25.8% Caucasian, 26.5% African or Caribbean Canadian, 30.3% mixed race, 17.4% Hispanic, Chinese, Aboriginal, South Asian, and West or South East Indian | Randomly selected from CPS caseloads (i.e., case management, resource linkages, out-of-home care, etc.) within a large urban catchment area in Ontario |
| Feiring et al., 2013 [[56]](https://www.zotero.org/google-docs/?oTsi0Z) | Children and adolescents | USA | Mean: 9.6, range: 8-11 at for children at baseline; mean: 13.5, range: 12-15 for adolescents at baseline | 73% female, 27% male | 41% African American, 31% White, 20% Hispanic (20%), 8% other | CPS offices or regional child abuse medical clinics |
| Fitzgerald, 2021 [[57]](https://www.zotero.org/google-docs/?OVWndu) | Adolescents | USA | Not reported; measures collected at age 12, 16, and 18 | 59.7% female, 40.3% male | 77.1% racial minorities | LONGSCAN; utilized data from adolescents who participated in waves of age 12, 16, and 18 and were in currently involved in a romantic relationship in the final wave (age 18) |
| Fitzgerald & Williams, 2024 [[58]](https://www.zotero.org/google-docs/?oQxYAe) | Adults | USA | Mean: 57.2, range: 25-75 | 50.2% female, 49.8% male | 93.8% White | Midlife in the United States study |
| Fuchshuber et al., 2019 [[59]](https://www.zotero.org/google-docs/?2susgm) | Adults | Austria | Mean: 30, range: 18-69 | 61.9% female, 38.1% male | Reported nationality: 54.5% German, 35.5% Austrian, 4.8% Swiss, 5.5% other nationalities | Online |
| Gardner et al., 2014 [[60]](https://www.zotero.org/google-docs/?4n7aXI) | Adults | USA | Mean: 34.5 for IPV offenders, 38 for non IPV offenders, range: 19-63 | IPV offenders: all males; non IPV offenders: 77% male, 33% female | IPV offenders: 49% Caucasian, 40% African American, 11% Other; non-IPV offenders: 63% African American, 19% Caucasian, 10% Other, and 8% Hispanic | Individuals mandated for psychological treatment from probation offices following violent offenses |
| Gause et al., 2022 [[61]](https://www.zotero.org/google-docs/?mQEDvp) | Young adults | USA | Mean: 20.3, range: 18-24 | All female | All Black | Recruited via community outreach in a large Southeastern US city and screened for eligibility to enroll in a randomized HIV-risk reduction intervention trial |
| Gerhant & Olajossy, 2016 [[62]](https://www.zotero.org/google-docs/?KZWpn3) | Adults | Poland | Mean: 37.2, range not reported | 44.4% women, 55.6 men | Not reported | Individuals addicted to alcohol and receiving in-patient detoxification treatment |
| Glück et al., 2017 [[63]](https://www.zotero.org/google-docs/?aDjPUh) | Adults | Austria | Mean: 58.0, range: 29-87 | 59.8% men, 40.2% women | 25.8% Caucasian, 26.5% African or Caribbean Canadian, 30.3% mixed race, 17.4% Hispanic, Chinese, Aboriginal, South Asian, and West or South East Indian | Vienna Institutional Abuse Study, included survivors of institutional abuse in care settings in Vienna between the years 1946 and 1986 |
| Grasser et al., 2024 [[64]](https://www.zotero.org/google-docs/?FYxkFP) | Children and adolescents | USA | Mean: 14.9, range: 9-19 | 35.4% girls, 64.6% boys | 12.8% Black, 5.1% Asian/Pacific Islander, 7% White, 7.7% Biracial, 35.9% Other; 66.7% Hispanic/Latin, 33.3% Not Hispanic/Latin | Two cohorts of treatment-seeking youth in the San Diego area |
| Gunnlaugsson et al., 2011 [[65]](https://www.zotero.org/google-docs/?exLHSb) | Adolescents | Iceland | Mean not reported, range: 14-15 | 48.8% female, 51.2% male | Not reported | International European School Project on Alcohol and other Drugs survey, included 14-15 year old students |
| Hamstra & Fitzgerald, 2022 [[66]](https://www.zotero.org/google-docs/?ZokE0k) | Adolescents | USA | Mean and range not reported, assessed at ~age 12 | 50.6% female, 49.4% male | 61.1% African American, Hispanic, Asian, Native American, or multiracial, 38.9% White | LONGSCAN |
| Harper & Arias, 2004 [[26]](https://www.zotero.org/google-docs/?KKuWhq) | Young adults | USA | Mean: 19, range not reported | 59% female, 41% male | 85% White | Undergraduate students recruited from introductory psychology courses at a large Southeastern university |
| Hasler et al., 2012 [[67]](https://www.zotero.org/google-docs/?GdQMZ2) | Adults | France and Switzerland | Mean: 39.8 for participants with a suicide attempt, 42.85 for participants without an attempt, range: 18-75 | All male | All of European ancestry | Psychiatric units of 2 hospitals in Montpellier, France and Geneva, Switzerland or Blood Donor Centers at the University of Geneva |
| Henriksen et al., 2021 [[18]](https://www.zotero.org/google-docs/?mBRG6V) | Adolescents and young adults | Norway | Mean: 16.1  range: 13-19 | 48.7% boys, 51.3% girls | Not reported | Nord Trøndelag Health Study (HUNT), included adolescents in the County Nord-Trøndelag, Norway |
| Herrenkohl et al., 2012 [[68]](https://www.zotero.org/google-docs/?yhRfos) | Adults | USA | Mean: 36, range: 31-41 | 47.9% females, 52.1% males | 1.3% American Indian/Alaska Native, 0.2% Native Hawaiian or Other Pacific Islander, 5.3% Black or African American, 80.7% White, 11.2% more than one race, 1.3% unknown, 7% Hispanic or Latino | Children in welfare system in Pennsylvania with at least one case of abuse or neglect of a child 18 months to 6 years old or children in Eastern Pennsylvania area with no cases |
| Hobfoll et al., 2002 [[69]](https://www.zotero.org/google-docs/?UhTYAc) | Adults | USA | Mean: 19.7, range not reported | All women | all Native American | Community centers at Little Big Horn, Dull Knife, Blackfoot, and Fort Belknap reservations in Montana |
| Johnsona et al., 2002 [[70]](https://www.zotero.org/google-docs/?DUjkv7) | Children | USA | Measures collected at ages 6 and 8 | 44% male, 56% female | 64% Non-White, 36% White | Stress, Social Support, and Abuse & Neglect in High Risk Infants Study, recruited from North Carolina hospitals and health departments; also part of LONGSCAN |
| Keene & Epps, 2016 [[71]](https://www.zotero.org/google-docs/?tLN4oY) | Adults | USA | Mean: 21.4, range: 18-49 | 80.3% female, 19.7% male | Not reported | Undergraduate psychology class at a large, Southern US university |
| Kendra et al., 2012 [[72]](https://www.zotero.org/google-docs/?fgMX2w) | Adults | USA | Mean: 18.8, range not reported | All female | 53.9% White, 28.5% African-American, 8.2% Asian, 6.1% Hispanic, 2.9% American Indian, and 0.4% Other | Undergraduate students at a large Midwestern university |
| Kobulsky et al., 2018 [[19]](https://www.zotero.org/google-docs/?ksGaiu) | Adolescents | USA | Not reported, measures collected at ages 12 and 16 | 47.5% boys, 52.5% girls | Not reported | LONGSCAN, included data at waves of age 12 and 16 |
| Leban & Delacruz, 2023 [[24]](https://www.zotero.org/google-docs/?6unbLz) | Adolescents | USA | Not reported, measures collected at ages 12 and 16 | 57% girls, 43% boys | 46.27% Black, 29.42% White, 7.76% Hispanic, 16.55% other racial/ethnic category | LONGSCAN, included participants who remained in the study through the age 18 survey and had complete data on ACEs, mediating emotional states, and violent delinquency |
| Li et al., 2025 [[73]](https://www.zotero.org/google-docs/?tGg6x3) | Adolescents | USA | Not reported | 81.2% male, 18.8% female | 46.9% Black and Hispanic, 8.1% White, 0.7% Asian | Archival data from the Harris County Juvenile Probation Department in Houston, Texas |
| Litrownik et al., 2005 [[74]](https://www.zotero.org/google-docs/?taEjKo) | Children | USA | Mean: 8.2, range not reported | 49.1% male, 50.9% female | 31.8% White, 68.2% Minority | LONGSCAN; included 5 samples recruited for being at-risk or identified as being maltreated |
| Macarenco et al., 2021 [[75]](https://www.zotero.org/google-docs/?z8CfCh) | Adults | Romania | Mean: 39.9, range not reported | 79.1% female, 20.9% male | Not reported | Online recruitment via Facebook |
| Malvaso et al., 2017 [[76]](https://www.zotero.org/google-docs/?jNEExp) | Adolescents | Australia | Mean: 16.0, range: 14-18 | All male | 53.6% Aboriginal or Torres Strait Islander | Juvenile justice detention center in South Australia |
| Marie-Mitchell et al., 2020 [[77]](https://www.zotero.org/google-docs/?Tpuhfz) | Children | USA | Mean not reported, range: 5-11 | 49.1% female, 50.9% male | Not reported | Retrospective chart review from two university-affiliated clinics in California |
| Mills et al., 2020 [[78]](https://www.zotero.org/google-docs/?KLejKR) | Children | USA | Mean: 10.9, range: 9-12 | 58 female, 56 male | 85% White, 5% Native American, 3% African American, 1% Pacific Islander, 1% Asian, 5% Other, 27% Hispanic/Latinx | Selected from a larger sample of 9–14 year olds enrolled in the Developmental Chronnecto-Genomics study |
| Moore et al., 2020 [[79]](https://www.zotero.org/google-docs/?IqRzkQ) | Adults | USA | Mean: 19.2, range: 18-35 | 73% female | 72% White | Undergraduates from a mid-sized university in the Rocky Mountain region of the United States |
| Morandotti et al., 2013 [[32]](https://www.zotero.org/google-docs/?d7JRDS) | Adults | Italy | Mean 34.2 for patients and 32.5 for healthy controls, range not reported | 70.3% female, 29.7% male | Not reported | Center for Research on Personality Disorders of the University of Pavia, Italy |
| Murphy & Turgoose, 2022 [[80]](https://www.zotero.org/google-docs/?wPFXA6) | Adults | UK | Not reported | 96% male | Not reported | Combat Stress, a national charity providing specialist mental health services to military veterans in the UK |
| Nakai et al., 2014 [[81]](https://www.zotero.org/google-docs/?3CIM3v) | Adults | Japan | Mean: 42.4, range not reported | 58.2% male, 41.8% female | Not reported | General adult population in Japan |
| Novaco & Taylor, 2008 [[82]](https://www.zotero.org/google-docs/?g74Ez4) | Adults | UK | Mean: 33.2, range not reported | All male | All White | Hospital forensic service of the National Health Service Trust in England |
| Osvath et al., 2004 [[83]](https://www.zotero.org/google-docs/?reUzFC) | Adults | Hungary | Mean: 33.4, range: 17-75 | 62% female, 38% male | Not reported | General and psychiatric hospitals in Pécs, Hungary (apart of a multicenter study on suicidal behavior) |
| Otsuka et al., 2017 [[84]](https://www.zotero.org/google-docs/?ER6PRW) | Adults | Japan | Mean: 42.3, range not reported | 53.5% male, 46.5% female | Not reported | General adult population in Japan |
| Papalia et al., 2022 [[85]](https://www.zotero.org/google-docs/?sL3TRu) | Adolescents | Australia | Mean: 16.8, range not reported | 82% male, 18% female | 48.8% White, 31.2% non-English speaking minority group, 20.0% Indigenous | Two justice centers in Victoria, Australia |
| Peng et al., 2022 [[86]](https://www.zotero.org/google-docs/?MILkCy) | Adolescents | China | Mean: 15.2, range: 12-18 | 51.9% male, 48.1% female | Not reported | High schools from 5 provinces in China |
| Perroud et al., 2010 [[87]](https://www.zotero.org/google-docs/?SpZLMH) | Adults | France and Switzerland | Mean: 39.6, range not reported | 29.3 % male, 70.7% female | All of European ancestry | Psychiatric units of 3 hospitals in Montpellier and Créteil, France, and Geneva, Switzerland |
| Peterson et al., 2018 [[88]](https://www.zotero.org/google-docs/?L8z8u6) | Adults | USA | Mean: 23.6, range not reported | All male | 78% Black/African American, 16% White/European American, 0.5% American Indian/Native American/Alaskan Native, 0.3% Asian/Asian American, 3% Hispanic/Latino | Two urban clinics in St. Louis, Missouri and Indianapolis, Indiana |
| Plattner et al., 2007 [[89]](https://www.zotero.org/google-docs/?7TPDUT) | Children and adolescents | USA | Mean: 14.5 and range: 11-16 for clinical group, mean: 16.5 and range: 14-18 for comparison group | Clinical group: 51.9% female, 48.1% male  Comparison group: 54.9% female, 45.1% male | clinical group: 19.2% Caucasian, 38.5% Hispanic, 23.1% African American, 1.9% Asian American, 11.5% Multiracial, 5.8% other; comparison group: 60.3% Caucasian, 18.4% Asian, 3.6 % Hispanic, 2.2% African American, and 15.5% other | Post-adjudicated participants recruited from a local juvenile hall; comparison group was recruited from two local high schools |
| Porcerelli et al., 2015 [[3]](https://www.zotero.org/google-docs/?j3DTZI) | Adults | USA | Mean: 34.3, range not reported | All women | All African American | Larger study on women's health through a university-based primary care clinic |
| Qirjako et al., 2021 [[90]](https://www.zotero.org/google-docs/?hIm0tw) | Adolescents | Albania | All 15 years old | 53.7% girls, 56.3% boys | Not reported | Nationwide representative sample of youth aged 15 in Albania |
| Quenneville et al., 2020 [[91]](https://www.zotero.org/google-docs/?nHZrfW) | Adults | Switzerland | Mean: 31.7, range not reported | 92.3% female, 7.8% male | Not reported | Specialized unit at the Geneva University Hospital providing healthcare to outpatients with borderline personality disorder |
| Rapoza et al., 2014 [[92]](https://www.zotero.org/google-docs/?CK1SOE) | Adults | USA | Not reported | 63% women, 37% men | 49% African American, 30% Caucasian, 21% Latino | Non-traditional college, where students were primarily older, working while obtaining a degree, and commuters |
| Renner et al., 2020 [[93]](https://www.zotero.org/google-docs/?NPcu50) | Children and adolescents | USA | Mean: 13.6, range: 10-17 | 49.6% male, 50.4% female | 74.97% Non-Hispanic White, 17.24% Non-Hispanic Black, 3.18% Asian, 3.72% other/multiple race, 10% Hispanic | Developmental Victimization Survey, a randomly selected nationally representative survey of youth |
| Rinne-Albers et al., 2016 [[30]](https://www.zotero.org/google-docs/?jlocBI) | Adolescents and young adults | Netherlands | Mean: 16.5 for PTSD group and 15.4 for control group, range: 12-21 | 88% female, 12% male | Not reported | Emotional Pathways Imaging Study in Clinical Adolescents (EPISCA), recruited mostly from specialized psychotrauma centers |
| Rizzo et al., 2014 [[20]](https://www.zotero.org/google-docs/?bkpzoS) | Adolescents | USA | Mean: 15.1, range not reported | 76% female, 24% male | 81% Caucasian/European American, 11% Hispanic, 3% Asian American, 3% African American, and 2% mixed ethnicity-other | Inpatient unit of a regional children’s psychiatric hospital in southern New England |
| Roe-Sepowitz & Krysik, 2008 [[94]](https://www.zotero.org/google-docs/?i0YaW3) | Adolescents | USA | Mean: 13.9, range: 7-17 | All female | 61% White, 33.1% African American, 5.9% Other | Statewide juvenile offender database (charged and sanctioned from 1999 to 2005) in Florida |
| Schneider et al., 2005 [[95]](https://www.zotero.org/google-docs/?kZ6qZS) | Children | USA | Measures collected at age 8 | Not reported | Not reported | LONGSCAN; including 5 samples identified as at-risk or maltreated |
| Sebre et al., 2004 [[96]](https://www.zotero.org/google-docs/?AXvmku) | Children and adolescents | Latvia, Lithuania, Macedonia, and Moldova | Mean not reported, range: 10-14 | Approximately 50% boys, 50% girls | Not reported | Primary schools in Latvia, Lithuania, Macedonia, and Moldova |
| Sevenoaks et al., 2022 [[97]](https://www.zotero.org/google-docs/?iRkYiw) | Adolescents | South Africa | Mean: 13.5 years for youth with HIV and 13.8 for control group, range not reported | HIV group: 45.4% males, 55.6% females; control group: 50% males, 50% females | Not reported | Neuropsychiatric sub-study of the Cape Town Adolescent Antiretroviral Cohort study |
| Showalter et al., 2020 [[98]](https://www.zotero.org/google-docs/?SghXCg) | Adolescents | USA | Mean: 13.5, range not reported | 52.4% girls, 47.6% boys | Not reported | Patients in a child and adolescent mental health services outpatient unit |
| Sigfusdottir et al., 2013 [[21]](https://www.zotero.org/google-docs/?JPfxGl) | Adolescents and young adults | Iceland | Mean: 17.2, range: 16-19 | 51% female, 49% male | Not reported | Cross-sectional national survey of 9085 16–19-year-old students at all high schools in Iceland in the year 2004 |
| Snyder et al., 2024 [[99]](https://www.zotero.org/google-docs/?vylXEj) | Adults | USA | Mean: 21.9, range: 18–40 | All male | 74.1 % White, 15.6% Black, 8.9% Asian, 2.2% Native Hawaiian or Pacific Islander, 3.3% American Indian or Alaska Native, 21.3% Hispanic | Active-duty enlisted members of the Air Force from a larger longitudinal study on military couples |
| Solakoglu et al., 2018 [[100]](https://www.zotero.org/google-docs/?n2m0xu) | Adolescents | Turkey | Mean: 16.6, range not reported | 49% male, 51% female | Not reported, all Turkish nationality | Adolescents in Istanbul |
| Springer et al., 2007 [[101]](https://www.zotero.org/google-docs/?9ewuSY) | Adults | USA | Mean: 55, range not reported | abused group: 55.6% female, 44.4% male; non-abused group: 51.9% female, 48.1% male | Not reported, described as "almost entirely white of Non-Hispanic origin" | Sibling respondents of the Wisconsin Longitudinal Study (WLS), a long-term population-based study of men and women who graduated in 1957 from Wisconsin high schools and their siblings |
| Stearns & McKinney, 2020 [[28]](https://www.zotero.org/google-docs/?vbmcpO) | Adults | USA | Mean: 19.6, range: 18-25 | 34.6% male, 65.4% female | 70.1% White, 23.5% Black, 2.1% Hispanic, 1.9% Asian, 2.2% other | A Southern United States university |
| Sudbrack et al., 2015 [[29]](https://www.zotero.org/google-docs/?Bz4fhN) | Adults | Brazil | Mean: 27.7 for males and 28.0 for females, range not reported | 30.2% male, 69.8% female | 67% White | National media broadcast (TV news, major newspapers) |
| Tang et al., 2017 [[102]](https://www.zotero.org/google-docs/?aW7JCe) | Adolescents and young adults | China | Mean: 20.8, range: 16-28 | All male | Not reported | Youth/young adults in custody at a youth correctional facility in Hubei province |
| Teicher et al., 2006 [[103]](https://www.zotero.org/google-docs/?MoPJBf) | Adults | USA | Mean: 19.8, range: 18-22 | 378 women, 176 men | 73% White, 6% Black, 6% Hispanic, 10% Asian, 1% Native American, 4% other ethnic groups | Advertisements to young adults requesting healthy subjects or individuals with a history of an unhappy childhood |
| Teicher & Vitaliano, 2011 [[104]](https://www.zotero.org/google-docs/?skQZuf) | Adults | USA | Mean: 21.8, range not reported | 37.3% male, 62.7% female | 75% Non-Hispanic White, 9% Non-Hispanic Black, 6% Non-Hispanic Asian, 4% Non-Hispanic other race, 8% Hispanic of any race | Multi-study community database of participants who had siblings |
| Toda et al., 2018 [[33]](https://www.zotero.org/google-docs/?GgWH1d) | Adults | Japan | Mean: 47.3 for bipolar group and 44.2 for control group, range not reported | Bipolar group: 56% male, 44% female; control group: 60.6% male, 39.4% female | Not reported | Outpatients with bipolar disorder (BD) recruited from Department of Psychiatry, National Defense Medical College, Hokkaido University Hospital, Self-Defense Forces Sapporo Hospital, and Self-Defense Forces Central Hospital  Healthy controls recruited from the general population |
| Truhan & Armour, 2023 [[105]](https://www.zotero.org/google-docs/?JfOYOR) | Adults | UK/Ireland | Mean: 56, range: 25-99 | All male | 99.3% White | Northern Ireland Veterans Health and Wellbeing Study (NIVHWS) |
| Turner et al., 2006 [[106]](https://www.zotero.org/google-docs/?jJL3bx) | Children | USA | Not reported | Not reported | Not reported | Nationally representative sample of youth living in United States |
| van Vugt et al., 2014 [[107]](https://www.zotero.org/google-docs/?IkjwN9) | Adolescents and young adults | Canada | Mean: 19.3, range not reported | All female | Not reported | Larger study on youth in residential care in Montreal |
| Veith et al., 2017 [[108]](https://www.zotero.org/google-docs/?HIuFxK) | Adults | USA | Mean not reported, range: 18-59+ | 67.1% women, 32.9% men | 77.3% Caucasian, 6.9% African American, 4.4% Hispanic, 4.6% Bi-racial/Multi-racial, 3.4% Asian American, 1.5% Native American, 1.9% Other | Amazon's Mechanical Turk |
| Wakatsuki et al., 2020 [[109]](https://www.zotero.org/google-docs/?MSMzj0) | Adults | Japan | Mean: 32.4, range not reported | All female | Not reported | Japanese adult volunteers from the community |
| Walker et al., 2021 [[110]](https://www.zotero.org/google-docs/?s4U11T) | Adults | USA | Mean: 20.8, range: 18-24 | 81.1% women, 18.3% men | 56.6% White, 26.2% Black, 6% Biracial/Multiracial, 4.9% Asian, 2.3% Middle Eastern, 2.6% other, 5.6% Hispanic/Latino | Undergraduate students from two U.S. universities |
| Wang et al., 2020 [[111]](https://www.zotero.org/google-docs/?HveYJ9) | Adolescents | China | Mean: 13.6, range: 11-16 | 52.4% girls, 47.6% boys | Not reported | 7th and 8th grade classes in Taiyuan, China |
| White et al., 2016 [[112]](https://www.zotero.org/google-docs/?t6TVOy) | Adolescents and young adults | USA | Mean: 18.4, range not reported | 45.2 % male, 54.8% female | 26% White, 55.2% African American, 6.4% Hispanic, 12.5% Other | LONGSCAN |
| Wills et al., 2022 [[4]](https://www.zotero.org/google-docs/?XSg6Pg) | Adults | USA | Mean: 47.8, range not reported | All female | All Hispanic/Latino | Sexual Assault Among Latinas Study |
| Win et al., 2021 [[113]](https://www.zotero.org/google-docs/?Qrd2NT) | Adults | USA | Mean: 45.6 at baseline, range: 24-74 | 54.6% female, 45.4% male | 89.01% White, 10.99% African American, Asian, Native American, or Pacific Islander | Midlife Development in the United States (MIDUS) project |
| Wolfe et al., 2001 [[25]](https://www.zotero.org/google-docs/?CDGTqq) | Adolescents and young adults | Canada | Mean: 16.1, range: 14-19 | 55% female, 45% male | 79% White (79%), 6% Pacific rim, 5% Aboriginal, 2% African descent, 8% other or unknown ethnicity | 10 high schools in Southwestern Ontario |
| Wuebken et al., 2023 [[114]](https://www.zotero.org/google-docs/?143yfi) | Adults | Germany | Mean: 38.9, range not reported | All female | Not reported, 91.7% were of German nationality | Two German cities, Berlin and Heidelberg, recruited by advertisement |
| Xu et al., 2024 [[115]](https://www.zotero.org/google-docs/?UQTTcE) | Adolescents | China | Mean: 13.5, range not reported | 52.5% male, 47.5% female | Not reported | Middle schools in the Anhui Province, China |
| Yarar et al., 2023 [[116]](https://www.zotero.org/google-docs/?HJBzTo) | Adults | Turkey | Mean: 21.4, range: 18-34 | 15.6% male, 84.4% female | Not reported | Education department of a university in Ankara, Turkey |
| Yaşar et al., 2020 [[117]](https://www.zotero.org/google-docs/?zjGeXc) | Adults | Turkey | Mean: 29.7, range not reported | 76.3% female, 23.7% male | Not reported | Epilepsy Clinic of Haydarpaşa Numune Research and Training Hospital In Istanbul, Turkey |
| Zelikovsky & Lynn, 2002 [[118]](https://www.zotero.org/google-docs/?YbpeKX) | Adults | USA | Not reported | 49% male, 51% female | Not reported | Introductory college psychology courses |
| Zhou & Zhen, 2022 [[119]](https://www.zotero.org/google-docs/?CuCoWo) | Adolescents | China | Mean: 14.0, range: 12-17 | 46.9% male, 53.1% female | Not reported | Junior and senior high schools in Zhejiang Province, China |

**Note.** ^1^The exact language used in paper to describe gender, sex, race, and ethnicity is included in this table. Abbreviations in the order of appearance: USA= United States of America; UK=United Kingdom; LONGSCAN=Longitudinal Studies of Child Abuse and Neglect; IPV=Intimate Partner Violence; CPS=Child Protective Services.

**Table S5a. Design and Findings of Included Studies for Aims 1 and 2 - Children and Adolescents**

| **Study** | **Design** | **Sample** | **Childhood Adversity** | **Irritability** | **Main Findings** |
| --- | --- | --- | --- | --- | --- |
| Asgeirsdottir et al., 2011 [[16]](https://www.zotero.org/google-docs/?4ZlGnW) | Cross-sectional | 9,085 high schoolers in Iceland | Self-reported family conflict/violence and sexual abuse | Self-reported anger/hostility on SCL-90 | Structural Equation Modeling: Family conflict/violence and sexual abuse were significant predictors of increased anger for both female (family conflict: β=.28, p<.001, sexual abuse: β=.17, p<.001) and male participants (family conflict: β=.30, p<.001, sexual abuse: β=.13, p<.001). |
| Baron et al., 2019 [[36]](https://www.zotero.org/google-docs/?3aPed9) | Cross-sectional | 400 unhoused adolescents | Self-reported childhood maltreatment on CTQ | Self-reported anger | Correlation: Physical abuse (r=.14, p<.05), sexual abuse (r=.11, p<.05), and emotional neglect (r=.14, p<.05) were positively correlated with anger. Physical neglect was not significantly correlated with anger (r=.10, p<.10). |
| Benedini & Fagan, 2020 [[22]](https://www.zotero.org/google-docs/?HJqO9C) | Cross-sectional | 777 children and adolescents from LONGSCAN | Official record review; self- and parent-reported physical and sexual abuse on CTS | Self-reported TSCC Anger | Structural Equation Modeling: Physical abuse was a significant predictor of increased anger for girls only (β=.10, p<.05). Sexual abuse was a significant predictor of increased anger for both girls (β=.15, p<.01) and boys (β=.17, p<.01). |
| Bielas et al., 2016 [[40]](https://www.zotero.org/google-docs/?VWKEBz) | Cross-sectional | 130 male adolescents and young adults in a juvenile detention center | Self-reported ACEs on Multidimensional Clinical Screening Inventory | Self-reported irritability on the CIS | Correlation: ACEs were positively correlated with irritability (r=.19, p<.05). |
| Boonmann et al., 2016 [[12]](https://www.zotero.org/google-docs/?BgB7XK) | Cross-sectional | 88 male adolescents in a juvenile detention center | Self-reported childhood maltreatment on CTQ | Self-reported anger-irritability on MAYSI-2 | Correlation: Sexual abuse was positively correlated with anger-irritability (r=.58, p<.01) for juvenile sex offenders, but not non-sex offenders (r=-.09, p>.01). Emotional abuse, physical abuse, emotional neglect, and physical neglect were not significantly correlated with anger-irritability for sex offenders (r=.10, r=-.06, r=.08, r=.19, respectively, all ps>.01) and non-sex offenders (r=.13, r=.03, r=.22, r=.18, respectively, all ps>.01). |
| Brenner et al., 2018 [[42]](https://www.zotero.org/google-docs/?JtNksT) | Cross-sectional | 350 children and adolescents from specialty psychiatry hospitals as part of AIC | Parent-reported childhood abuse history on the Demographic and Medical Intake Form | Parent-reported irritability on ABC-C | Individuals with childhood abuse history (vs. without) reported increased irritability (t=−2.97, p=.003). |
| Brezina, 1998 [[43]](https://www.zotero.org/google-docs/?g5JbjF) | Cross-sectional | 1,502 male high schoolers | Self-reported childhood maltreatment | Self-reported anger | Structural Equation Modeling: Childhood maltreatment was a significant predictor of increased anger (β=.22, p<.01). |
| Briere et al., 2001 [[13]](https://www.zotero.org/google-docs/?qAn8Nf) | Cross-sectional | 104 children in community | Caregiver-reported sexual abuse, physical abuse, and witnessing domestic violence per clinical interview | Self-reported TSCYC Anger | Hierarchical Multiple Regression: Sexual abuse, physical abuse, and witnessing domestic violence were not significant predictors of anger. |
| Cazala et al., 2019 [[23]](https://www.zotero.org/google-docs/?wkkpJf) | Cross-sectional | 59 children and adolescents from BPD clinics | Self-reported childhood maltreatment on CTQ | Self-reported irritability on ARI | ANCOVA: Sexual abuse (F(1)=8.94; p<.01; η^2^_partial_=.19) and physical abuse (F(1)=6.23; p<.05; η^2^_partial_=.14) were significant predictors of increased irritability.  There were no main effects for emotional abuse, emotional neglect, or physical neglect. However, there was a significant gender by trauma interaction for these trauma types, such that ARI scores were higher in males with exposure than those without (emotional abuse: (F(1)=4.30; p<.05; η^2^_partial_=.10), emotional neglect: (F(1)=9.88; p<.01; η^2^_partial_=.20), and physical neglect: (F(1)=5.73; p<.05; η^2^_partial_=.13). |
| Cecil et al., 2014 [[46]](https://www.zotero.org/google-docs/?OVGIHM) | Cross-sectional | 189 inner city adolescents and young adults from community and secondary schools in UK | Self-reported childhood maltreatment on CTQ-SF and community violence exposure on CREV | Self-reported TSCC Anger | Multivariate Regression: Child maltreatment (β = 0.26, p <.05) and community violence (β = 0.28, p <.05) exposure were significant predictors of increased anger. |
| Coe et al., 2020 [[17]](https://www.zotero.org/google-docs/?tXOr2C) | Cross-sectional | 274 children from pediatric medical clinics and child protection agency | Staff-coded childhood maltreatment per CPS records | Caregiver-reported TBAQ Anger | Correlation & Path Analysis: Childhood maltreatment was not significantly correlated with, nor predicted, anger for both girls (r=.02, p>.10) and boys (r=.10, p>.10). |
| Collin-Vézina et al., 2011 [[49]](https://www.zotero.org/google-docs/?E1RRej) | Cross-sectional | 53 adolescents from residential care | Self-reported childhood maltreatment on CTQ | Self-reported TSCC Anger | One-way ANOVA and Chi-Square Test:  Clinical levels of anger were significantly associated with emotional abuse (t(1, 52)=10.13, *p*<.01), physical abuse (t(1, 52)=23.63, p<.001), sexual abuse (t(1, 52)=4.52, *p*<.05) emotional neglect (t(1, 52)=26.42, *p*<.001), and physical neglect (t(1, 52)=13.77, *p*<.001). Individuals with experiences of multiple types (vs. single type) of childhood maltreatment reported increased anger (X^2^ (5, N=53)=13.38, p<.01). |
| Cooley & Taussig, 2022 [[50]](https://www.zotero.org/google-docs/?68VQfZ) | Cross-sectional | 457 children with a history of out-of-home care | Staff-coded childhood maltreatment per CPS and court records;  Self-reported witnessing violence on TIHSH | Self-reported TSCC Anger | Correlation and Path Analysis: Witnessed violence was positively correlated with (r=.45, p<.05), and significantly predicted, anger (β=−.43, p<.05). Physical, emotional, or sexual abuse was not significantly correlated with anger (r=.05, r=.04, r=.00, respectively, all ps>.05). Physical or emotional neglect was not significantly correlated with anger (r=.01, r=-.01, respectively, all ps>.05). |
| Cotter et al., 2024 [[51]](https://www.zotero.org/google-docs/?vbe7AI) | Cross-sectional | 603 children with fathers with an IPV history | Caregiver-reported STRESS-C | Caregiver-reported temper loss on SDQ | T test & Network Analysis: Children with elevated irritability reported experiencing more trauma events (t=4.66, p<.001), emotional abuse (t=2.67, p=.003), and witnessing interpersonal violence (t=3.73, p<.001), but not sexual violence or loss of a loved one, per caregiver report.  In the network analysis, irritability was most strongly connected with PTSD symptom severity and parental psychopathology and stress, rather than experiences of child adversity. |
| Derella et al., 2020 [[52]](https://www.zotero.org/google-docs/?0CdnLw) | Longitudinal   **Time-lagged panel through age 16* | 2,450 girls in Pittsburgh neighborhoods | Parent- and child-reported parent-to-child aggression on CTSPC | Parent-reported CSI-4 Irritability | Regression: Only parent-reported (and not child-reported) corporal punishment significantly predicted increased irritability (IRR=1.05, p<.001). Both parent- (IRR=1.03, p<.001) and child-reported verbal aggression (IRR=1.01, p<.001) significantly predicted increased irritability. |
| Evans et al., 2012 [[2]](https://www.zotero.org/google-docs/?NklPZW) | Cross-sectional | 704 African American children and adolescents in community | Self-reported experiences of corporal punishment and verbal abuse | Self-reported enduring feelings of anger and frustration on DISC-IV | Correlation & Structural Equation Modeling: Verbal abuse was positively correlated with anger for both girls (r=.31, p<.001) and boys (r=.11, p<.05). Verbal abuse predicted anger in girls (β=.28, p<.01), but not boys (β=.11, p<.10). Corporal punishment was not significantly correlated with (r=−.01, p>.05, full sample), nor predicted, anger for both girls and boys. |
| Faulkner et al., 2014 [[55]](https://www.zotero.org/google-docs/?ixfhVD) | Longitudinal | 158 adolescents and young adults in community | Self-reported childhood maltreatment on CTQ Short Form; Self-reported witnessed IPV on CEVQ | Self-reported TSCC Anger (assessed 1.5 years later) | Correlation & Regression: Childhood maltreatment (r=-.25, p<.05) and witnessing IPV (r=.32, p<.05) were positively correlated with, and significantly predicted, anger (maltreatment: b=.06, p=.029; witnessing IPV: b=.73, p<.001). |
| Feiring et al., 2013 [[56]](https://www.zotero.org/google-docs/?6UD1tt) | Prospective | 121 children and adolescents from CPS offices and child abuse clinics | Abuse severity coded based on CPS records | Sele-reported TSI Anger (assessed 6 years later) | Correlation: Abuse severity was not significantly correlated with anger/irritability (r=.09, p>.05). |
| Fitzgerald, 2021 [[57]](https://www.zotero.org/google-docs/?O0SPfy) | Longitudinal | 313 adolescents from LONGSCAN | Self-reported physical, sexual, and emotional abuse (assessed at age 12) | Self-reported TSC Anger (assessed at age 16) | Correlation & Structural Equation Modeling: Physical abuse was positively correlated with , and significantly predicted, anger (r=.23, p<.001; β=.21, *p*<.05).  Multiple types of sexual abuse were positively correlated with, and significantly predicted, anger (rs=.13-.28, p<.001; β=.24, *p*<.01). |
| Grasser et al., 2024 [[64]](https://www.zotero.org/google-docs/?LyQeug) | cross-sectional | 48 treatment-seeking children and adolescents | Self-reported childhood abuse and neglect CTQ | Self-reported irritability on ARI | Regression: Childhood abuse (β=.35, p=.01), not neglect (β=.35, p=.07), was a significant predictor of increased irritability. |
| Gunnlaugsson et al., 2011 [[65]](https://www.zotero.org/google-docs/?7tkxVp) | Cross-sectional | 3515 students in 9th and 10th grades in Iceland | Self-reported witnessed or experienced verbal and physical violence | Self-reported anger severity | T test: Adolescents who witnessed physical and verbal violence reported increased anger relative to those who had not witnessed violence. (d = 0.74 and 0.39, respectively)  Adolescents who experienced physical and verbal violence reported increased anger relative to those who had not experienced violence (d = 0.89 and 0.52, respectively). |
| Hamstra & Fitzgerald, 2022 [[66]](https://www.zotero.org/google-docs/?tOZ86w) | Cross-sectional | 239 adolescents from LONGSCAN | Self-reported physical, sexual, and emotional abuse | Self-reported TSC Anger | Correlation & Structural Equation Modeling: Childhood abuse was positively correlated with and significantly predicted anger (r=.34, p<.001; β=.43, p<.001). |
| Henriksen et al., 2021 [[18]](https://www.zotero.org/google-docs/?BmLBmv) | Cross-sectional | 7422 adolescents in community in Norway | Self-reported trauma exposure on UCLA PTSD Reaction Index | Self-reported irritability on SDQ | Logistic Regression: Trauma exposure was a significant predictor of increased irritability for both girls and boys (total sample: OR=1.17, p<.001; boys only: OR=1.13, p<.001; girls only: OR=1.21, p<.001). |
| Johnsona et al., 2002 [[70]](https://www.zotero.org/google-docs/?oGkMXx) | Cross-sectional | 167 mother-child dyads from LONGSCAN | Official record review; mother-reported child victimization on CTS and Child Life Events; child self-reported "Things I've Seen and Heard" | Child self-reported TSCC Anger | ANOVA: Children with moderate or severe self-reported witnessed violence (vs. none), but not caregiver-reported witnessed violence (p=.448) or child victimization status (p=.091), had significantly higher anger (least-squares means for severe: 7.98, moderate: 6.01, none: 3.67, p=.002). |
| Kobulsky et al., 2018 [[19]](https://www.zotero.org/google-docs/?Vo0iou) | Longitudinal | 1161 children and adolescents from LONGSCAN | Self-reported childhood victimization; CPS record review (assessed at age 12) | Self-reported TSCC-A Anger (assessed at age 16) | Structural Equation Modeling: Childhood abuse and neglect significantly predicted anger for both girls (abuse: β=.15, p<.05; neglect: β=.30, p<.001) and boys (abuse: β=.17, p<.01; neglect: β=.30, p<.001). |
| Leban & Delacruz, 2023 [[24]](https://www.zotero.org/google-docs/?ZsGqlo) | Longitudinal | 979 children and adolescents from LONGSCAN | CPS record reviews; caregiver-reported household dysfunction ACEs (e.g., parental IPV) (assessed at age 12) | Self-reported TSC Anger (assessed at age 16) | Binomial Regression: Household dysfunction ACEs was a significant predictor of increased anger for girls (IRR=1.07, p<.01), but not boys (IRR=1.05, p>.05). |
| Li et al., 2025 [[73]](https://www.zotero.org/google-docs/?BwdbS2) | Longitudinal | 4581 adolescents in a juvenile probation center | Self-reported ACEs | Self-reported persistent anger/irritability (assessed 1 year later) | Network Analysis: Anger was positively connected with all ACEs except for parental separation/divorce. Anger was more strongly connected with physical abuse, emotional abuse, and physical neglect, compared with other ACEs. |
| Litrownik et al., 2005 [[74]](https://www.zotero.org/google-docs/?JXuKi3) | Cross-sectional | 519 children and adolescents and LONGSCAN | CPS record review | Self-reported TSCC Anger | Hierarchical Regression: Physical abuse at maximum severity was a significant predictor of increased anger (b=2.94, p<.05). |
| Malvaso et al., 2017 [[76]](https://www.zotero.org/google-docs/?WOXj4l) | Cross-sectional | 28 male adolescents in a juvenile detention center | Self-reported childhood trauma on the Childhood Trauma Questionnaire (Pennebaker & Susman, 1988)* | Self-report anger on SAM | Correlation & Chi-Square Test: Childhood trauma was not significantly correlated with anger (r=.05, p>.05). Adolescents with one major trauma were more likely to score above the 75th percentile on the anger measure (X^2^=5.08, p<.05). |
| Marie-Mitchell et al., 2020 [[77]](https://www.zotero.org/google-docs/?x5zRM2) | Cross-sectional | 499 children from clinics | Caregiver-reported ACEs | Caregiver-reported anger on WCA | Logistic Regression: Having 2 or more ACEs was a significant predictor of increased anger (Adjusted OR=9.57, p≤.05) |
| Mills et al., 2020 [[78]](https://www.zotero.org/google-docs/?VyQpiw) | Cross-sectional | 106 children from the community | Self-reported childhood trauma on the UCLA Trauma History Profile | Self-reported TSCC Anger | Correlation: The number of childhood trauma events was positively correlated with anger (r=.34, p<.05). |
| Papalia et al., 2022 [[85]](https://www.zotero.org/google-docs/?b0oKQq) | Cross-sectional | 165 adolescents from Youth Justice Centers in Australia | Self-reported childhood maltreatment on CTQ | Self-reported BDHI Irritability | ANOVA: Adolescents with poly-victimization (vs. low/rare maltreatment) reported increased irritability (p=.001, d=.58). |
| Peng et al., 2022 [[86]](https://www.zotero.org/google-docs/?GfX1cZ) | Cross-sectional | 16111 high schoolers in China | Self-reported parent-to-child aggression on CTSPC | Self-reported BWAQ Anger | Structural Equation Modeling: Childhood abuse was a significant predictor of increased anger (β=.15, p<.05). |
| Plattner et al., 2007 [[89]](https://www.zotero.org/google-docs/?1swZDh) | Cross-sectional | 56 post-adjudicated adolescents | Self-reported childhood trauma on CTI | Self-reported WAI-M Trait Anger | Correlation: The number or severity of multiple types of childhood trauma were not significantly correlated with trait anger (rs=-.17-.16, all ps>.05). |
| Qirjako et al., 2021 [[90]](https://www.zotero.org/google-docs/?X9aJH2) | Cross-sectional | 1,541 adolescents in Albania | Self-reported physical abuse | Self-reported irritability frequency | Logistic Regression: Physical abuse was a significant predictor of increased irritability (OR=2.0, p<.001). |
| Renner et al., 2020 [[93]](https://www.zotero.org/google-docs/?TDiVOR) | Cross-sectional | 1,079 children and adolescents in community | Self-reported childhood maltreatment, sexual assault, and witnessing family violence on JVQ | Self-reported TSCC Anger | Logistic Regression: Physical abuse (OR=4.16), emotional abuse (OR=4.28), sibling aggression (OR=2.29), bullying (OR=2.64), witnessing parental IPV (OR=2.82), parental assault of a sibling (OR=4.56) were all significant predictors of clinically significant anger. Sexual assault (OR=.79) and neglect (OR=1.59) were not significant predictors of anger. |
| Rinne-Albers et al., 2016 [[30]](https://www.zotero.org/google-docs/?Fa26HV) | Cross-sectional | 40 adolescents from specialized psychotrauma centers (20 with CSA and PTSD; 20 healthy controls) | PTSD diagnosis with CSA history | Self-reported TSCC Anger | Mann-Whitney U-test: The PTSD/CSA group reported significantly higher anger relative to the healthy control group (U=2.0, p<.001). |
| Rizzo et al., 2014 [[20]](https://www.zotero.org/google-docs/?NJKEfL) | Cross-sectional | 155 adolescents from an inpatient unit of a psychiatric hospital | Self-reported childhood abuse on K-SADS-PL | Self-reported AQ Anger | Correlation: Childhood abuse was not significantly correlated with anger for both girls (r=.11, p>.05) and boys (r=.07, p>.05). |
| Roe-Sepowitz & Krysik, 2008 [[94]](https://www.zotero.org/google-docs/?xob2El) | Cross-sectional | 118 female juvenile sex offenders | Self-reported childhood maltreatment on MYSI-2 and SRCI | Self-reported MYSI-2 Angry-Irritable Scale | Chi-Square Test: Participants with maltreatment history were more likely to report elevated anger/irritability (X^2^(1, N=118)= 6.71, p<.01). |
| Schneider et al., 2005 [[95]](https://www.zotero.org/google-docs/?DlUsjh) | Cross-sectional | 806 children from LONGSCAN | Official record review | Self-reported TSCC Anger | Hierarchical Regression: Emotional abuse (safety-restriction type, but not self-esteem and autonomy type) was a significant predictor of increased anger (β=2.79, p<.05). Overall maltreatment that was not emotional was also a significant predictor of increased anger (β=2.50, p<.05). |
| Sebre et al., 2004 [[96]](https://www.zotero.org/google-docs/?ysQd4I) | Cross-sectional | 1,145 children and adolescents in primary schools of Eastern Europe | Self-reported childhood maltreatment on CTS | Self -reported TSCC Anger | Correlation: Childhood maltreatment (abuse and neglect) was positively correlated with anger (correlations reported by country, Latvia: r=.54, Lithuania: r=.46, Macedonia: r=.56, Moldova: r=.47, all ps<.01). |
| Sevenoaks et al., 2022 [[97]](https://www.zotero.org/google-docs/?zz94a0) | Cross-sectional | 135 adolescents (99 with perinatal HIV and 36 healthy controls) in South Africa | Self-reported childhood maltreatment on CTQ | Self-reported anger on BYI-2 | Correlation: Emotional abuse, but not other types of childhood maltreatment (physical and sexual abuse, physical and emotional neglect, rs= .01-.17, all ps>.05), was positively correlated with anger (r=.32, p<.001). |
| Showalter et al., 2020 [[98]](https://www.zotero.org/google-docs/?fWXI5K) | Cross-sectional | 580 children from a mental health outpatient unit | Caregiver-reported childhood maltreatment based on medical forensic interview | Caregiver-reported TSCYC Anger | Logistic Regression: Physical abuse only, but not exposure to IPV only (OR=1.42, p>.05) was a significant predictor of elevated anger (OR=1.65, p<.05). Combined physical abuse and exposure to IPV was a significant predictor of elevated anger (OR=3.33, p<.001). |
| Sigfusdottir et al., 2013 [[21]](https://www.zotero.org/google-docs/?WLxOPH) | Cross-sectional | 9,085 high schoolers in Iceland | Self-reported family violence and sexual abuse | Self-reported SCL-90 Anger/Hostility Scale | Correlation & Structural Equation Modeling: Sexual abuse was positively correlated with (males: r=.15, p<.001; females: r=.22, p<.001) and significantly predicted anger for both female and male participants (males: β=.13, p<.001; females: β=.17, p<.001).  Family violence was positively correlated with (males: r=.30, p<.001; females: r=.27, p<.001) and significantly predicted anger for both female and male participants (males: β=.30, p<.001; females: β=.28, p<.001). |
| Solakoglu et al., 2018 [[100]](https://www.zotero.org/google-docs/?jXTA3i) | Cross-sectional | 2,067 adolescents in Turkey | Self-reported sexual abuse | Self-report anger | Regression: Sexual abuse was a significant predictor of increased anger (β=.07, p<.001). |
| Tang et al., 2017 [[102]](https://www.zotero.org/google-docs/?PMrAeN) | Cross-sectional | 1,082 male adolescents and young adults in a youth correctional facility | Self-reported childhood maltreatment on CTQ | Self-reported anger on STAXI-2 | Correlation: Emotional (r=.32), physical (r=.25), and sexual abuse (r=.23) and physical neglect (r=.08) were positively correlated with anger (all ps<.01) Emotional neglect was not correlated with anger (r=.05, p>.01). |
| Turner et al., 2006 [[106]](https://www.zotero.org/google-docs/?j7HqAj) | Cross-sectional | 993 children and adolescents in community, with 1000 caregivers of children ages 2-9 | Self-reported childhood victimization on JVQ | Self-reported TSCC Anger (ages 10-17); Caregiver-reported TSCYC-Anger (ages 2-9) | Hierarchical Regression: Childhood maltreatment (2-9 year olds: β=.22, p<.001; 10-17 year olds: β=.12, p<.001)and non-victimization adversity (2-9 year olds: β=.21, p<.001; 10-17 year olds: β=.29, p<.001) were significant predictors of increased anger, regardless of age. Witnessing family violence was a significant predictor for 2-9 year olds (β=.12, p<.001) but not 10-17 year olds (β=-.01, p>.05). |
| van Vugt et al., 2014 [[107]](https://www.zotero.org/google-docs/?5cgRnw) | Cross-sectional | 89 female adolescents in residential care | Self-reported maltreatment on CTQ | Self-reported TSCC Anger | Hierarchical Regression: Emotional abuse (β=.37, p<.05) and emotional neglect (β=.33, p<.05) were significant predictors of increased anger. Physical neglect (β=-.27, p>.05) and physical (β=-.02, p<.05) and sexual abuse (β=.01, p>.05) were not significant predictors. |
| Wang et al., 2020 [[111]](https://www.zotero.org/google-docs/?JzerQT) | Longitudinal | 435 middle schoolers in China | Self-reported maltreatment on CTQ | self-report STAXI Trait Anger (assessed 6 months later) | Correlation: Childhood maltreatment was positively correlated with trait anger (r=.27, p<.001). |
| White et al., 2016 [[112]](https://www.zotero.org/google-docs/?3eqyk1) | Longitudinal | 770 adolescents from LONGSCAN | Official record review; self-reported emotional maltreatment (assessed at age 12 and 16) | Self-reported TSI Anger (assessed at age 18) | Regression: Emotional maltreatment, not physical abuse (b=.16, p<.05), sexual abuse (b=.18, p>.05), or neglect (b=.54, p<.05), was a significant predictor of increased anger.  Among emotional maltreatment dimensions, only threats to psychological safety was a significant predictor of increased anger (b=.60, p<.05). |
| Wolfe et al., 2001 [[25]](https://www.zotero.org/google-docs/?VSihKD) | Cross-sectional | 1,419 high schoolers in Canada | Self-reported maltreatment on CTQ | Self-reported TSCC Anger | Logistic Regression: Girls, not boys, with moderate/severe maltreatment (vs. not maltreated) reported increased anger (girls: OR=7.1, p<.01; boys: OR=3.3, p>.01). |
| Xu et al., 2024 [[115]](https://www.zotero.org/google-docs/?9AxnRq) | Cross-sectional | 5,724 middle schoolers in China | Self-reported maltreatment on CTQ | Self-reported irritability on CIS | Correlation & Structural Equation Modeling: Childhood maltreatment was positively correlated with (males: r=.29, p<.01; females: r=.31, p<.01), and a significant predictor of increased irritability (β=.24, p<.01). |
| Zhou & Zhen, 2022 [[119]](https://www.zotero.org/google-docs/?tUvGVq) | Longitudinal | 1,689 high schoolers in China | Self-reported emotional and physical abuse on CTQ | Self-reported trait anger | Correlation: Emotional abuse (r=.21, p<.001) and physical abuse (r=.11, p<.001) were positively correlated with trait anger. |

**Note.** Statistics, including nonsignificant findings, are included alongside descriptive results when reported. β refers to standardized regression coefficients and b refers to unstandardized regression coefficients, as reported. ANCOVA=Analysis of Covariance; ANOVA=Analysis of Variance; IRR=Incidence rate ratio; OR=odds ratio.

Abbreviations of measures in the order of appearance: SCL-90=Symptom Checklist-90; CTQ=Childhood Trauma Questionnaire; CTS=Conflict Tactics Scale; TSCC=Trauma Symptom Checklist for Children; CIS=Caprara Irritability Scale; MAYSI-2=Massachusetts Youth Screening Instrument-2; ABC-C=Aberrant Behavior Checklist; TSCYC=Trauma Symptom Checklist for Young Children; ARI=Affective Reactivity Index; CTQ-SF=Childhood Trauma Questionnaire-Short Form; CREV=Children’s Report of Exposure to Violence; CPS=child protection services; TBAQ=Toddler Behavior Assessment Questionnaire; TIHSH=Things I Have Seen and Heard; STRESS-C=Structured Trauma-Related Experiences and Symptoms Screener-Child Edition; SDQ=Strength and Difficulties Questionnaire; CTSPC=Conflict Tactics Scale-Parent/Child; CSI-4=Child Symptom Inventory-4; DISC-IV=Diagnostic Interview Schedule for Children, Version IV; CEVQ=Childhood Experiences of Victimization Questionnaire; TSI=Trauma Symptom Inventory; TSC=Trauma Symptom Checklist; SAM=Short Anger Measure; WCA=Whole Child Assessment; BDHI=Buss-Durkee Hostility Inventory; BWAQ=Buss and Warren’s Aggression Questionnaire; CTI=Childhood Trauma Interview; WAI-M=Weinberger Adjustment Inventory-Modified; JVQ=Juvenile Victimization Questionnaire; K-SADS-PL=Kiddie Schedule for Affective Disorders and Schizophrenia, Present and Lifetime Version; AQ=Aggression Questionnaire; SRCI=Supervision Risk Classification Instrument; BYI-2=Beck Youth Anger Inventory-2; STAXI-2=State-Trait Anger Expression Inventory-2.

**Table 5b. Design and Findings of Included Studies for Aims 1 and 2 – Adults**

| **Study** | **Design** | **Sample** | **Childhood Adversity** | **Irritability** | **Main Findings** |
| --- | --- | --- | --- | --- | --- |
| Adler et al., 2020 [[35]](https://www.zotero.org/google-docs/?2E5uCv) | Longitudinal | 90,266 adults from US military rosters | Self-reported childhood victimization on JVQ | Self-reported anger on DAR-5 (assessed 3-4 years later) | Logistic Regression: Having 3 or more childhood victimization experiences (vs. none) was a significant predictor of increased anger (OR=2.70; 95% CI: 2.43, 3.00). |
| Almeida et al., 2024 [[27]](https://www.zotero.org/google-docs/?a1E5PH) | Cross-sectional | 1,541 adults from community | Self-reported childhood adversity on CHQ | Self-reported BPAQ-SF Anger | Correlation: Childhood adversity was positively correlated with anger in both women (r=.22, p<.001) and men (r=.14, p<.01). |
| Barrett et al., 2013 [[37]](https://www.zotero.org/google-docs/?QQSHXM) | Cross-sectional | 58 adults from an inpatient clinic | Self-reported childhood trauma on CIDI | Self-reported anger severity | Regression: Childhood trauma was a significant predictor of increased anger (b=1.81, p<.05). |
| Beesley et al., 2010 [[38]](https://www.zotero.org/google-docs/?odZhn6) | Cross-sectional | 151 adults from outpatient clinics (75 with IBS, 76 with Crohn's disease) | Self-reported physical, sexual, and emotional abuse per medical history | Self-reported trait anger on STAXI-2 | Univariate ANOVA: Childhood sexual abuse was not a significant predictor of trait anger (p=.09). |
| Berthelot et al., 2014 [[39]](https://www.zotero.org/google-docs/?9amlji) | Cross-sectional | 114 adults from outpatient clinics | self-reported childhood abuse and neglect on ETI-SR and CCMS | Self-reported trait anger on STAXI | Correlation: Childhood maltreatment was positively correlated with trait anger (r=.23, p≤.01). |
| Bilgi et al., 2017 [[31]](https://www.zotero.org/google-docs/?YtHR9C) | Cross-sectional | 72 adults from an inpatient unit (36 diagnosed with schizophrenia, 36 healthy control) | Self-reported childhood maltreatment on CTQ | Self- and caregiver-reported irritability on IRQ | Regression: Childhood maltreatment was a significant predictor of increased caregiver-reported irritability for schizophrenia patients (β=.34, p=.04). |
| Bock et al., 2020 [[41]](https://www.zotero.org/google-docs/?Er3diU) | Cross-sectional | 325 adults from community in Brazil | Self-reported childhood maltreatment on CTQ | Self-reported anger on AFECTS | One-way ANOVA: Individuals with mild/moderate or severe childhood ,maltreatment had significantly higher levels of anger (p<.001). |
| Carver et al., 2014 [[44]](https://www.zotero.org/google-docs/?o6tY9I) | Cross-sectional | 369 undergraduates | Self-reported risky family environment on RF | Self-reported AQ-SF Anger | Correlation: Risky family environment was positively correlated with anger (r=.34, p<.001). |
| Cascardi et al., 2020 [[45]](https://www.zotero.org/google-docs/?p3fHs9) | Cross-sectional | 504 university students | Self-reported physical and emotional abuse on EASE-PI and witnessing family violence on JVQ | Self-reported AQ Anger | Correlation: Physical abuse (r=.23, p<.001), emotional abuse (r=.25, p<.001), and witnessing family violence (r=.19, p<.001) were positively correlated with anger. |
| Choi et al., 2009 [[47]](https://www.zotero.org/google-docs/?SsRafE) | Cross-sectional | 33 from an inpatient psychiatric unit | Medical chart review | Nurse-reported irritability on NOSIE-30 | Correlation & Regression: Sexual abuse was positively correlated with (r=.46, p<.01) and significantly predicted increased irritability (β=.45, p<.05). |
| Chong et al., 2023 [[48]](https://www.zotero.org/google-docs/?Yt5P7p) | Cross-sectional | 182 undergraduates | Self-reported parent-to-child psychological aggression on CTSPC | Self-reported AQ Anger | Correlation & Regression: Psychological aggression was positively correlated with (r=.29, p≤.01) and significantly predicted anger (β=.47, p<.01). |
| de Bles et al., 2023 [[34]](https://www.zotero.org/google-docs/?WxnFoA) | Longitudinal | 2,271 adults in Netherlands (1199 with anxiety/depression, 1072 healthy controls) | Self-reported childhood trauma on CTI (assessed at baseline) and CTQ-SF | Self-report anger on STAXI-2 (assessed 4 years later) | ANOVA: Higher childhood trauma was associated with greater anger F(1,2270) = 60.64, p<.001). All types of childhood trauma, expect sexual abuse, were independently associated with trait anger (p<.001). |
| DiLillo et al., 2000 [[53]](https://www.zotero.org/google-docs/?nuTAvQ) | Cross-sectional | 290 women from local Women, Infants, and Children (WIC) clinics | Self-reported childhood physical and sexual abuse by interview | Self-reported BSI Hostility | ANOVA: Participants with childhood sexual abuse had significantly higher anger than those without childhood sexual abuse (F(1,286)=16.2, p=.001). |
| Etchin et al., 2022 [[54]](https://www.zotero.org/google-docs/?VCNwin) | Cross-sectional | 167 veterans | Self-reported childhood maltreatment on CTQ | Self-report anger on DAR-5 | Correlation: Childhood maltreatment was positively correlated with anger (r=.35, p≤.001). |
| Fitzgerald & Williams, 2024 [[58]](https://www.zotero.org/google-docs/?x3RysQ) | Cross-sectional | 596 adults from community | Self-reported childhood maltreatment on CTQ | Self-reported trait anger on STAXI | Correlation: Childhood maltreatment was positively correlated with trait anger (r=.21, p<.001) |
| Fuchshuber et al., 2019 [[59]](https://www.zotero.org/google-docs/?boGNY2) | Cross-sectional | 616 adults in Austria | Self-reported childhood maltreatment on CTQ | Self-reported ANPS Anger | Correlation: Childhood maltreatment was positively correlated with anger (r=.20, p<.001). |
| Gardner et al., 2014 [[60]](https://www.zotero.org/google-docs/?0NXSix) | Cross-sectional | 88 adult violent (IPV and non-IPV) offenders mandated for mental health treatment | Self-reported childhood maltreatment on CTQ | Self-reported trait anger on STAXI-2 | Correlation: Childhood maltreatment, including its 5 CTQ subtypes, was not significantly correlated with trait anger (all ps >.05). |
| Gause et al., 2022 [[61]](https://www.zotero.org/google-docs/?7MvyWz) | Cross-sectional | 440 adults eligible for an HIV-risk reduction intervention trial | Self-reported physical, sexual, and emotional abuse | Self-reported trait anger on STAS | Correlation: Childhood abuse was positively correlated with trait anger (r=.35, p<.001). |
| Gerhant & Olajossy, 2016 [[62]](https://www.zotero.org/google-docs/?11MXyx) | Cross-sectional | 90 adults in treatment for alcohol use problems | Self-reported childhood trauma on ETI-SR | Self-reported BPAQ Anger | Mann-Whitney U Test: Participants with high (vs. low) childhood abuse reported increased anger (Z=2.05, p=.04). |
| Glück et al., 2017 [[63]](https://www.zotero.org/google-docs/?Ycih5T) | Cross-sectional | 220 adult survivors of institutional abuse | Self-reported childhood maltreatment on CTQ | Self-reported trait anger on STAXI | Network Analysis: There were no significant paths between any type of childhood maltreatment and trait anger. |
| Harper & Arias, 2004 [[26]](https://www.zotero.org/google-docs/?ogyss6) | Cross-sectional | 294 undergraduates | Self-report childhood psychological maltreatment on PMI | Self-reported anger on MAI | Correlation: Psychological abuse was positively correlated with anger (r=.26, p<.001). |
| Hasler et al., 2012 [[67]](https://www.zotero.org/google-docs/?g0O4Zw) | cross-sectional | 288 adults (265 who had a previous suicide attempt, 23 controls without a previous attempt) | Self-reported childhood maltreatment on CTQ | Self-reported anger on STAXI | Chi-Square Test: Participants with sexual abuse (vs. no sexual abuse) reported increased anger. (X^2^(1, N=434)= -5.7, p<.001). |
| Herrenkohl et al., 2012 [[68]](https://www.zotero.org/google-docs/?51BvDD) | Prospective | 357 adults once in welfare system in Pennsylvania | Childhood maltreatment per official record review; caregiver-reported abusive disciplining | Self-reported anger proneness (assessed 34-35 years later) | Correlation & Hierarchical Regression: Childhood maltreatment (r=.17, p<.01) and abusive disciplining (r=.21, p<.01) were positively correlated with, and significantly predicted (maltreatment: β=.13, p<.05; discipling: β=.18, p<.01), adulthood anger proneness. |
| Hobfoll et al., 2002 [[69]](https://www.zotero.org/google-docs/?DlMNk0) | Cross-sectional | 160 Native American women | Self-reported childhood maltreatment on CTQ | Self-reported anger on STAXI-2 | Correlation & Hierarchical Regression: Physical-emotional abuse (r=.34, p<.001), not sexual abuse (r=.09, p>.05), was positively correlated with and significantly predicted anger (physical: β=.31, p<.001; sexual: β=.01, p>.05). |
| Keene & Epps, 2016 [[71]](https://www.zotero.org/google-docs/?MaUJy6) | Cross-sectional | 400 undergraduates | Self-reported physical and emotional abuse on EPAB | Self-reported trait anger on AQ | MANCOVA: Individuals with a history of childhood abuse had significantly higher trait anger than those without an abuse history (F(1,363)=12.07, p<.006). |
| Kendra et al., 2012 [[72]](https://www.zotero.org/google-docs/?rxvnAe) | Cross-sectional | 496 female undergraduates | Self-reported childhood physical and sexual abuse on CTS and FSCSE | Self-reported anger arousal on MAI | Correlation: Childhood abuse was positively correlated with anger arousal (r=.14, p<.01). |
| Macarenco et al., 2021 [[75]](https://www.zotero.org/google-docs/?Phr5JF) | Cross-sectional | 598 adults in Romania | Self-reported childhood maltreatment on MAES | Self-reported trait anger on STAXI-2 | Path Analysis: Childhood maltreatment was a significant predictor of increased trait anger (z=7.18, p<.01). |
| Moore et al., 2020 [[79]](https://www.zotero.org/google-docs/?IYYMts) | Cross-sectional | 369 undergraduates | Self-reported childhood maltreatment on CTQ | Self-reported AQ Anger | Correlation: Childhood maltreatment (r=.20, p<.01), emotional abuse (r=.23, p<.001), emotional neglect (r=.22, p<.05), and physical neglect (r=.11, p<.05) were positively correlated with anger; sexual (r=.04, p>.05) and physical abuse (r=.05, p>.05) were not significantly correlated with anger.  Independent t test: Participants with childhood maltreatment history reported increased anger (t=3.04, p<.01). |
| Morandotti et al., 2013 [[32]](https://www.zotero.org/google-docs/?aJpjLG) | Cross-sectional | 37 adults (18 diagnosed with BPD and 19 healthy controls) | Clinician-reported childhood abuse on CAS | Self-reported BDHI Irritability | Correlation: Abuse severity was positively correlated with irritability (r=.69, p=.03) in the subsample of BPD patients with childhood abuse history.  Independent t test: Participants (with BPD) with childhood abuse history (vs. without) reported increased irritability (t=3.1, p=.006). |
| Murphy & Turgoose, 2022 [[80]](https://www.zotero.org/google-docs/?w3qhBs) | Cross-sectional | 403 veterans in UK | Self-reported ACEs | Self-reported anger on DAR-5 | Logistic Regression: Having anger problems was a significant predictor of having 6+ ACEs (OR=2.17. p<.05). |
| Nakai et al., 2014 [[81]](https://www.zotero.org/google-docs/?j695oV) | Cross-sectional | 294 adults from community in Japan | Self-reported childhood abuse and trauma on CATS | Self-reported irritability on TEMPS-A | Correlation & Regression: Childhood neglect (r=.39, p<.01) and punishment (r=.20, p<.01), but not sexual abuse (r=.09), were positively correlated with irritability. Childhood neglect was a significant predictor of increased irritability (*β*=.04, p<.01), but not punishment (*β*=.03) or sexual abuse (*β*=-.05). |
| Novaco & Taylor, 2008 [[82]](https://www.zotero.org/google-docs/?8HhDrH) | Cross-sectional | 137 male adults receiving hospital forensic services | Self-reported childhood adversity by interview | Self-reported anger on STAXI (trait), NAS, PI; Staff-reported anger on WARS | Independent t test: Participants with childhood abuse history (vs. without) reported greater anger on NAS (p=.003, d=.59) and WARS (p=.032, d=.42), but not on trait anger or PI. |
| Osvath et al., 2004 [[83]](https://www.zotero.org/google-docs/?HXQf6O) | Cross-sectional | 101 adults from general and psychiatric hospitals in Hungary | Self-reported childhood trauma on STEQ | Self-reported trait anger on STAS | Correlation: Childhood trauma was positively correlated with trait anger (r=.35, p<.01). |
| Otsuka et al., 2017 [[84]](https://www.zotero.org/google-docs/?oNl1VR) | Cross-sectional | 415 adults from community in Japan | Self-reported childhood abuse and trauma on CATS | Self-reported irritability on TEMPS-A | Correlation: Childhood neglect (r=.32, p<.01) and punishment (r=.12, p<.05), not sexual abuse (r=.08, p>.05), were positively correlated with irritability. |
| Perroud et al., 2010 [[87]](https://www.zotero.org/google-docs/?VPSgjg) | Cross-sectional | 875 adults from psychiatric hospitals who had a previous suicide attempt | Self-reported childhood maltreatment on CTQ | Self-reported trait anger on STAXI | Regression: Emotional abuse (b=1.43, p=.001) and physical neglect (b=.82, p=.048), but not sexual abuse (b=.76, p=.083) or emotional neglect (b=.39, p=.484), were significant predictors of increased trait anger. |
| Peterson et al., 2018 [[88]](https://www.zotero.org/google-docs/?K0Zjmv) | Cross-sectional | 377 adult males | Self-reported childhood maltreatment (sexual abuse subscale) on CTQ | Self-reported trait anger on STAXI | Correlation & Regression: Childhood sexual abuse was positively correlated with (r=.30, p<.001) and significantly predicted increased trait anger (*β*=.33, p=.002). |
| Porcerelli et al., 2015 [[3]](https://www.zotero.org/google-docs/?IwJd9t) | Cross-sectional | 98 African American adult women from a primary care clinic | Self-reported childhood maltreatment on CTQ | Self-reported control of anger on PAS | Correlation & Regression: Anger control was not significantly correlated with childhood physical or sexual abuse (r=.16, .25, respectively). Anger control was not a significant predictor of childhood abuse (p>.05). |
| Quenneville et al., 2020 [[91]](https://www.zotero.org/google-docs/?18QdjC) | Cross-sectional | 388 outpatient adults with BPD | Self-reported childhood maltreatment on CTQ | Self-reported trait anger on STAXI | Regression: Childhood maltreatment was a significant predictor of increased trait anger (b=.07, p<.001). |
| Rapoza et al., 2014 [[92]](https://www.zotero.org/google-docs/?J78X8v) | Cross-sectional | 198 adults from a non-traditional college | Self-reported childhood maltreatment on CTS | Self-reported anger on shortened version of MAI | Correlation: Childhood maltreatment was positively correlated with anger (r=.15, p<.05). |
| Snyder et al., 2024 [[99]](https://www.zotero.org/google-docs/?QfD2sC) | Cross-sectional | 559 adult males in the US Air Force | Self-reported childhood adversity on ACE-Q | Self-reported anger on CAS* | Correlation: Emotional/physical neglect (r=.14, p<.01), emotional/physical abuse (r=.15, p<.01), and interparental physical abuse (r=.08, p<.05) were positively correlated with anger.  Risk Ratio: Participants with at least 1 ACE were more likely to report anger difficulties (risk ratio=2.32) |
| Springer et al., 2007 [[101]](https://www.zotero.org/google-docs/?WVdtWB) | Cross-sectional | 2051 adults (234 had experienced childhood abuse, 1817 were non-abused) | Self-reported childhood maltreatment on CTS | Self-reported trait anger on STAXI-2 | Independent t test: Individuals with abuse history reported greater anger symptoms (p<.01).  Regression: Physical abuse (b=.20, p<.01) was a significant predictor of trait anger. |
| Stearns & McKinney, 2020 [[28]](https://www.zotero.org/google-docs/?jlJuEf) | Cross-sectional | 1012 adults from a university | Self-reported parent-to-child psychological aggression on CTSPC | Self-reported irritability on ASR latent factor of irritability | Correlation: Maternal and paternal psychological and physical abuse were positively correlated with irritability (r=.26, .21, .16, .24, ps<.05) respectively).  Regression: Maternal psychological maltreatment was a significant predictor of irritability for both female (*β*=.15, p<.05) and male (*β*=.16, p<.05) participants, while paternal psychological maltreatment was a significant predictor of irritability for female (*β*=.13, p<.05) participants only.Maternal physical maltreatment was not a significant predictor of irritability for both female and male participants, while paternal physical maltreatment was a significant predictor of irritability for female (*β*=.12, p<.05) participants only. |
| Sudbrack et al., 2015 [[29]](https://www.zotero.org/google-docs/?mH8NFf) | Cross-sectional | 10,800 adults from community in Brazil | Self-reported childhood maltreatment on CTQ | self-reported anger on AFECTS | Regression: Emotional abuse was a significant predictor of increased anger in males (b=.60, p<.05), but not in females (b=.47, p>.05). Other types of abuse or neglect were not significant predictors of anger. |
| Teicher et al., 2006 [[103]](https://www.zotero.org/google-docs/?ZDLUnh) | Cross-sectional | 554 young adults from community | Self-reported abuse on Verbal Abuse Questionnaire & 3 items assessing physical abuse, sexual abuse, and witnessed domestic violence | Self-reported anger on SQ | Independent t test: Emotional (d=.63) and sexual abuse (d=.52) alone had a moderate effect on increased anger, but not physical abuse (d=.13, p>.40) Combined exposure to any two categories had a large effect size (d=.85), and combined exposure to all three types of abuse had the largest effect (d=1.12). |
| Teicher & Vitaliano, 2011 [[104]](https://www.zotero.org/google-docs/?i9UIlu) | Cross-sectional | 1,412 young adults with siblings | Self-reported abuse on Verbal Abuse Questionnaire & 3 items assessing physical abuse, sexual abuse, and witnessed domestic violence | Self-reported anger on SQ | Regression: Witnessing violence towards siblings (*β*=1.24, p<.001), parental verbal (*β*=1.36, p<.0001) and physical (*β*=.81, p<.05) abuse, and sibling verbal abuse (*β*=.78, p<.05) were significant predictors of increased anger-hostility. |
| Toda et al., 2018 [[33]](https://www.zotero.org/google-docs/?kRMf5X) | Cross-sectional | 245 adults (75 outpatients with bipolar disorder and 170 healthy controls) | Self-reported childhood abuse and trauma on CATS | Self-reported irritability on TEMPS-A | Correlation: Overall childhood abuse (r=.25/.42 for healthy controls and BD patients, p<.01) and neglect (r=.27/.43 for healthy controls and BD patients, p<.01)were positively correlated with irritability.Childhood sexual abuse (r=.28, p<.05) and punishment (r=.33, p<.01) were positively correlated with irritability only for patients with bipolar disorder.  Regression: Childhood neglect (β=.27/.34 for healthy controls and BD patients, (ps<.05) was a significant predictor of increased irritability. |
| Truhan & Armour, 2023 [[105]](https://www.zotero.org/google-docs/?n6NlWQ) | Cross-sectional | 590 adult men (veterans) in UK/Ireland | Self-reported childhood adversity on ACE-Q | Self-reported anger on DAR-7 | Correlation: child abuse (r=.23), neglect (r=.29) , and household challenge (r=.16) were not significantly correlated with anger (ps>.05).   Regression: Child abuse (β=.16-.19, p<.001) and neglect (β=.22-.28, p<.001) were significant predictors of increased anger (βs varied depending on covariates); household challenge (β=.09-.10, p<.05) was a significant predictor of increased anger, only when friend support was not entered as a covariate. |
| Veith et al., 2017 [[108]](https://www.zotero.org/google-docs/?7Eoz5h) | Cross-sectional | 526 adults from community | Self-reported childhood maltreatment on VEQ-R and sexual abuse and assault | Self-reported trait anger on BPAQ | Correlation: Childhood maltreatment (r=.17, p<.001) and sexual abuse (r=.17, p<.001) were positively correlated with trait anger. |
| Wakatsuki et al., 2020 [[109]](https://www.zotero.org/google-docs/?lYaden) | Cross-sectional | 204 adults from community in Japan | Self-reported childhood abuse and trauma on CATS | Self-reported irritability on TEMPS-A | Correlation: Childhood neglect (ρ=.54, p<.01), punishment (ρ=.22, p<.01), and sexual abuse (ρ=.18, p<.05) were positively correlated with irritability. |
| Walker et al., 2021 [[110]](https://www.zotero.org/google-docs/?p4w1fX) | Cross-sectional | 567 undergraduates | self-reported childhood sexual abuse on LSC-R | Self-reported anger on DAR-5 | Regression: Childhood sexual abuse was a significant predictor of increased anger (b=3.1, p=.02). |
| Wills et al., 2022 [[4]](https://www.zotero.org/google-docs/?Bw84pK) | Cross-sectional | 870 Latina adults | Self-reported childhood victimization on LTVH | Self-reported TSI anger/irritability | Regression: Total victimization (β=.14, p=.049) and childhood victimization perpetrated by family members (β=.12, p<.001) were significant predictors of increased anger-irritability. |
| Win et al., 2021 [[113]](https://www.zotero.org/google-docs/?iZkjwx) | Longitudinal | 3294 adults | Self-reported childhood maltreatment on CTQ | Self-reported trait anger on STAXI (assessed within a year of CTQ) | Correlation: Childhood maltreatment was positively correlated with trait anger-in (r=.16, p<.001) and trait anger-out (r=.18, p<.001). |
| Wuebken et al., 2023 [[114]](https://www.zotero.org/google-docs/?QNF48h) | Cross-sectional | 254 mothers from community in Germany | Clinician-reported child abuse on CAS | Self-reported trait anger on STAXI | Correlation: Childhood abuse was positively correlated with trait anger (r=.31, p<.01). |
| Yarar et al., 2023 [[116]](https://www.zotero.org/google-docs/?x2f267) | Cross-sectional | 184 undergraduates | Self-reported childhood maltreatment on CTQ | self-reported trait anger on Trait Anger and the Anger Expressions Scale | Correlation: Childhood maltreatment was positively correlated with trait anger in men (r=.23, p=.03) and women (r=.38, p<.001).   Regression: Childhood maltreatment significantly predicted trait anger (*β*=.08, p<.001). |
| Yaşar et al., 2020 [[117]](https://www.zotero.org/google-docs/?WZ1veQ) | Cross-sectional | 38 adults with epilepsy | Self-reported childhood maltreatment on CTQ | Self-reported irritability on TEMPS-A | Correlation: Overall childhood maltreatment (r=.33, p=.045) and emotional abuse (r=.39, p=.017) were positively correlated with irritability.   Mann-Whitney U test: Individuals with CTQ>=35 reported higher irritability than those with CTQ<35 (p=.046). |
| Zelikovsky & Lynn, 2002 [[118]](https://www.zotero.org/google-docs/?2kXK16) | Cross-sectional | 100 undergraduates (65 with childhood abuse history and 35 reported no abuse) | self-reported psychological abuse on the PsyMQ, physical abuse on the PhyMQ, and sexual abuse on the CSVQ | Self-reported trait anger on STAXI | One-way ANOVA: Individuals with combined psychological and physical abuse (vs. no abuse) reported increased trait anger (p<.01). There were no differences in trait anger between individuals with psychological abuse alone vs. combined abuse or no abuse. |

**Note.** Statistics, including nonsignificant findings, are included alongside descriptive results when reported. β refers to standardized regression coefficients and b refers to unstandardized regression coefficients, as reported. ρ refers to Spearman correlation coefficient and r refers to Pearson correlation coefficient. ANOVA=Analysis of Variance; CI=confidence interval; OR=odds ratio.

Abbreviations in the order of appearance: JVQ=Juvenile Victimization Questionnaire; DAR-5=Dimensions of Anger Reactions-5 Scale; CHQ=Childhood History Questionnaire; BPAQ-SF=Buss-Perry Aggression Questionnaire-Short Form; IBS=irritable bowel syndrome; CIDI=Composite International Diagnostic Interview; STAXI-2=State-Trait Anger Expression Inventory-2; ETI-SR=Early Trauma Inventory-Self Report; CCMS=Comprehensive Childhood Maltreatment Scale for Adults; STAXI=State-Trait Anger Expression Inventory; CTQ=Childhood Trauma Questionnaire; IRQ=Irritability Questionnaire; AFECTS=Affective and Emotional Composite Temperament Scale; RF=Risky Families Questionnaire; AQ-SF=Aggression Questionnaire-Short Form; EASE-PI=Exposure to Abusive and Support Environments: Parenting Inventory; AQ=Aggression Questionnaire; NOSIE-30=Nurses’ Observation Scale for Inpatient Evaluation-30; CTSPC=Conflict Tactics Scale-Parent/Child; CTI=Childhood Trauma Interview; CTQ-SF=Childhood Trauma Questionnaire-Short Form; BSI=Brief Symptom Inventory; ANPS=Affective Neuroscience Personality Scale; STAS=State-Trait Anger Scale; BPAQ=Buss-Perry Aggression Questionnaire; PMI=Psychological Maltreatment Inventory; MAI=Multidimensional Anger Inventory; CTS=Conflict Tactics Scale; EPAB=Emotional and Physical Abuse Questionnaire; FSCSE=Finkelhor’s Survey of Childhood Sexual Experiences; MAES=Maltreatment Abuse and Exposure Scale; CAS=Child Abuse Scale; BDHI=Buss-Durkee Hostility Inventory; CATS=Child Abuse and Trauma Scale; TEMPS-A=Temperament Evaluation of Memphis, Pisa, Paris, and San Diego; NAS=Novaco Anger Scale; PI=Provocation Inventory; WARS=Ward Anger Rating Scale; STEQ=Stressful and Traumatic Events Questionnaire; BPD=borderline personality disorder; PAS=Personality Assessment Screener; ASR=Adult Self-Report; CTSPC=Conflict Tactics Scale-Parent/Child; SQ=Kellner Symptom Questionnaire; DAR-7=Dimensions of Anger Reactions-7 Scale; VEQ-R=Violent Experiences Questionnaire-Revised; LSC-R=Life Stressor Checklist-Revised; LTVH=Lifetime Trauma and Victimization History; PsyMQ=Psychological Child Maltreatment Questionnaire; PhyMQ=Physical Child Maltreatment Questionnaire; CSVQ=Child Sexual Victimization Questionnaire.

**References**

[[1]](https://www.zotero.org/google-docs/?pX20iA)	[Henrich J, Heine SJ, Norenzayan A. The weirdest people in the world? Behav Brain Sci 2010;33:61–83. https://doi.org/10.1017/S0140525X0999152X.](https://www.zotero.org/google-docs/?pX20iA)

[[2]](https://www.zotero.org/google-docs/?pX20iA)	[Evans SZ, Simons LG, Simons RL. The Effect of Corporal Punishment and Verbal Abuse on Delinquency: Mediating Mechanisms. J Youth Adolesc 2012;41:1095–110. https://doi.org/10.1007/s10964-012-9755-x.](https://www.zotero.org/google-docs/?pX20iA)

[[3]](https://www.zotero.org/google-docs/?pX20iA)	[Porcerelli JH, Hurrell K, Cogan R, Jeffries K, Markova T. Personality Assessment Screener, Childhood Abuse, and Adult Partner Violence in African American Women Using Primary Care. Assessment 2015;22:749–52. https://doi.org/10.1177/1073191115606213.](https://www.zotero.org/google-docs/?pX20iA)

[[4]](https://www.zotero.org/google-docs/?pX20iA)	[Wills C, Cuevas CA, Sabina C. The role of the victim–offender relationship on psychological distress among Latinx women: A betrayal trauma perspective. Psychol Trauma Theory Res Pract Policy 2022;14:20–8. https://doi.org/10.1037/tra0000923.](https://www.zotero.org/google-docs/?pX20iA)

[[5]](https://www.zotero.org/google-docs/?pX20iA)	[Bernstein DP, Fink L, Handelsman L, Foote J. Childhood Trauma Questionnaire 2011. https://doi.org/10.1037/t02080-000.](https://www.zotero.org/google-docs/?pX20iA)

[[6]](https://www.zotero.org/google-docs/?pX20iA)	[Straus MA. Conflict Tactics Scales 2012. https://doi.org/10.1037/t02125-000.](https://www.zotero.org/google-docs/?pX20iA)

[[7]](https://www.zotero.org/google-docs/?pX20iA)	[Felitti VJ, Anda RF, Nordenberg D, Williamson DF, Spitz AM, Edwards V, Koss MP, Marks JS. Relationship of Childhood Abuse and Household Dysfunction to Many of the Leading Causes of Death in Adults. Am J Prev Med 1998;14:245–58. https://doi.org/10.1016/S0749-3797(98)00017-8.](https://www.zotero.org/google-docs/?pX20iA)

[[8]](https://www.zotero.org/google-docs/?pX20iA)	[Caprara GV, Cinanni V, D’Imperio G, Passerini S, Renzi P, Travaglia G. Indicators of impulsive aggression: Present status of research on irritability and emotional susceptibility scales. Personal Individ Differ 1985;6:665–74. https://doi.org/10.1016/0191-8869(85)90077-7.](https://www.zotero.org/google-docs/?pX20iA)

[[9]](https://www.zotero.org/google-docs/?pX20iA)	[Craig KJ, Hietanen H, Markova IS, Berrios GE. The Irritability Questionnaire: A new scale for the measurement of irritability. Psychiatry Res 2008;159:367–75. https://doi.org/10.1016/j.psychres.2007.03.002.](https://www.zotero.org/google-docs/?pX20iA)

[[10]](https://www.zotero.org/google-docs/?pX20iA)	[Stringaris A, Goodman R, Ferdinando S, Razdan V, Muhrer E, Leibenluft E, Brotman MA. The Affective Reactivity Index: a concise irritability scale for clinical and research settings. J Child Psychol Psychiatry 2012;53:1109–17. https://doi.org/10.1111/j.1469-7610.2012.02561.x.](https://www.zotero.org/google-docs/?pX20iA)

[[11]](https://www.zotero.org/google-docs/?pX20iA)	[Akiskal HS, Akiskal KK, Haykal RF, Manning JS, Connor PD. TEMPS-A: progress towards validation of a self-rated clinical version of the Temperament Evaluation of the Memphis, Pisa, Paris, and San Diego Autoquestionnaire. J Affect Disord 2005;85:3–16. https://doi.org/10.1016/j.jad.2004.12.001.](https://www.zotero.org/google-docs/?pX20iA)

[[12]](https://www.zotero.org/google-docs/?pX20iA)	[Boonmann C, Grisso T, Guy LS, Colins OF, Mulder EA, Vahl P, Jansen LMC, Doreleijers TAH, Vermeiren RRJM. Childhood traumatic experiences and mental health problems in sexually offending and non-sexually offending juveniles. Child Adolesc Psychiatry Ment Health 2016;10:45. https://doi.org/10.1186/s13034-016-0127-2.](https://www.zotero.org/google-docs/?pX20iA)

[[13]](https://www.zotero.org/google-docs/?pX20iA)	[Briere J, Johnson K, Bissada A, Damon L, Crouch J, Gil E, Hanson R, Ernst V. The Trauma Symptom Checklist for Young Children (TSCYC): reliability and association with abuse exposure in a multi-site study. Child Abuse Negl 2001;25:1001–14. https://doi.org/10.1016/S0145-2134(01)00253-8.](https://www.zotero.org/google-docs/?pX20iA)

[[14]](https://www.zotero.org/google-docs/?pX20iA)	[Spielberger CD. State‐Trait Anger Expression Inventory. In: Weiner IB, Craighead WE, editors. Corsini Encycl. Psychol. 1st ed., Wiley; 2010, p. 1–1. https://doi.org/10.1002/9780470479216.corpsy0942.](https://www.zotero.org/google-docs/?pX20iA)

[[15]](https://www.zotero.org/google-docs/?pX20iA)	[Forbes D, Alkemade N, Mitchell D, Elhai JD, McHugh T, Bates G, Novaco RW, Bryant R, Lewis V. UTILITY OF THE DIMENSIONS OF ANGER REACTIONS-5 (DAR-5) SCALE AS A BRIEF ANGER MEASURE: Research Article: Utility of DAR-5. Depress Anxiety 2014;31:166–73. https://doi.org/10.1002/da.22148.](https://www.zotero.org/google-docs/?pX20iA)

[[16]](https://www.zotero.org/google-docs/?pX20iA)	[Asgeirsdottir BB, Sigfusdottir ID, Gudjonsson GH, Sigurdsson JF. Associations between sexual abuse and family conflict/violence, self-injurious behavior, and substance use: The mediating role of depressed mood and anger. Child Abuse Negl 2011;35:210–9. https://doi.org/10.1016/j.chiabu.2010.12.003.](https://www.zotero.org/google-docs/?pX20iA)

[[17]](https://www.zotero.org/google-docs/?pX20iA)	[Coe JL, Micalizzi L, Josefson B, Parade SH, Seifer R, Tyrka AR. Sex differences in associations between early adversity, child temperament, and behavior problems. Int J Behav Dev 2020;44:490–504. https://doi.org/10.1177/0165025420912012.](https://www.zotero.org/google-docs/?pX20iA)

[[18]](https://www.zotero.org/google-docs/?pX20iA)	[Henriksen M, Skrove M, Hoftun GB, Sund ER, Lydersen S, Kalvin CB, Sukhodolsky DG. Exposure to traumatic events poses greater risk for irritability in girls than in boys. J Affect Disord Rep 2021;6:100204. https://doi.org/10.1016/j.jadr.2021.100204.](https://www.zotero.org/google-docs/?pX20iA)

[[19]](https://www.zotero.org/google-docs/?pX20iA)	[Kobulsky JM, Yoon S, Bright CL, Lee G, Nam B. Gender‐Moderated Pathways From Childhood Abuse and Neglect to Late‐Adolescent Substance Use. J Trauma Stress 2018;31:654–64. https://doi.org/10.1002/jts.22326.](https://www.zotero.org/google-docs/?pX20iA)

[[20]](https://www.zotero.org/google-docs/?pX20iA)	[Rizzo CJ, Esposito‐Smythers C, Swenson L, Hower HM, Wolff J, Spirito A. Dating Violence Victimization, Dispositional Aggression, and Nonsuicidal Self‐Injury among Psychiatrically Hospitalized Male and Female Adolescents. Suicide Life Threat Behav 2014;44:338–51. https://doi.org/10.1111/sltb.12081.](https://www.zotero.org/google-docs/?pX20iA)

[[21]](https://www.zotero.org/google-docs/?pX20iA)	[Sigfusdottir ID, Asgeirsdottir BB, Gudjonsson GH, Sigurdsson JF. Suicidal ideations and attempts among adolescents subjected to childhood sexual abuse and family conflict/violence: The mediating role of anger and depressed mood. J Adolesc 2013;36:1227–36. https://doi.org/10.1016/j.adolescence.2013.10.001.](https://www.zotero.org/google-docs/?pX20iA)

[[22]](https://www.zotero.org/google-docs/?pX20iA)	[Benedini KM, Fagan AA. From Child Maltreatment to Adolescent Substance Use: Different Pathways for Males and Females? Fem Criminol 2020;15:147–73. https://doi.org/10.1177/1557085118810426.](https://www.zotero.org/google-docs/?pX20iA)

[[23]](https://www.zotero.org/google-docs/?pX20iA)	[Cazala F, Bauer IE, Meyer TD, Spiker DE, Kazimi IF, Zeni CP, Zunta-Soares GB, Soares JC. Correlates of childhood trauma in children and adolescents with bipolar disorder spectrum: A preliminary study. J Affect Disord 2019;247:114–9. https://doi.org/10.1016/j.jad.2018.12.007.](https://www.zotero.org/google-docs/?pX20iA)

[[24]](https://www.zotero.org/google-docs/?pX20iA)	[Leban L, Delacruz DJ. Gendered Relationships Between Adverse Childhood Experiences, Negative Emotional States, and Violent Delinquency. J Interpers Violence 2023;38:9132–58. https://doi.org/10.1177/08862605231162664.](https://www.zotero.org/google-docs/?pX20iA)

[[25]](https://www.zotero.org/google-docs/?pX20iA)	[Wolfe DA, Scott K, Wekerle C, Pittman A-L. Child Maltreatment: Risk of Adjustment Problems and Dating Violence in Adolescence. J Am Acad Child Adolesc Psychiatry 2001;40:282–9. https://doi.org/10.1097/00004583-200103000-00007.](https://www.zotero.org/google-docs/?pX20iA)

[[26]](https://www.zotero.org/google-docs/?pX20iA)	[Harper FWK, Arias I. The Role of Shame in Predicting Adult Anger and Depressive Symptoms Among Victims of Child Psychological Maltreatment. J Fam Violence 2004;19:359–67. https://doi.org/10.1007/s10896-004-0681-x.](https://www.zotero.org/google-docs/?pX20iA)

[[27]](https://www.zotero.org/google-docs/?pX20iA)	[Almeida TC, Cardoso J, Matos AF, Murça A, Cunha O. Adverse childhood experiences and aggression in adulthood: The moderating role of positive childhood experiences. Child Abuse Negl 2024;154:106929. https://doi.org/10.1016/j.chiabu.2024.106929.](https://www.zotero.org/google-docs/?pX20iA)

[[28]](https://www.zotero.org/google-docs/?pX20iA)	[Stearns M, McKinney C. Perceived Parental Anxiety and Depressive Problems and Emerging Adult Oppositional Defiant Problems: Moderated Mediation by Psychological and Physical Maltreatment and Gender. Fam Process 2020;59:651–65. https://doi.org/10.1111/famp.12459.](https://www.zotero.org/google-docs/?pX20iA)

[[29]](https://www.zotero.org/google-docs/?pX20iA)	[Sudbrack R, Manfro PH, Kuhn IM, De Carvalho HW, Lara DR. What doesn’t kill you makes you stronger and weaker: How childhood trauma relates to temperament traits. J Psychiatr Res 2015;62:123–9. https://doi.org/10.1016/j.jpsychires.2015.01.001.](https://www.zotero.org/google-docs/?pX20iA)

[[30]](https://www.zotero.org/google-docs/?pX20iA)	[Rinne-Albers MAW, Van Der Werff SJA, Van Hoof M-J, Van Lang ND, Lamers-Winkelman F, Rombouts SA, Vermeiren RRJM, Van Der Wee NJA. Abnormalities of white matter integrity in the corpus callosum of adolescents with PTSD after childhood sexual abuse: a DTI study. Eur Child Adolesc Psychiatry 2016;25:869–78. https://doi.org/10.1007/s00787-015-0805-2.](https://www.zotero.org/google-docs/?pX20iA)

[[31]](https://www.zotero.org/google-docs/?pX20iA)	[Bilgi MM, Taspinar S, Aksoy B, Oguz K, Coburn K, Gonul AS. The relationship between childhood trauma, emotion recognition, and irritability in schizophrenia patients. Psychiatry Res 2017;251:90–6. https://doi.org/10.1016/j.psychres.2017.01.091.](https://www.zotero.org/google-docs/?pX20iA)

[[32]](https://www.zotero.org/google-docs/?pX20iA)	[Morandotti N, Dima D, Jogia J, Frangou S, Sala M, Vidovich GZD, Lazzaretti M, Gambini F, Marraffini E, d’Allio G, Barale F, Zappoli F, Caverzasi E, Brambilla P. Childhood abuse is associated with structural impairment in the ventrolateral prefrontal cortex and aggressiveness in patients with borderline personality disorder. Psychiatry Res Neuroimaging 2013;213:18–23. https://doi.org/10.1016/j.pscychresns.2013.02.002.](https://www.zotero.org/google-docs/?pX20iA)

[[33]](https://www.zotero.org/google-docs/?pX20iA)	[Toda H, Inoue T, Tanichi M, Saito T, Nakagawa S, Masuya J, Tanabe H, Yoshino A, Kusumi I. Affective temperaments play an important role in the relationship between child abuse and the diagnosis of bipolar disorder. Psychiatry Res 2018;262:13–9. https://doi.org/10.1016/j.psychres.2018.01.040.](https://www.zotero.org/google-docs/?pX20iA)

[[34]](https://www.zotero.org/google-docs/?pX20iA)	[De Bles NJ, Pütz LEH, Rius Ottenheim N, Van Hemert AM, Elzinga BM, Penninx BWJH, Giltay EJ. Childhood trauma and anger in adults with and without depressive and anxiety disorders. Acta Psychiatr Scand 2023;148:288–301. https://doi.org/10.1111/acps.13589.](https://www.zotero.org/google-docs/?pX20iA)

[[35]](https://www.zotero.org/google-docs/?pX20iA)	[Adler AB, LeardMann CA, Roenfeldt KA, Jacobson IG, Forbes D, for the Millennium Cohort Study Team. Magnitude of problematic anger and its predictors in the Millennium Cohort. BMC Public Health 2020;20:1168. https://doi.org/10.1186/s12889-020-09206-2.](https://www.zotero.org/google-docs/?pX20iA)

[[36]](https://www.zotero.org/google-docs/?pX20iA)	[Baron SW. Strain, Criminal Propensity, and Violence: Examining the Role of the Composite Moderator in Agnew’s Extension to GST. Crime Delinquency 2019;65:801–21. https://doi.org/10.1177/0011128718787511.](https://www.zotero.org/google-docs/?pX20iA)

[[37]](https://www.zotero.org/google-docs/?pX20iA)	[Barrett EL, Mills KL, Teesson M, Ewer P. Mental health correlates of anger and violence among individuals entering substance use treatment. Ment Health Subst Use 2013;6:287–302. https://doi.org/10.1080/17523281.2012.725425.](https://www.zotero.org/google-docs/?pX20iA)

[[38]](https://www.zotero.org/google-docs/?pX20iA)	[Beesley H, Rhodes J, Salmon P. Anger and childhood sexual abuse are independently associated with irritable bowel syndrome. Br J Health Psychol 2010;15:389–99. https://doi.org/10.1348/135910709X466496.](https://www.zotero.org/google-docs/?pX20iA)

[[39]](https://www.zotero.org/google-docs/?pX20iA)	[Berthelot N, Hébert M, Godbout N, Goulet M, Bergeron S, Boucher S. Childhood Maltreatment Increases the Risk of Intimate Partner Violence via PTSD and Anger Personality Traits in Individuals Consulting for Sexual Problems. J Aggress Maltreatment Trauma 2014;23:982–98. https://doi.org/10.1080/10926771.2014.960631.](https://www.zotero.org/google-docs/?pX20iA)

[[40]](https://www.zotero.org/google-docs/?pX20iA)	[Bielas H, Barra S, Skrivanek C, Aebi M, Steinhausen H-C, Bessler C, Plattner B. The associations of cumulative adverse childhood experiences and irritability with mental disorders in detained male adolescent offenders. Child Adolesc Psychiatry Ment Health 2016;10:34. https://doi.org/10.1186/s13034-016-0122-7.](https://www.zotero.org/google-docs/?pX20iA)

[[41]](https://www.zotero.org/google-docs/?pX20iA)	[Bock BB, Bastos CR, Ardais AP, Grellert M, De Carvalho HW, Farias CP, Jansen K, Oses JP, Da Silva RA, Portela LV, Kaster MP, Lara DR, Ghisleni G. Temperament traits moderate the relationship between Childhood Trauma and Interleukin 1β profile in young adults. Psychoneuroendocrinology 2020;116:104671. https://doi.org/10.1016/j.psyneuen.2020.104671.](https://www.zotero.org/google-docs/?pX20iA)

[[42]](https://www.zotero.org/google-docs/?pX20iA)	[Brenner J, Pan Z, Mazefsky C, Smith KA, Gabriels R, Siegel M, Erickson C, Gabriels RL, Kaplan D, Mazefsky C, Morrow EM, Righi G, Santangelo SL, Wink L, Benevides J, Beresford C, Best C, Bowen K, Dechant B, Flis T, Gastgeb H, Geer A, Hagopian L, Handen B, Klever A, Lubetsky M, MacKenzie K, Meservy Z, McGonigle J, McGuire K, McNeil F, Pedapati E, Peura C, Pierri J, Rogers C, Rossman B, Ruberg J, Small C, Smith KA, Stuckey N, Tylenda B, Verdi M, Vezzoli J, Williams D, Williams D, for the Autism and Developmental Disorders Inpatient Research Collaborative (ADDIRC). Behavioral Symptoms of Reported Abuse in Children and Adolescents with Autism Spectrum Disorder in Inpatient Settings. J Autism Dev Disord 2018;48:3727–35. https://doi.org/10.1007/s10803-017-3183-4.](https://www.zotero.org/google-docs/?pX20iA)

[[43]](https://www.zotero.org/google-docs/?pX20iA)	[Brezina T. Adolescent Maltreatment and Delinquency: The Question of Intervening Processes. J Res Crime Delinquency 1998;35:71–99. https://doi.org/10.1177/0022427898035001003.](https://www.zotero.org/google-docs/?pX20iA)

[[44]](https://www.zotero.org/google-docs/?pX20iA)	[Carver CS, Johnson SL, McCullough ME, Forster DE, Joormann J. Adulthood personality correlates of childhood adversity. Front Psychol 2014;5. https://doi.org/10.3389/fpsyg.2014.01357.](https://www.zotero.org/google-docs/?pX20iA)

[[45]](https://www.zotero.org/google-docs/?pX20iA)	[Cascardi M, Jouriles EN, Temple JR. Distinct and Overlapping Correlates of Psychological and Physical Partner Violence Perpetration. J Interpers Violence 2020;35:2375–98. https://doi.org/10.1177/0886260517702492.](https://www.zotero.org/google-docs/?pX20iA)

[[46]](https://www.zotero.org/google-docs/?pX20iA)	[Cecil CAM, Viding E, Barker ED, Guiney J, McCrory EJ. Double disadvantage: the influence of childhood maltreatment and community violence exposure on adolescent mental health. J Child Psychol Psychiatry 2014;55:839–48. https://doi.org/10.1111/jcpp.12213.](https://www.zotero.org/google-docs/?pX20iA)

[[47]](https://www.zotero.org/google-docs/?pX20iA)	[Choi K-H, Reddy LF, Liu NH, Spaulding WD. Memory as a Moderator in the Relationship Between Child Sexual Abuse and Maladaptive Functioning in People With Severe Mental Illness. J Nerv Ment Dis 2009;197:282–6. https://doi.org/10.1097/NMD.0b013e31819dc316.](https://www.zotero.org/google-docs/?pX20iA)

[[48]](https://www.zotero.org/google-docs/?pX20iA)	[Chong LS, Senich KL, Ahmad S, Olezeski CL, Gordis EB. Psychologically aggressive parenting and later aggression: Salivary alpha-amylase reactivity and sex as moderators. Int J Psychophysiol 2023;194:112260. https://doi.org/10.1016/j.ijpsycho.2023.112260.](https://www.zotero.org/google-docs/?pX20iA)

[[49]](https://www.zotero.org/google-docs/?pX20iA)	[Collin-Vézina D, Coleman K, Milne L, Sell J, Daigneault I. Trauma Experiences, Maltreatment-Related Impairments, and Resilience Among Child Welfare Youth in Residential Care. Int J Ment Health Addict 2011;9:577–89. https://doi.org/10.1007/s11469-011-9323-8.](https://www.zotero.org/google-docs/?pX20iA)

[[50]](https://www.zotero.org/google-docs/?pX20iA)	[Cooley JL, Taussig HN. Anger and Attention Problems as Mechanisms Linking Maltreatment Subtypes and Witnessed Violence to Social Functioning Among Children in Out-of-Home Care. Child Maltreat 2022;27:647–57. https://doi.org/10.1177/10775595211038926.](https://www.zotero.org/google-docs/?pX20iA)

[[51]](https://www.zotero.org/google-docs/?pX20iA)	[Cotter G, Morreale K, Valdegas A, Fish M, Beebe R, Grasso D, Stover C, Tseng W. Associations between trauma exposure and irritability within the family unit: a network approach. J Child Psychol Psychiatry 2024;65:1501–12. https://doi.org/10.1111/jcpp.13998.](https://www.zotero.org/google-docs/?pX20iA)

[[52]](https://www.zotero.org/google-docs/?pX20iA)	[Derella OJ, Burke JD, Stepp SD, Hipwell AE. Reciprocity in Undesirable Parent–Child Behavior? Verbal Aggression, Corporal Punishment, and Girls’ Oppositional Defiant Symptoms. J Clin Child Adolesc Psychol 2020;49:420–33. https://doi.org/10.1080/15374416.2019.1603109.](https://www.zotero.org/google-docs/?pX20iA)

[[53]](https://www.zotero.org/google-docs/?pX20iA)	[DiLillo D, Tremblay GC, Peterson L. Linking childhood sexual abuse and abusive parenting: the mediating role of maternal anger. Child Abuse Negl 2000;24:767–79. https://doi.org/10.1016/S0145-2134(00)00138-1.](https://www.zotero.org/google-docs/?pX20iA)

[[54]](https://www.zotero.org/google-docs/?pX20iA)	[Etchin AG, Corbo V, Brown E, Fortier CB, Fonda JR, Milberg WP, Currao A, McGlinchey RE. Associations among clinical variables and anger differ by early life adversity among post‐9/11 veterans. Clin Psychol Psychother 2022;29:1403–15. https://doi.org/10.1002/cpp.2722.](https://www.zotero.org/google-docs/?pX20iA)

[[55]](https://www.zotero.org/google-docs/?pX20iA)	[Faulkner B, Goldstein AL, Wekerle C. Pathways From Childhood Maltreatment to Emerging Adulthood: Investigating Trauma-Mediated Substance Use and Dating Violence Outcomes Among Child Protective Services–Involved Youth. Child Maltreat 2014;19:219–32. https://doi.org/10.1177/1077559514551944.](https://www.zotero.org/google-docs/?pX20iA)

[[56]](https://www.zotero.org/google-docs/?pX20iA)	[Feiring C, Simon VA, Cleland CM, Barrett EP. Potential Pathways From Stigmatization and Externalizing Behavior to Anger and Dating Aggression in Sexually Abused Youth. J Clin Child Adolesc Psychol 2013;42:309–22. https://doi.org/10.1080/15374416.2012.736083.](https://www.zotero.org/google-docs/?pX20iA)

[[57]](https://www.zotero.org/google-docs/?pX20iA)	[Fitzgerald M. Developmental Pathways from Childhood Maltreatment to Young Adult Romantic Relationship Functioning. J Trauma Dissociation 2021;22:581–97. https://doi.org/10.1080/15299732.2020.1869653.](https://www.zotero.org/google-docs/?pX20iA)

[[58]](https://www.zotero.org/google-docs/?pX20iA)	[Fitzgerald M, Williams L. When childhood states become adult traits: Trait anxiety and anger as mediators linking childhood maltreatment to marital outcomes in midlife adults. Psychol Violence 2024;14:219–27. https://doi.org/10.1037/vio0000463.](https://www.zotero.org/google-docs/?pX20iA)

[[59]](https://www.zotero.org/google-docs/?pX20iA)	[Fuchshuber J, Hiebler-Ragger M, Kresse A, Kapfhammer H-P, Unterrainer HF. The Influence of Attachment Styles and Personality Organization on Emotional Functioning After Childhood Trauma. Front Psychiatry 2019;10:643. https://doi.org/10.3389/fpsyt.2019.00643.](https://www.zotero.org/google-docs/?pX20iA)

[[60]](https://www.zotero.org/google-docs/?pX20iA)	[Gardner FL, Moore ZE, Dettore M. The Relationship Between Anger, Childhood Maltreatment, and Emotion Regulation Difficulties in Intimate Partner and Non-Intimate Partner Violent Offenders. Behav Modif 2014;38:779–800. https://doi.org/10.1177/0145445514539346.](https://www.zotero.org/google-docs/?pX20iA)

[[61]](https://www.zotero.org/google-docs/?pX20iA)	[Gause NK, Sales JM, Brown JL, Pelham WE, Liu Y, West SG. The protective role of secure attachment in the relationship between experiences of childhood abuse, emotion dysregulation and coping, and behavioral and mental health problems among emerging adult Black women: A moderated mediation analysis. J Psychopathol Clin Sci 2022;131:716–26. https://doi.org/10.1037/abn0000772.](https://www.zotero.org/google-docs/?pX20iA)

[[62]](https://www.zotero.org/google-docs/?pX20iA)	[Gerhant A, Olajossy M. Personality traits in alcohol-dependent individuals in the context of childhood abuse. Psychiatr Pol 2016;50:973–87. https://doi.org/10.12740/PP/60346.](https://www.zotero.org/google-docs/?pX20iA)

[[63]](https://www.zotero.org/google-docs/?pX20iA)	[Glück TM, Knefel M, Lueger-Schuster B. A network analysis of anger, shame, proposed ICD-11 post-traumatic stress disorder, and different types of childhood trauma in foster care settings in a sample of adult survivors. Eur J Psychotraumatology 2017;8:1372543. https://doi.org/10.1080/20008198.2017.1372543.](https://www.zotero.org/google-docs/?pX20iA)

[[64]](https://www.zotero.org/google-docs/?pX20iA)	[Grasser LR, Yang R, Brotman MA, Wiggins JL. The contribution of childhood trauma to irritability symptoms. JCPP Adv 2024:e12260. https://doi.org/10.1002/jcv2.12260.](https://www.zotero.org/google-docs/?pX20iA)

[[65]](https://www.zotero.org/google-docs/?pX20iA)	[Gunnlaugsson G, Kristjánsson ÁL, Einarsdóttir J, Sigfúsdóttir ID. Intrafamilial conflict and emotional well-being: A population based study among Icelandic adolescents. Child Abuse Negl 2011;35:372–81. https://doi.org/10.1016/j.chiabu.2011.01.011.](https://www.zotero.org/google-docs/?pX20iA)

[[66]](https://www.zotero.org/google-docs/?pX20iA)	[Hamstra C, Fitzgerald M. Longitudinal Effects from Childhood Abuse to Bullying Perpetration in Adolescence: The Role of Mental Health and Social Problems. J Child Adolesc Trauma 2022;15:869–81. https://doi.org/10.1007/s40653-021-00409-2.](https://www.zotero.org/google-docs/?pX20iA)

[[67]](https://www.zotero.org/google-docs/?pX20iA)	[Hasler R, Perroud N, Baud P, Olié E, Guillaume S, Malafosse A, Courtet P. *CREB1* modulates the influence of childhood sexual abuse on adult’s anger traits. Genes Brain Behav 2012;11:720–6. https://doi.org/10.1111/j.1601-183X.2012.00807.x.](https://www.zotero.org/google-docs/?pX20iA)

[[68]](https://www.zotero.org/google-docs/?pX20iA)	[Herrenkohl TI, Klika JB, Herrenkohl RC, Russo MJ, Dee T. A Prospective Investigation of the Relationship Between Child Maltreatment and Indicators of Adult Psychological Well-Being. Violence Vict 2012;27:764–76. https://doi.org/10.1891/0886-6708.27.5.764.](https://www.zotero.org/google-docs/?pX20iA)

[[69]](https://www.zotero.org/google-docs/?pX20iA)	[Hobfoll SE, Bansal A, Schurg R, Young S, Pierce CA, Hobfoll I, Johnson R. The impact of perceived child physical and sexual abuse history on Native American women’s psychological well-being and AIDS risk. J Consult Clin Psychol 2002;70:252–7. https://doi.org/10.1037/0022-006X.70.1.252.](https://www.zotero.org/google-docs/?pX20iA)

[[70]](https://www.zotero.org/google-docs/?pX20iA)	[Johnsona RM, Kotch JB, Catellier DJ, Winsor JR, Dufort V, Hunter W, Amaya-Jackson L. Adverse Behavioral and Emotional Outcomes from Child Abuse and Witnessed Violence. Child Maltreat 2002;7:179–86. https://doi.org/10.1177/1077559502007003001.](https://www.zotero.org/google-docs/?pX20iA)

[[71]](https://www.zotero.org/google-docs/?pX20iA)	[Keene AC, Epps J. Childhood physical abuse and aggression: Shame and narcissistic vulnerability. Child Abuse Negl 2016;51:276–83. https://doi.org/10.1016/j.chiabu.2015.09.012.](https://www.zotero.org/google-docs/?pX20iA)

[[72]](https://www.zotero.org/google-docs/?pX20iA)	[Kendra R, Bell KM, Guimond JM. The Impact of Child Abuse History, PTSD Symptoms, and Anger Arousal on Dating Violence Perpetration Among College Women. J Fam Violence 2012;27:165–75. https://doi.org/10.1007/s10896-012-9415-7.](https://www.zotero.org/google-docs/?pX20iA)

[[73]](https://www.zotero.org/google-docs/?pX20iA)	[Li N, Shelton M, Grigorenko EL. Adverse childhood experiences, anger, and violence among justice-involved youth: A network analysis. Child Abuse Negl 2025;161:107301. https://doi.org/10.1016/j.chiabu.2025.107301.](https://www.zotero.org/google-docs/?pX20iA)

[[74]](https://www.zotero.org/google-docs/?pX20iA)	[Litrownik AJ, Lau A, English DJ, Briggs E, Newton RR, Romney S, Dubowitz H. Measuring the severity of child maltreatment. Child Abuse Negl 2005;29:553–73. https://doi.org/10.1016/j.chiabu.2003.08.010.](https://www.zotero.org/google-docs/?pX20iA)

[[75]](https://www.zotero.org/google-docs/?pX20iA)	[Macarenco M-M, Opariuc-Dan C, Nedelcea C. Childhood trauma, dissociation, alexithymia, and anger in people with autoimmune diseases: A mediation model. Child Abuse Negl 2021;122:105322. https://doi.org/10.1016/j.chiabu.2021.105322.](https://www.zotero.org/google-docs/?pX20iA)

[[76]](https://www.zotero.org/google-docs/?pX20iA)	[Malvaso C, Day A, Casey S, Corrado R. Young Offenders, Maltreatment, and Trauma: A Pilot Study. Psychiatry Psychol Law 2017;24:458–69. https://doi.org/10.1080/13218719.2016.1247682.](https://www.zotero.org/google-docs/?pX20iA)

[[77]](https://www.zotero.org/google-docs/?pX20iA)	[Marie-Mitchell A, Watkins HBR, Copado IA, Distelberg B. Use of the Whole Child Assessment to identify children at risk of poor outcomes. Child Abuse Negl 2020;104:104489. https://doi.org/10.1016/j.chiabu.2020.104489.](https://www.zotero.org/google-docs/?pX20iA)

[[78]](https://www.zotero.org/google-docs/?pX20iA)	[Mills MS, Embury CM, Klanecky AK, Khanna MM, Calhoun VD, Stephen JM, Wang Y-P, Wilson TW, Badura-Brack AS. Traumatic Events Are Associated with Diverse Psychological Symptoms in Typically-Developing Children. J Child Adolesc Trauma 2020;13:381–8. https://doi.org/10.1007/s40653-019-00284-y.](https://www.zotero.org/google-docs/?pX20iA)

[[79]](https://www.zotero.org/google-docs/?pX20iA)	[Moore SM, Welsh MC, Peterson E. History of Childhood Maltreatment: Associations with Aggression and College Outcomes. J Aggress Maltreatment Trauma 2020;29:111–28. https://doi.org/10.1080/10926771.2019.1637989.](https://www.zotero.org/google-docs/?pX20iA)

[[80]](https://www.zotero.org/google-docs/?pX20iA)	[Murphy D, Turgoose D. Childhood adversity and mental health in veterans seeking treatment for mental health difficulties: Comparisons with the general military population. Psychol Trauma Theory Res Pract Policy 2022;14:805–11. https://doi.org/10.1037/tra0000538.](https://www.zotero.org/google-docs/?pX20iA)

[[81]](https://www.zotero.org/google-docs/?pX20iA)	[Nakai Y, Inoue T, Toda H, Toyomaki A, Nakato Y, Nakagawa S, Kitaichi Y, Kameyama R, Hayashishita Y, Wakatsuki Y, Oba K, Tanabe H, Kusumi I. The influence of childhood abuse, adult stressful life events and temperaments on depressive symptoms in the nonclinical general adult population. J Affect Disord 2014;158:101–7. https://doi.org/10.1016/j.jad.2014.02.004.](https://www.zotero.org/google-docs/?pX20iA)

[[82]](https://www.zotero.org/google-docs/?pX20iA)	[Novaco RW, Taylor JL. Anger and assaultiveness of male forensic patients with developmental disabilities: links to volatile parents. Aggress Behav 2008;34:380–93. https://doi.org/10.1002/ab.20254.](https://www.zotero.org/google-docs/?pX20iA)

[[83]](https://www.zotero.org/google-docs/?pX20iA)	[Osvath P, Vörös V, Fekete S. Life Events and Psychopathology in a Group of Suicide Attempters. Psychopathology 2004;37:36–40. https://doi.org/10.1159/000077018.](https://www.zotero.org/google-docs/?pX20iA)

[[84]](https://www.zotero.org/google-docs/?pX20iA)	[Otsuka A, Takaesu Y, Sato M, Masuya J, Ichiki M, Kusumi I, Inoue T. Interpersonal sensitivity mediates the effects of child abuse and affective temperaments on depressive symptoms in the general adult population. Neuropsychiatr Dis Treat 2017;Volume 13:2559–68. https://doi.org/10.2147/NDT.S144788.](https://www.zotero.org/google-docs/?pX20iA)

[[85]](https://www.zotero.org/google-docs/?pX20iA)	[Papalia N, Baidawi S, Luebbers S, Shepherd S, Ogloff JRP. Patterns of Maltreatment Co-Occurrence in Incarcerated Youth in Australia. J Interpers Violence 2022;37:NP4341–71. https://doi.org/10.1177/0886260520958639.](https://www.zotero.org/google-docs/?pX20iA)

[[86]](https://www.zotero.org/google-docs/?pX20iA)	[Peng C, Guo T, Cheng J, Wang M, Tan Y, Rong F, Kang C, Ding H, Wang Y, Yu Y. Association between childhood physical abuse and suicidal behaviors among Chinese adolescents: The mediation of aggression. J Affect Disord 2022;318:338–46. https://doi.org/10.1016/j.jad.2022.09.021.](https://www.zotero.org/google-docs/?pX20iA)

[[87]](https://www.zotero.org/google-docs/?pX20iA)	[Perroud N, Jaussent I, Guillaume S, Bellivier F, Baud P, Jollant F, Leboyer M, Lewis CM, Malafosse A, Courtet P. COMT but not serotonin‐related genes modulates the influence of childhood abuse on anger traits. Genes Brain Behav 2010;9:193–202. https://doi.org/10.1111/j.1601-183X.2009.00547.x.](https://www.zotero.org/google-docs/?pX20iA)

[[88]](https://www.zotero.org/google-docs/?pX20iA)	[Peterson ZD, Janssen E, Goodrich D, Fortenberry JD, Hensel DJ, Heiman JR. Child Sexual Abuse and Negative Affect as Shared Risk Factors for Sexual Aggression and Sexual HIV Risk Behavior in Heterosexual Men. Arch Sex Behav 2018;47:465–80. https://doi.org/10.1007/s10508-017-1079-1.](https://www.zotero.org/google-docs/?pX20iA)

[[89]](https://www.zotero.org/google-docs/?pX20iA)	[Plattner B, Karnik N, Jo B, Hall RE, Schallauer A, Carrion V, Feucht M, Steiner H. State and Trait Emotions in Delinquent Adolescents. Child Psychiatry Hum Dev 2007;38:155–69. https://doi.org/10.1007/s10578-007-0050-0.](https://www.zotero.org/google-docs/?pX20iA)

[[90]](https://www.zotero.org/google-docs/?pX20iA)	[Qirjako G, Dika Q, Mone I, Draçini X, Kuneshka L, Roshi E, Burazeri G. Correlates of Lifetime Physical Abuse Among Schoolchildren Aged 15 Years in Post-communist Albania. Front Public Health 2021;9:607493. https://doi.org/10.3389/fpubh.2021.607493.](https://www.zotero.org/google-docs/?pX20iA)

[[91]](https://www.zotero.org/google-docs/?pX20iA)	[Quenneville AF, Kalogeropoulou E, Küng A-L, Hasler R, Nicastro R, Prada P, Perroud N. Childhood maltreatment, anxiety disorders and outcome in borderline personality disorder. Psychiatry Res 2020;284:112688. https://doi.org/10.1016/j.psychres.2019.112688.](https://www.zotero.org/google-docs/?pX20iA)

[[92]](https://www.zotero.org/google-docs/?pX20iA)	[Rapoza KA, Wilson DT, Widmann WA, Riley MA, Robertson TW, Maiello E, Villot N, Manzella DJ, Ortiz-Garcia AL. The relationship between adult health and childhood maltreatment, as moderated by anger and ethnic background. Child Abuse Negl 2014;38:445–56. https://doi.org/10.1016/j.chiabu.2014.01.009.](https://www.zotero.org/google-docs/?pX20iA)

[[93]](https://www.zotero.org/google-docs/?pX20iA)	[Renner LM, Schwab-Reese LM, Coppola EC, Boel-Studt S. The contribution of interpersonal violence victimization types to psychological distress among youth. Child Abuse Negl 2020;106:104493. https://doi.org/10.1016/j.chiabu.2020.104493.](https://www.zotero.org/google-docs/?pX20iA)

[[94]](https://www.zotero.org/google-docs/?pX20iA)	[Roe-Sepowitz D, Krysik J. Examining the sexual offenses of female juveniles: The relevance of childhood maltreatment. Am J Orthopsychiatry 2008;78:405–12. https://doi.org/10.1037/a0014310.](https://www.zotero.org/google-docs/?pX20iA)

[[95]](https://www.zotero.org/google-docs/?pX20iA)	[Schneider MW, Ross A, Graham JC, Zielinski A. Do allegations of emotional maltreatment predict developmental outcomes beyond that of other forms of maltreatment? Child Abuse Negl 2005;29:513–32. https://doi.org/10.1016/j.chiabu.2004.08.010.](https://www.zotero.org/google-docs/?pX20iA)

[[96]](https://www.zotero.org/google-docs/?pX20iA)	[Sebre S, Sprugevica I, Novotni A, Bonevski D, Pakalniskiene V, Popescu D, Turchina T, Friedrich W, Lewis O. Cross-cultural comparisons of child-reported emotional and physical abuse: rates, risk factors and psychosocial symptoms. Child Abuse Negl 2004;28:113–27. https://doi.org/10.1016/j.chiabu.2003.06.004.](https://www.zotero.org/google-docs/?pX20iA)

[[97]](https://www.zotero.org/google-docs/?pX20iA)	[Sevenoaks T, Fouche J-P, Phillips N, Heany S, Myer L, Zar HJ, Stein DJ, Hoare J. Childhood Trauma and Mental Health in the Cape Town Adolescent Antiretroviral Cohort. J Child Adolesc Trauma 2022;15:353–63. https://doi.org/10.1007/s40653-021-00362-0.](https://www.zotero.org/google-docs/?pX20iA)

[[98]](https://www.zotero.org/google-docs/?pX20iA)	[Showalter K, Yoon S, Maguire‐Jack K, Wolf KG, Letson M. Are dual and single exposures differently associated with clinical levels of trauma symptoms? Examining physical abuse and witnessing intimate partner violence among young children. Child Fam Soc Work 2020;25:439–47. https://doi.org/10.1111/cfs.12700.](https://www.zotero.org/google-docs/?pX20iA)

[[99]](https://www.zotero.org/google-docs/?pX20iA)	[Snyder DK, Cigrang JA, Balderrama-Durbin C, Spierling TN, Alsaif HK, Kidd KR, Slep AMS, Heyman RE, Lorber MF, Eckardt AC, Mitnick DM, Waggoner JW. Resisting the cycle of violence: Impact of childhood abuse and neglect on individual and relationship functioning in early career enlisted air force male service members. Psychol Men Masculinities 2025;26:218–27. https://doi.org/10.1037/men0000503.](https://www.zotero.org/google-docs/?pX20iA)

[[100]](https://www.zotero.org/google-docs/?pX20iA)	[Solakoglu O, Driver N, Belshaw SH. The Effect of Sexual Abuse on Deviant Behaviors Among Turkish Adolescents: The Mediating Role of Emotions. Int J Offender Ther Comp Criminol 2018;62:24–49. https://doi.org/10.1177/0306624X16642810.](https://www.zotero.org/google-docs/?pX20iA)

[[101]](https://www.zotero.org/google-docs/?pX20iA)	[Springer KW, Sheridan J, Kuo D, Carnes M. Long-term physical and mental health consequences of childhood physical abuse: Results from a large population-based sample of men and women. Child Abuse Negl 2007;31:517–30. https://doi.org/10.1016/j.chiabu.2007.01.003.](https://www.zotero.org/google-docs/?pX20iA)

[[102]](https://www.zotero.org/google-docs/?pX20iA)	[Tang L, Ye X, Yan Q, Chang H, Ma Y, Liu D, Li Z, Yu Y. Factors associated with trait anger level of juvenile offenders in Hubei province: A binary logistic regression analysis. J Huazhong Univ Sci Technolog Med Sci 2017;37:20–4. https://doi.org/10.1007/s11596-017-1688-x.](https://www.zotero.org/google-docs/?pX20iA)

[[103]](https://www.zotero.org/google-docs/?pX20iA)	[Teicher MH, Samson JA, Polcari A, McGreenery CE. Sticks, Stones, and Hurtful Words: Relative Effects of Various Forms of Childhood Maltreatment. Am J Psychiatry 2006;163:993–1000. https://doi.org/10.1176/ajp.2006.163.6.993.](https://www.zotero.org/google-docs/?pX20iA)

[[104]](https://www.zotero.org/google-docs/?pX20iA)	[Teicher MH, Vitaliano GD. Witnessing Violence Toward Siblings: An Understudied but Potent Form of Early Adversity. PLoS ONE 2011;6:e28852. https://doi.org/10.1371/journal.pone.0028852.](https://www.zotero.org/google-docs/?pX20iA)

[[105]](https://www.zotero.org/google-docs/?pX20iA)	[Truhan TE, Armour C. Perceived friendships protect against the development of anger following childhood adversities in UK military veteran men residing in Northern Ireland. Eur J Psychotraumatology 2023;14:2289286. https://doi.org/10.1080/20008066.2023.2289286.](https://www.zotero.org/google-docs/?pX20iA)

[[106]](https://www.zotero.org/google-docs/?pX20iA)	[Turner HA, Finkelhor D, Ormrod R. The effect of lifetime victimization on the mental health of children and adolescents. Soc Sci Med 2006;62:13–27. https://doi.org/10.1016/j.socscimed.2005.05.030.](https://www.zotero.org/google-docs/?pX20iA)

[[107]](https://www.zotero.org/google-docs/?pX20iA)	[Van Vugt E, Lanctôt N, Paquette G, Collin-Vézina D, Lemieux A. Girls in residential care: From child maltreatment to trauma-related symptoms in emerging adulthood. Child Abuse Negl 2014;38:114–22. https://doi.org/10.1016/j.chiabu.2013.10.015.](https://www.zotero.org/google-docs/?pX20iA)

[[108]](https://www.zotero.org/google-docs/?pX20iA)	[Veith AC, Russell TD, King AR. PID-5 trait mediation of childhood maltreatment effects. Personal Individ Differ 2017;104:58–63. https://doi.org/10.1016/j.paid.2016.07.024.](https://www.zotero.org/google-docs/?pX20iA)

[[109]](https://www.zotero.org/google-docs/?pX20iA)	[Wakatsuki Y, Inoue T, Hashimoto N, Fujimura Y, Masuya J, Ichiki M, Tanabe H, Kusumi I. Influence of Childhood Maltreatment, Adulthood Stressful Life Events, and Affective Temperaments on Premenstrual Mental Symptoms of Nonclinical Adult Volunteers. Neuropsychiatr Dis Treat 2020;Volume 16:1–10. https://doi.org/10.2147/NDT.S232925.](https://www.zotero.org/google-docs/?pX20iA)

[[110]](https://www.zotero.org/google-docs/?pX20iA)	[Walker HE, Wamser-Nanney R, Howell KH. Child Sexual Abuse and Adult Sexual Assault among Emerging Adults: Exploring the Roles of Posttraumatic Stress Symptoms, Emotion Regulation, and Anger. J Child Sex Abuse 2021;30:407–26. https://doi.org/10.1080/10538712.2021.1890295.](https://www.zotero.org/google-docs/?pX20iA)

[[111]](https://www.zotero.org/google-docs/?pX20iA)	[Wang X, Zhao F, Yang J, Gao L, Li B, Lei L, Wang P. Childhood Maltreatment and Bullying Perpetration among Chinese Adolescents: A Moderated Mediation Model of Moral Disengagement and Trait Anger. Child Abuse Negl 2020;106:104507. https://doi.org/10.1016/j.chiabu.2020.104507.](https://www.zotero.org/google-docs/?pX20iA)

[[112]](https://www.zotero.org/google-docs/?pX20iA)	[White CR, English D, Thompson R, Roberts YH. Youth self-report of emotional maltreatment: Concordance with official reports and relation to outcomes. Child Youth Serv Rev 2016;62:111–21. https://doi.org/10.1016/j.childyouth.2016.02.004.](https://www.zotero.org/google-docs/?pX20iA)

[[113]](https://www.zotero.org/google-docs/?pX20iA)	[Win E, Zainal NH, Newman MG. Trait anger expression mediates childhood trauma predicting for adulthood anxiety, depressive, and alcohol use disorders. J Affect Disord 2021;288:114–21. https://doi.org/10.1016/j.jad.2021.03.086.](https://www.zotero.org/google-docs/?pX20iA)

[[114]](https://www.zotero.org/google-docs/?pX20iA)	[Wuebken K, Bermpohl F, Boedeker K, Hindi Attar C, Kluczniok D, Schoofs N, Fuchs A, Neukel C, Herpertz SC, Brunner R, Winter SM, Kaess M, Jaite C, Dittrich K. The mediating role of attachment and anger: exploring the impact of maternal early-life maltreatment on child abuse potential. Front Psychiatry 2023;14:1267038. https://doi.org/10.3389/fpsyt.2023.1267038.](https://www.zotero.org/google-docs/?pX20iA)

[[115]](https://www.zotero.org/google-docs/?pX20iA)	[Xu B, Li Y, Li Y, Xie J, Ding H, Wang J, Su P, Wang G. Association Between Child Maltreatment and Aggression in Chinese Early Adolescents: The Mediating Role of Irritability. J Interpers Violence 2024;39:393–413. https://doi.org/10.1177/08862605231197141.](https://www.zotero.org/google-docs/?pX20iA)

[[116]](https://www.zotero.org/google-docs/?pX20iA)	[Yarar EZ, Bulut BP, Demirbaş H. Does Gender Really Matter: Childhood Trauma, Trait Anger, and Suicide Risk in Early Adulthood. J Interpers Violence 2023;38:1222–38. https://doi.org/10.1177/08862605221087704.](https://www.zotero.org/google-docs/?pX20iA)

[[117]](https://www.zotero.org/google-docs/?pX20iA)	[Yaşar AB, Sayman C, Erdoğan Taycan S, Çeti̇Nkaya Y, Gündüz A, Ti̇Reli̇ H. vThe association between temperament features and childhood traumas in patients with juvenile myoclonic epilepsy. Turk J Med Sci 2020;50:1314–22. https://doi.org/10.3906/sag-1912-18.](https://www.zotero.org/google-docs/?pX20iA)

[[118]](https://www.zotero.org/google-docs/?pX20iA)	[Zelikovsky N, Lynn SJ. Childhood Psychological and Physical Abuse: Psychopathology, Dissociation, and Axis I Diagnosis. J Trauma Dissociation 2002;3:27–58. https://doi.org/10.1300/J229v03n03_03.](https://www.zotero.org/google-docs/?pX20iA)

[[119]](https://www.zotero.org/google-docs/?pX20iA)	[Zhou X, Zhen R. How do physical and emotional abuse affect depression and problematic behaviors in adolescents? The roles of emotional regulation and anger. Child Abuse Negl 2022;129:105641. https://doi.org/10.1016/j.chiabu.2022.105641.](https://www.zotero.org/google-docs/?pX20iA)
